# Late Quaternary range shifts of marcescent oaks unveil the dynamics of a major biogeographic transition in southern Europe

**DOI:** 10.1038/s41598-020-78576-9

**Published:** 2020-12-09

**Authors:** Carlos Vila-Viçosa, João Gonçalves, João Honrado, Ângela Lomba, Rubim S. Almeida, Francisco Maria Vázquez, Cristina Garcia

**Affiliations:** 1 CIBIO (Research Center in Biodiversity and Genetic Resources) - InBIO (Research Network in Biodiversity and Evolutionary Biology), University of Porto, Campus Agrário de Vairão, Rua Padre Armando Quintas, 4485-661 Vairão, Portugal; 2 MHNC-UP - Museum of Natural History and Science of the University of Porto - PO Herbarium, University of Porto, Praça Gomes Teixeira, 4099-002 Porto, Portugal; 3 Biology Department, Faculty of Sciences, University of Porto, Rua Do Campo Alegre, s/n, 4169-007 Porto, Portugal; 4 Agricultural Research Centre, Finca La Orden, Valdesequera, CICYTEX - Centro de Investigaciones Científicas Y Tecnológicas de Extremadura, Ctra. A-V, Km 372, 06187 Guadajira, Badajoz Spain; 5 Department of Evolution, Ecology and Behaviour, Institute of Integrative Biology (IIB), University of Liverpool, Bioscience Building, Liverpool, L69 7ZB UK

**Keywords:** Ecology, Biogeography, Conservation biology, Ecological modelling, Ecosystem ecology, Forest ecology, Ecology, Biodiversity, Biogeography, Conservation biology, Ecological modelling, Ecosystem ecology, Forest ecology

## Abstract

Marcescent forests are ecotones distributed across southern Europe that host increased levels of biodiversity but their persistence is threatened by global change. Here we study the range dynamics of these forests in the Iberian Peninsula (IP) during the Late Quaternary, a period of profound climate and anthropic changes. We modeled and compared the distribution of eight oak taxa for the present and two paleoclimatic environments, the Last Glacial Maximum (LGM, ~ 21 kya) and the Mid-Holocene (MH, ~ 6 kya). Presence records were combined with bioclimatic and topographic data in an ensemble modelling framework to obtain spatial projections for present and past conditions across taxa. Substantial distribution shifts were projected between the three studied periods, that were explained by precipitation, winter cold and terrain ruggedness. Results were congruent with paleoclimatic records of the IP and showed that range shifts of these contact zones concurred with range dynamics of both Submediterranean and Temperate oaks. Notably, the distribution ranges of hybrid oaks and marcescent forests matched throughout the late Quaternary. This study contributes to unveil the complex Late-Quaternary biogeography of the ecotone belt occupied by marcescent forests and, more broadly, of Mediterranean oaks. Improved knowledge of species’ responses to climate dynamics will allow us to anticipate and manage future range shifts driven by climate change.

## Introduction

Ecotones are transitional areas between contrasting adjacent ecological systems integrating ecological features from neighbor regions^1^. They host complex and highly diverse communities that combine endemic species and those from adjacent neighboring zones that typically use these areas as refugia when environmental changes challenge their persistence across their native ranges^2^. This combination of species makes ecotones hotspots of biodiversity and, therefore, they are of utmost importance if we are to preserve biodiversity in a changing world^3–5^ . Although the distribution and extent of ecotones have changed widely over millennia in response to climate change^6^, we still ignore whether and past climate changes have shaped the current distribution of species inhabiting ecotones.

Marcescent forests are ecotones located in the transition between temperate areas, with cold winters and mild rainy summers and Mediterranean ones, with dry and hot summers. They occur in the European Southern Peninsulas, through the Mediterranean Basin, including North Africa and Middle East regions^7,8^. Marcescence, i.e., the absence of leaf fall abscission, is a life-history trait characteristic of tree species from ecotones between contrasted climatic zones, such as those across Temperate and Mediterranean Europe^8,9^. Leaf retention was firstly interpreted as an adaptation to cold climates that would allow a direct uptake of soil minerals back to the tree^10^. Further, marcescence proved to be adaptive in areas with summer drought and winter frosts by protecting leaf buds from desiccation and extreme cold^11^. Despite the unclear adaptative value of marcescence, leaf retention is a trait highly responsive to environmental conditions that control distributional range fluctuations in response to climate changes^12^.

Previous biogeographic studies in the Iberian Peninsula, a well-known hotspot of European and global biodiversity, confine the distribution of marcescent woodlands to the submediterranean bioclimatic belt, where these forests are fed by moderate summer precipitation, at least during one month of the dry season^2,8,11^. Such mild summers allow marcescent forests to host warm-temperate and even sub-tropical species^13^, acting as refugia for paleoclimatic relicts, including rare taxa that are otherwise common in the Atlantic islands^14^. Model projections show that future climatic changes will likely hard-hit marcescent forests^11^ and therefore, anticipating distributional range shifts of marcescent species in response to future climate changes is an urgent research goal. Tracking past range shifts can also improve our ability to forecast future changes. In this sense, the severe climatic conditions exhibited during cold periods in the late Pleistocene triggered massive migrations of the European flora towards the southern peninsulas, where they become confined. The Last Glacial Maximum (LGM, *ca.* 20 Ky) was one of the most critical periods of the Quaternary for forest persistence because of their low temperatures and water availability^15^. Then, during the Bolling-Allerod period, the ice sheets retreated and the onset of the temperate and moister climate and mild winters triggered the expansion of Temperate and Mediterranean forest at the start of the late glacial (13 to 10 Ky), interrupted during the Younger Dryas (12.9 to 11.7 Ky). These forests recovered and achieved their maximum extent at the Early to Middle Holocene (*ca*. 6 Ky) depending on the geographical location^16^.

The magnitude of the distribution range shifts that marcescent forests have undergone in response to past climate changes is still poorly understood. We currently ignore the biogeographic trajectories that co-occurring oak species inhabiting this ecotone experienced in response to climate changes during the Late Quaternary. This is partly because most studies aimed to delineate past distributional ranges are based on fossil pollen records that provide a broad depiction of plant communities but fail to distinguish between deciduous oak species^17,18^. Recent approaches based on species distribution modeling (SDM) can provide new insights to advance our understanding of the biogeographic past trajectories^19–21^. This is especially important to transiently co-occurring oak species at the boundary of two biogeographic regions in Southern Europe. This study aims to elucidate the late-Quaternary dynamics of the Temperate-Mediterranean transition in southern Europe by applying correlative SDMs to hindcast the past distribution of eighth oaks (seven marcescent and one deciduous) distributed across the Iberian Peninsula as model taxa. Specifically, we:

(i) Applied correlative species distribution models to identify the main climatic factors determining the current distribution ranges for each studied species;
(ii) Hindcasted projections of each species’ past distribution and quantify distribution range shifts in response to climate changes;
(iii) Tracked the distribution shifts of oaks distributed across the submediterranean belt in the Iberian Peninsula based on the SDM's, and compare these shifts to those observed for temperate and mediterranea oak species; and
(iv) Compared current and past distributional ranges of parental species with the known occurrence of hybrids to infer ancient contact zones and the persistence or turn-over of hybrid-species.

We further discuss the implications of our results for preserving the biodiversity of marcescent forests that occupy the Temperate-Mediterranean transition in southern Europe in the face of projected climate change.

## Results

### Model performance and selection of environmental predictors

Selected models showed high performance scores for all species (Table 1), especially for *Quercus robur*, *Q. canariensis* and their hybrids. Model performance values (for details see Material and Methods section: *Modelling approach, model fitting and evaluation*) show that the lowest TSS values (True skill statistic bounded between [-1, 1], with values closer to one depicting better models) corresponded to *Q. faginea* (0.79) and *Q. broteroi* (0.77) up to *Q. robur* (0.98) which shows overall good to excellent model performance. The AUC (Area Under the Curve: [0, 1], values closer to one depict better models) were always above 0.96, also suggesting an excellent performance of the models. In addition, sensitivity and specificity scores (bounded between [0, 100]) were always above 85, confirming good and well-balanced performance of the models.

**Table 1:** Evaluation scores per species for the ensemble of 21 top-models (TSS: True skill statistic; AUC: Area Under the Curve; Sensitivity also called true positive rate or recall; Specificity also defined as the true negative rate).

Species	TSS	AUC	Sensitivity	Specificity
*Quercus robur*	0.98	1.00	98.88	99.09
*Quercus canariensis*	0.94	0.99	100.00	94.01
*Quercus* × *coutinhoi*	0.93	0.99	97.67	95.53
*Quercus* × *marianica*	0.91	0.99	97.67	93.34
*Quercus lusitanica*	0.90	0.99	95.65	94.72
*Quercus estremadurensis*	0.88	0.98	95.52	92.66
*Quercus faginea*	0.79	0.97	85.71	92.87
*Quercus broteroi*	0.77	0.96	89.20	87.50

Bioclimatic predictors showed the highest importance scores when compared to topographic ones (Supplementary Fig. S1). Exceptions were found for *Q. faginea* and *Q. canariensis*, for which the average topographic ruggedness index (TRI_AVG) was the most important variable. The contribution of variables related to the precipitation regime contributed the most to explain the present distribution of the focal species (Supplementary Fig. S1), especially the precipitation of the warmest quarter (BIO_18), the annual precipitation (BIO_12), and precipitation seasonality (BIO_15). Winter cold temperatures also held substantial explanatory power, especially the Annual mean temperature (BIO_01), the Mean Temperature of Coldest Quarter (BIO_11), and Isothermality (BIO_03).

### Changes in predicted distributions of marcescent oak species and their hybrids

**Figure 1:**
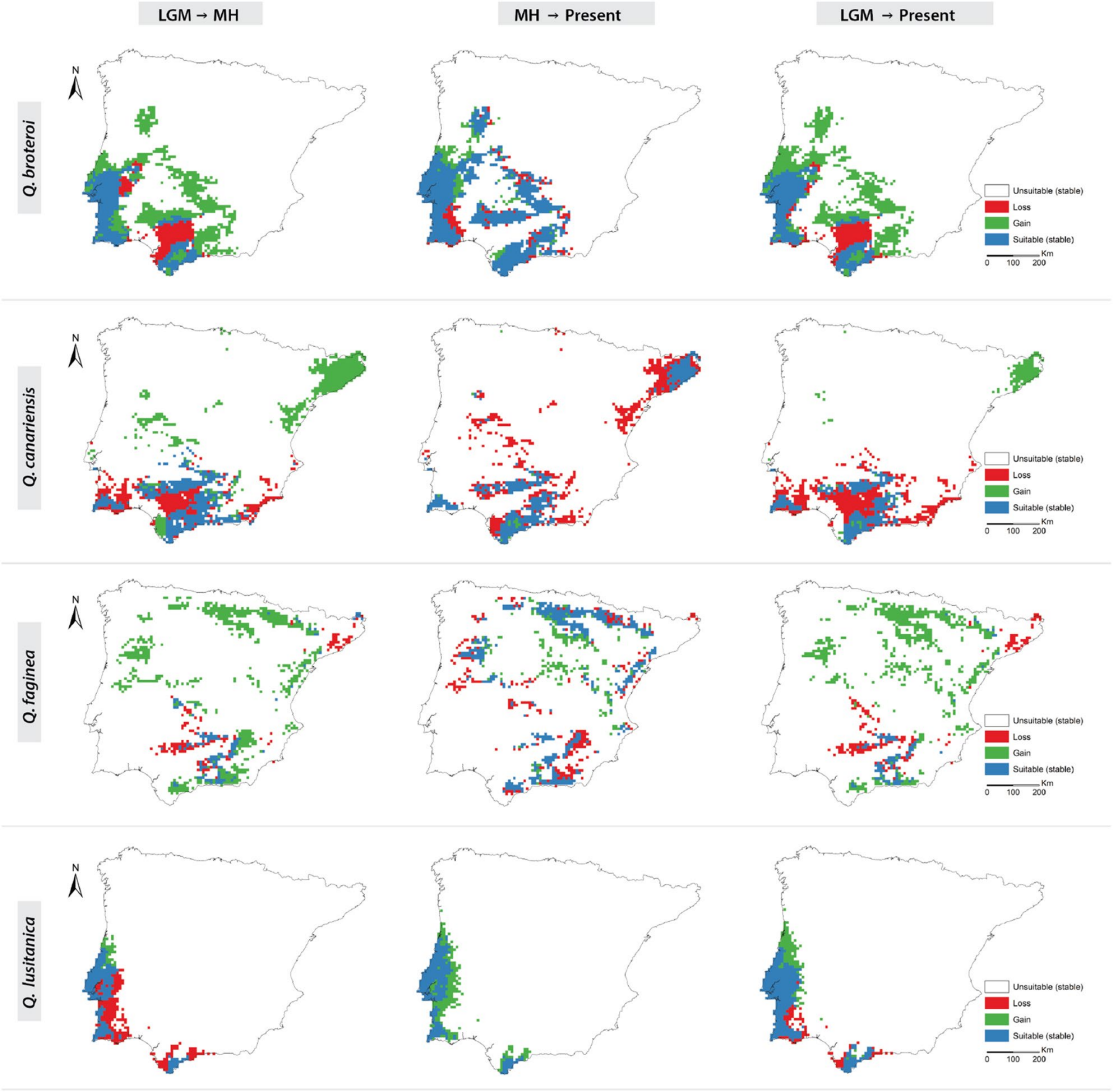
Changes in predicted distributions (gained, lost, or stable) for *Galliferae* oak species for the studied periods: LGM-MH (left); LGM-Present (center); MH-Present (right). Maps were generated by JG and CVV in R v.4.0.3 (https://www.r-project.org) and assembled in ArcMap 10.5 (https://www.esri.com/en-us/arcgis).

**Figure 2:**
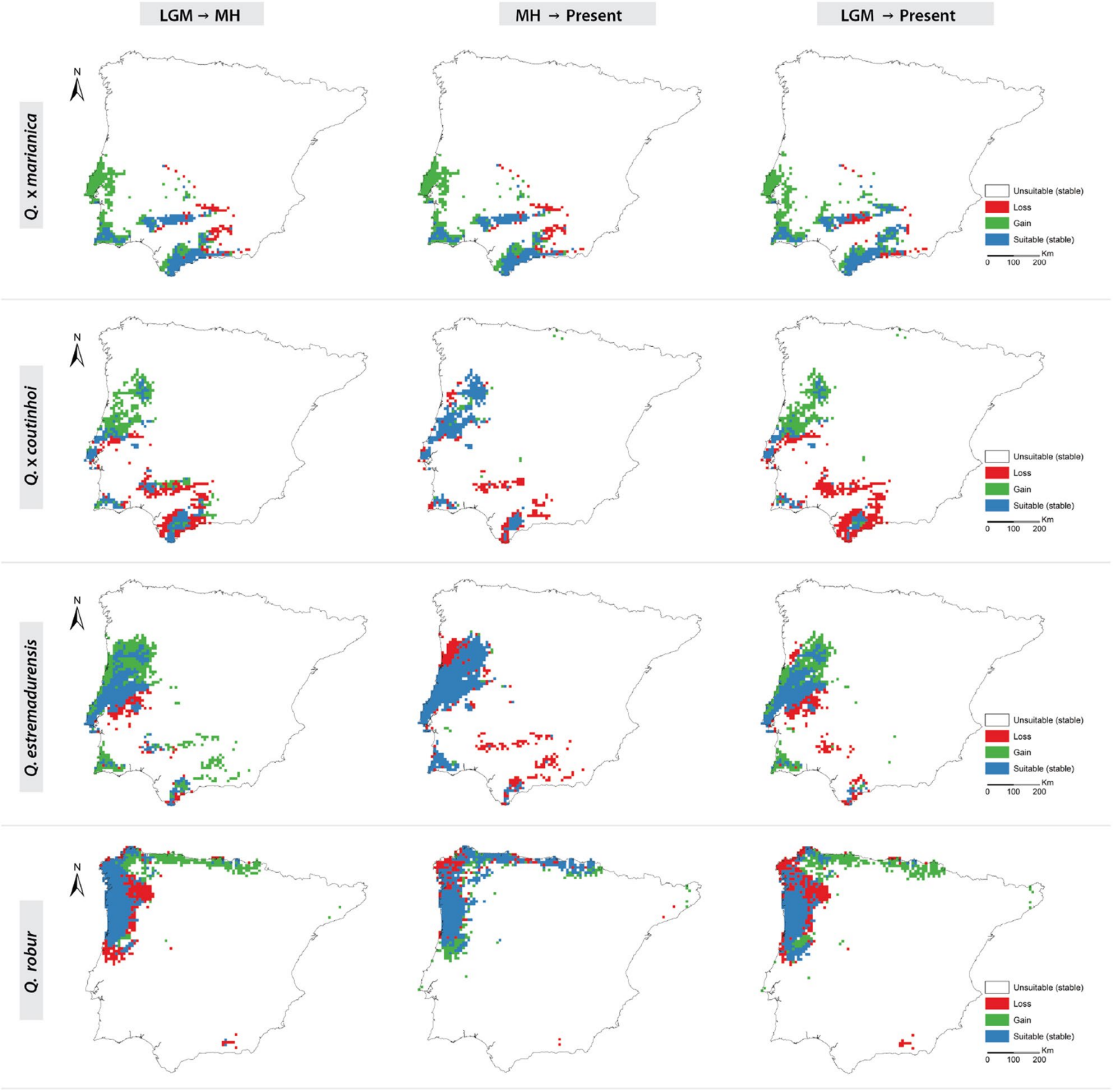
Changes in the predicted distributions (gained, lost, or stable) of hybrids (*Q* x *marianica* and *Q.* × *coutinhoi*) *and* Roburoid oaks (*Q. estremadurensis* and *Q. robur*) for the studied periods: LGM-MH (left); MH-Present (center); LGM-Present (right). Maps were generated by JG and CVV in R v.4.0.3 (https://www.r-project.org) and assembled in ArcMap 10.5 (https://www.esri.com/en-us/arcgis).

**Figure 3:**
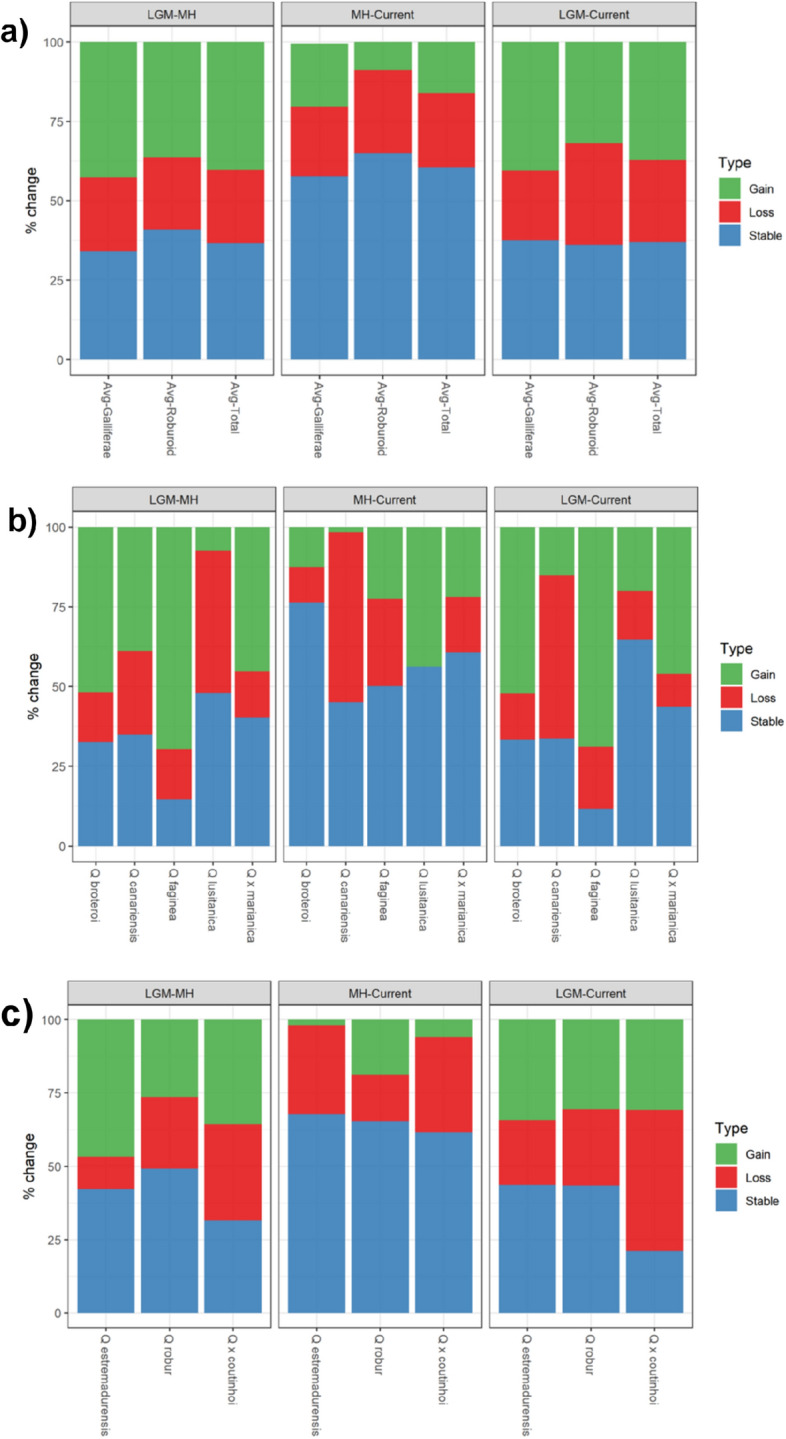
Percentage of change (% change) of the predicted distributions (gained, lost, or stable) in the focal time frames: LGM-HM (left), MH-Present (center) and LGM-Present (right) (**a**) Broad taxonomic groups (*Galliferae*, Roburoid, and Total); (**b**) Individual species within the *Galliferae* group; (**c**) Individual species within the Roburoid group and the hybrid *Q. x coutinhoi*. Graphics were generated by JG and CVV in R v.4.0.3 (https://www.r-project.org).

When taking into account all taxa pat-present dynamics (Fig. 3a), we observed an average loss of suitable areas from the LGM to the present of ca. 25%, offset by an overall gains of *ca*. 38% while about 37% of the range remained stable. From the MH to the present, all selected oak species maintained 60% of their area, with ca. 16% of gains and ca. 23% of losses in terms of suitable area (Figs. 1, 2 and 3).

**Figure 4:**
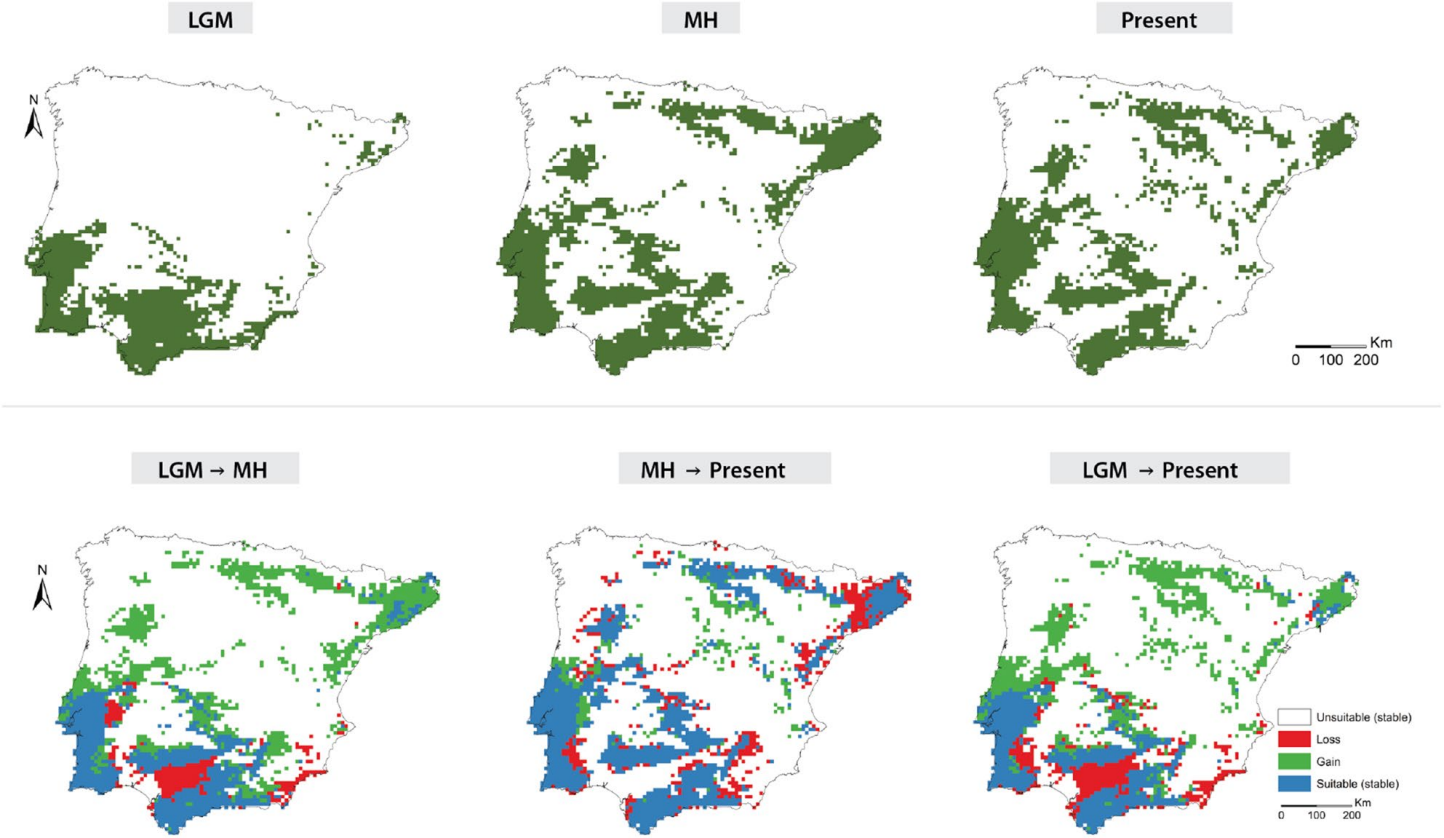
Predicted distribution of the submediterranean belt in Iberian Peninsula, based in the distribution of the strictly marcescent oaks (*Q. broteroi, Q. canariensis, Q. faginea* and *Q. marianica*) (top row) and their distributional range changes across the three studied periods (bottom row). Maps were generated by JG and CVV in R v.4.0.3 (https://www.r-project.org) and assembled in ArcMap 10.5 (https://www.esri.com/en-us/arcgis).

Considering taxonomic groups, we observed that subsection *Galliferae* species expanded 43% of their overall area from LGM to MH, with losses of ca. 23% and 34% of their range remaining stable (Figs. 2 and 4). Since the MH to the present, the range of these species remained stable for the most part (58%) (Figs. 1, 2, 3 and 4). Regarding roburoid oaks, they showed limited latitudinal shifts, while the overall extension of their distribution area remained generally stable (Figs. 2 and 3).

Regarding individual species, *Quercus broteroi* had an expansion of 52% from LGM to the present, expanding from southern basins and mountains, towards the Central Iberian Peninsula and the Northern basins. This species showed the highest stability in its distribution range from MH to the present (76%) (Figs. 2 and 3). In contrast, the distribution ranges of *Q. canariensis* contracted from LGM to the present (51%) with a fast-pace contraction from MH to present. Yet, this species experienced a notorious expansion of 39% towards the northeastern part of the Iberian Peninsula from LGM to MH (Figs. 1 and 3). *Q. faginea* had the highest range expansion from LGM to MH (70%), mainly from Southern and Eastern Iberian Peninsula to the Northern Mountains, Central System and both Douro and Tagus basins, while 50% of the distribution area remained generally stable from MH to the present. The distribution range of *Q. lusitanica* contracted 45% from LGM to MH followed by an expansion of 44% from MH to present (Figs. 1 and 3) when the species did not contract its distribution area. The distribution of *Q. estremadurensis* expanded (46%) from LGM to MH but ca. 40% of peripheral locations disappeared from MH to present. Finally, the distribution of *Q. robur* remained stable, particularly from the MH to the present (65%) (Figs. 2 and 3).

As for hybrids, changes in the distribution range of *Q.* × *marianica* were very similar to those observed for the parental *Q. broteroi* (Fig. 2). Changes in the distribution of *Q.* × *coutinhoi* resembled those observed for *Q. estremadurensis* expanding ca. 40% northwards and contracting ca. 30% in the southern areas (Figs. 2 and 3)*.*

Complementarily, the hierarchical clustering analysis, reflects the similarity of species projections in the three studied periods (Supplementary Fig. S3).

### Identification of contact zones

**Figure 5:**
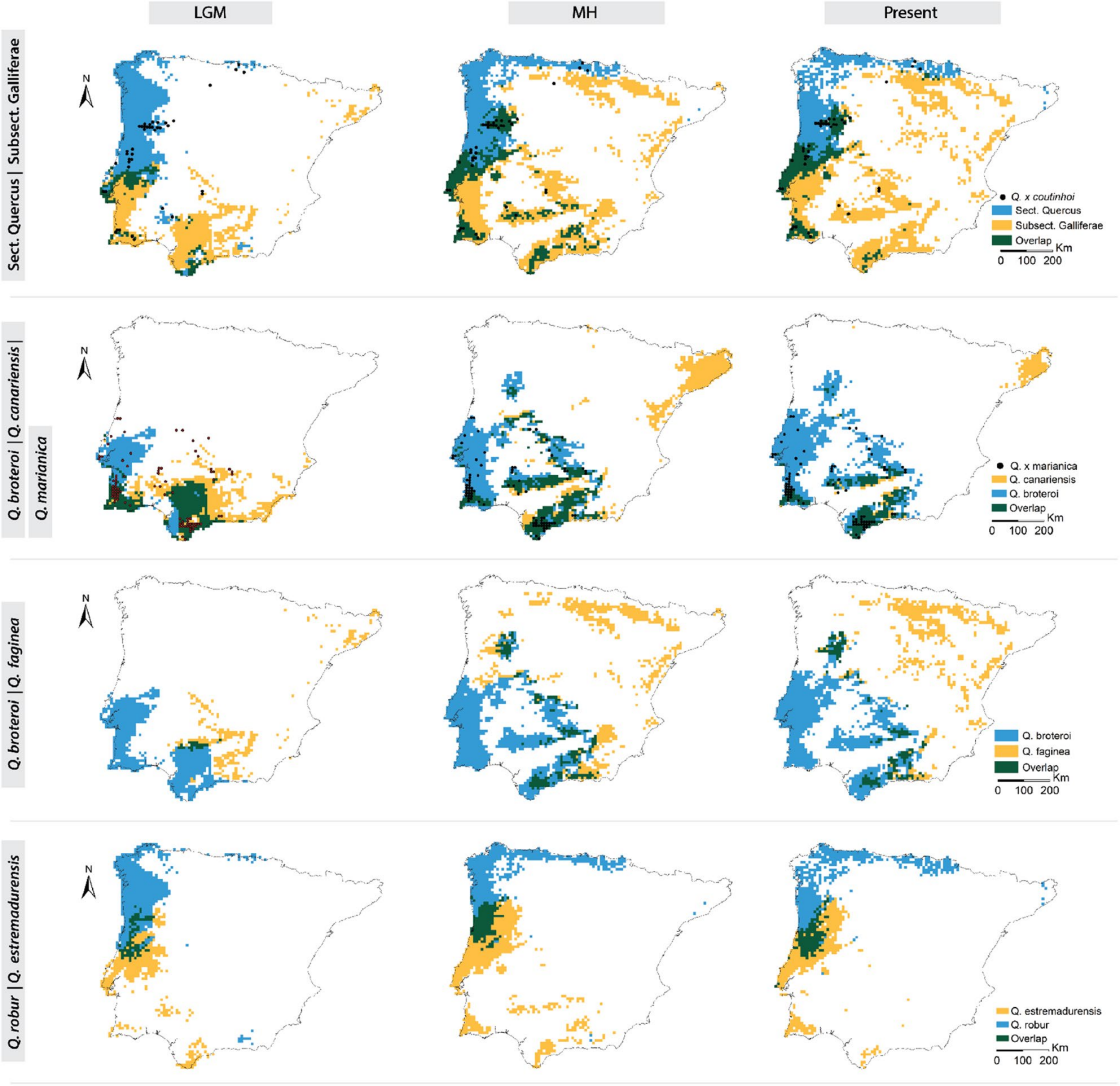
Distribution of the contact zones (green) of main targeted taxa across time periods. LGM (left), MH (center), and the present (right), and current distribution of hybrids (*Q.* × *coutinhoi* and *Q.* × *marianica*). Sect. *Quercus* refers to the roburoid oaks (*Q. robur* and *Q. estremadurensis*) and Subsection *Galliferae* refers to the Gall oaks (*Q. broteroi* and *Q. faginea*) (Colours were chosen in way to enhance visual discrimination of different groups and their overlap). Maps were generated by JG and CVV in R v.4.0.3 (https://www.r-project.org) and assembled in ArcMap 10.5 (https://www.esri.com/en-us/arcgis).

The contact zone between species of the two major groups (Roburoid oaks – Section *Quercus vs*. *Gall oaks* – Subsection *Galliferae*) was located across the Tagus basin in the LGM (with ca. 11% of overlap between distribution areas) and it expanded North in the MH, followed by a contraction of its area (ca.20%) towards the present (Figs. 3 and 5). The current distribution of *Q.* × *marianica* tracks the distribution of its parental species (*Q. canariensis* and *Q. broteroi*) that were predicted to co-exist during the LGM and MH periods mainly across southern and western areas (Figs. 2 and 5).

The distribution areas of *Q. broteroi* and *Q. faginea* showed a residual overlap (3%) in the LGM located in southern Spanish mountains. This contact zone expanded in the MH to reach 12% from the expansion of *Q. faginea* towards North, which originated a second contact zone in the Douro basin (Figs. 1 and 5). The distribution of the two roburoid oaks (*Q. robur* and *Q. estremadurensis*) also overlapped in the central and northern regions of Portugal. The area of this contact zone increased from 9 to 12% from LGM to the MH period and it remains generally stable since then (Figs. 2 and 5).

## Discussion

Our results reveal substantial distribution range shifts across Iberian Peninsula during the latest 20 ky for all studied species, suggesting a rather dynamic transition zone between two major biogeographic regions in Europe (Eurosiberian and Mediterranean). Overall, changes in precipitation and temperature during the late Quaternary were the main drivers of inferred distributional ranges. This also implies that future projected changes in precipitation regime and water balance will likely have strong effects on species ranges, thus calling for improvements in regional and local scenarios based on global climate models.

We complemented previous projections on Iberian trees [^22^] and other studies that underline a broad association of marcescent forests with the submediterranean bioclimatic belt [^2,8,11^]. In fact, while the distribution range obtained for the deciduous *Quercus robur* closely tracked the Eurosiberian region, the distribution ranges obtained for marcescent species (*Q. broteroi*, *Q. canariensis, Q. faginea* and *Q. marianica*) broadly corresponded to this submediterranean transition zone (Fig. 4).

Our models also revealed that the distribution range of marcescent forests included the western coast of the Iberian Peninsula, an area with frequent advection fogs during summer that might compensate low annual precipitation values [^11^], thus favoring the expansion and establishment of marcesncent forests. This overlooked source humidity has led previous biogeographic models to characterize *Q. broteroi* and *Q. canariensis* as thermophilic taxa inhabiting dry conditions [^8^]. On the contrary, they are maintained by frequent summer-fog regimes^23,24^ that increase annual and summer precipitation and confer a submediterranean character to these areas. Also, this climatic and topographic heterogeneities favoured by small mountain ranges play a crucial role as identified across major refugia areas throughout the Mediterranean Basin^25,26^.

The geographic patterns obtained for the different study periods are consistent with the known biogeography and dynamics of Iberian forests since the last glacial maximum (LGM)^16,27^. Overall, climatic hardiness and increased annual precipitation in Southwest Iberian Peninsula during the LGM allowed the persistence of most marcescent oaks in the southern range limits of their present distribution. The distributional segregation between marcescent oaks and the deciduous *Q. robur* (Figs. 1, 2 and Supplementary Fig. S3) is evidenced by the range contraction of the latter between the LGM and MH, from the inland towards the Atlantic and Cantabrian coasts. This coastal expansion follows an increase in winter minimum temperatures, while the inland retreats are a response to increasing summer drought (i.e. more Mediterranean climate). Other roburoid oaks (*Q. estremadurensis* and *Q.* × *coutinhoi*) and *Q. lusitanica* follow the same general pattern.

The climatic amelioration during the MH explains most of the expansion of marcescent oaks towards Center and North Iberian Peninsula, followed by a slight contraction as the climate became drier towards the present. The present distribution of *Quercus faginea* approaches the roburoid taxa, unlike the remaining *Galliferae* oaks (Fig. 4 and Supplementary Fig. S3). This is related with this species recent expansion through the Eurosiberian region, where it forms secondary forests in locally drier biotopes, in areas where the more favorable soils are occupied by deciduous primary forests of *Fagus sylvatica, Q. petraea* and *Q. pubescens*^28,29^. Terrain ruggedness followed by summer precipitation plays a major role on *Q. faginea* and *Q. canariensis* distributions (Fig. 1 and Supplementary Fig. S1). Both are associated with increased summer precipitation, with the first expanding through interior mountainous areas, which is confirmed by the influence of precipitation and temperature seasonality, while *Q. canariensis* distribution is determined by a summer fog regime both in coastal and inland areas (Fig. 1 and Supplementary Fig. S1). The less fragmented distribution of *Q. canariensis* during the LGM, across southern Iberia, discloses an ancient contact between nowadays relict populations. This contact is supported by the current presence of vicariant assemblages of understory species, including relict taxa like *Frangula baetica, Cytisus baeticus, Davallia canariensis, Rhododendron ponticum* and *Myrica* spp.^30^.

**Figure 6:**
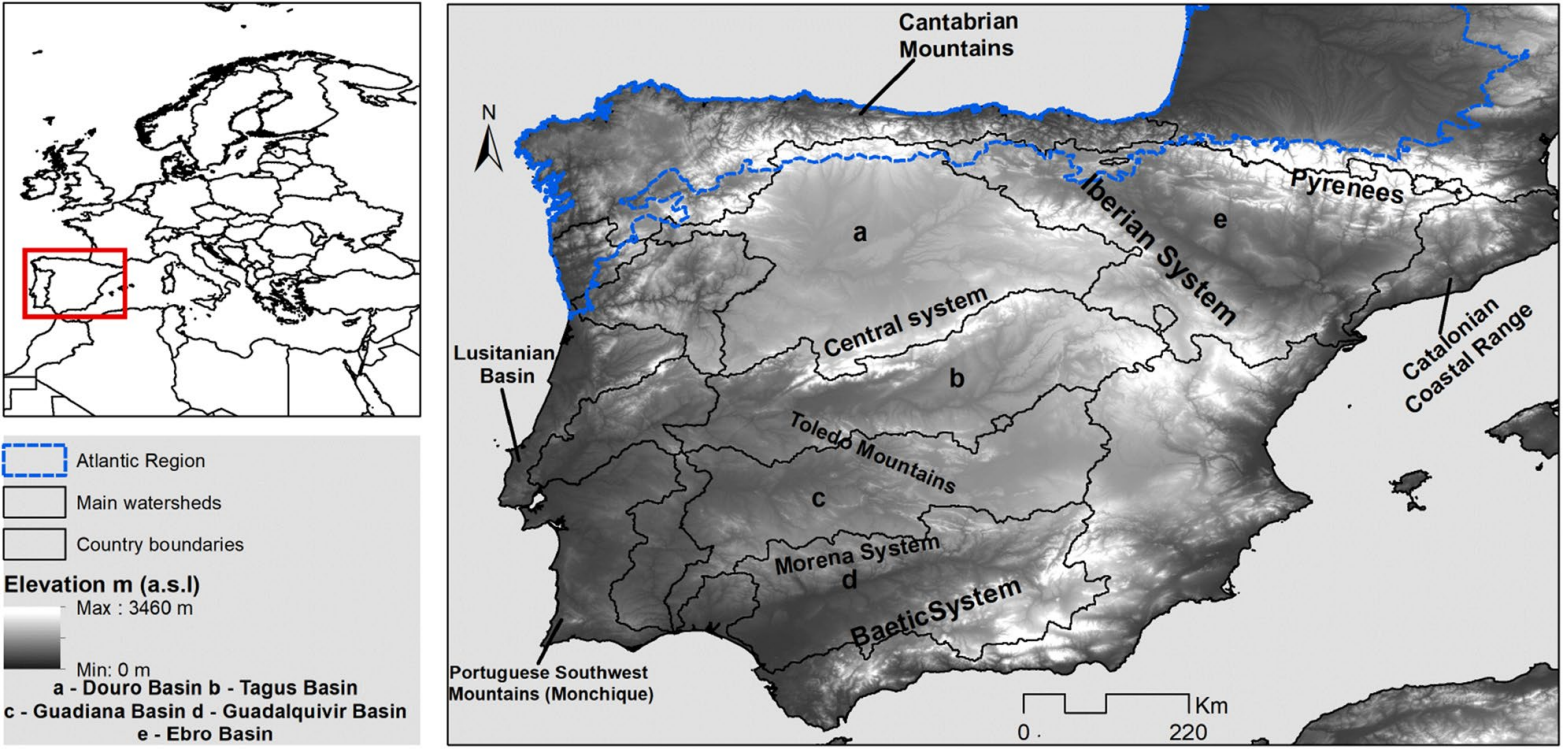
Study area (Iberian Peninsula) in the European context (left) and with the boundary of the Atlantic region highlighted in blue (right) and major river basins and mountain systems. Source: EEA. (European Environment Agency, Copenhagen, Denmark) 2012.

Our results support ancient, but not current, sympatry between parental species during the LGM and MH at locations where their hybrids are currently present (Fig. 5). The distribution of *Q.* × *marianica* tracks the distribution of *Q. canariensis* in the LGM but it progressively tracks the distribution range of its other parental species (*Q. broteroi*) during the MH and towards the present (Fig. 5 and Supplementary Fig. S3). This shows both adaptation towards drier conditions in face of *Q. canariensis* and expansion towards equilibrium considering the parental niches. Similarly, the spatial distribution of roburoid (*Q. estremadurensis* and *Q. robur*) and *Galliferae* parental species (*Q. broteroi* and *Q*. faginea) closely resembles the current distribution of the hybrid *Q.* × *coutinhoi* towards northern areas (Figs. 2 and 5). Here, *Q. robur* may have shifted between submediterranean and temperate areas, benefitting from less severe summers and winters during the Holocene^31^, which may explain the detected hybrid swarms during fieldwork in the Douro Basin and Central Portugal (Fig. 6), in populations where roburoid oaks are absent.

The species-level segregation of *Q. broteroi* and *Q. faginea* is congruent with their exiguous overlap during the LGM, followed by the posterior expansion (MH) of *Q. faginea* to NW Iberia (Figs. 1 and 5). The distribution of *Quercus lusitanica* is generally closer to roburoid oaks, especially in the LGM, but approaching other *Galliferae* species towards the present (Fig. 4 and Supplementary Fig. S3).

The projected distribution of *Q. estremadurensis* (Fig. 2) is consistent with its thermophilic preferences and resembles the one obtained for *Q. canariensis* (Fig. 1). This strengthens the hypothesis of *Q. estremadurensis* being a Tertiary relict oak that evolved under a subtropical paleo-environment^32–34^. Herbaria review performed by the authors confirms the presence of this roburoid oak in northern Africa where it co-exists with other relicts such as *Q. canariensis* and *Prunus lusitanica*. In contrast, the projected distribution of *Q. estremadurensis* is opposed to the well-established Eurosiberian distribution of *Q. robur *^35^. This projection dismisses any southern Iberian references of *Q. robur* enhancing the taxonomic dichotomy between these two roburoid oaks. Furthermore, the projected distribution of *Q. estremadurensis* in southwest Spain, emphasizes the role of Guadalquivir basin as a “dry” barrier for its expansion across southern Iberia (Figs. 2, 5 and 6).

## Conclusions

We provided insights into the late-Quaternary distribution range shifts of a set of oak species in response to past climate changes. By combining hindcast and current projections obtained from species distribution models, we provide novel insights to disentangle taxonomic uncertainties. The performance of the models and the major importance of climatic variables shows that these oaks are rather suitable model species to undergo paleo-environmental and biogeographic studies along transitional bioclimatic and biogeographic areas in southern Europe. Further, our methodology proved valid to infer the distributional range shifts of hybrid taxa that showed to be congruent with the biogeographic trajectories of their parental species. This is quite important in a group with low interspecific barriers to gene flow and, therefore, characterized by complex evolutionary and speciation patterns^36,37^. . Our work addresses a major gap in paleo-vegetation studies that typically rely on palinological data, which tend to fail in delimitating the distribution of tree species accurately, particularly in the case of deciduous oaks (Sect. *Quercus*)^17,18^. Our study suggests that conservation efforts should target *Q. canariensis* and *Q. estremadurensis* because both taxa presently show a scattered (known and projected) distribution. In this regard, species distribution models prove useful to forecast the responses of these species to future climatic scenarios will contribute to prioritize conservation efforts.

## Materials and methods

### Study area

The Iberian Peninsula, located in southwestern Europe and with a total area of 583,832 km^2^ (Fig. 6), holds a highly diverse geological history^38^ and a well-documented late Holocene climatic influence^39^. The present climate ranges from dry Mediterranean in the Southeast to wet Temperate Atlantic in the North^7^. The former is considered to have a Submediterranean variant that represents 65.64% of its total area in the peninsula, where at least in one summer month, the average of rainfall in millimeters is 2.8 times lower than the average temperature. Under these conditions marcescent forests constitute the natural potential vegetation^7,8,11^. Due to its high edaphoclimatic heterogeneity, the Iberian Peninsula is a major hotspot of European plant diversity, hosting 54% of all European species, with 22.7% percentage of endemicity, and a remarkable number of 21 endemic genera^40^.

### Focal taxa and occurrence data

The European white oaks (Section *Quercus*)^41^ include a group traditionally segregated as a subsection (*Galliferae* (Spach) Guerke)^9,42^ that contains a group of oak species, considered strictly marcescent, with a broad (though disjoint) distribution across the Mediterranean Basin, from the Middle East to the Western Mediterranean region^9^. *Five* taxa belonging to this group (*Quercus broteroi, Q. canariensis, Q. faginea, Q. lusitanica*, including one hybrid *Q.* × *marianica*) plus two roburoid oaks (*Q. estremadurensis* and *Q. robur*) and the hybrid between them and the previous group (*Q.* × *coutinhoi*) were selected as focal taxa totaling eight taxa (Supplementary Note S1, Fig. S2).

Occurrence data was gathered through extensive fieldwork in Western Iberian Peninsula (since 2005), plus literature and herbaria review (Supplementary Table S1). Specimens were assigned to each taxa based on the authors’ experience and supported in relevant literature^32,34,43–45^. To tackle taxonomic uncertainty, a thorough examination of more than 5000 vouchers of oak specimens was performed in 16 reference herbaria. This info was assessed and harmonized with other herbaria online collections and databases (Supplementary Information S1). Records for the target species (presence-only data) were aggregated to a 10 × 10 km grid (Supplementary Table S2).

### Environmental variables (predictors)

Model development was based on regional/coarse-scale factors potentially influencing the target species distribution, related to bioclimatic and topographic descriptors of the niche environmental space.

For bioclimatic data we used the WorldClim v.1.4 dataset (URL: http://worldclim.org/version1), containing 19 bioclimatic indices, available between for the period 1960–1990^46^. For obtaining model-based hindcasts, we used WorldClim’s downscaled paleoclimate reconstructions for the Last Glacial Maximum (LGM; *ca.* 22 Ky) and the Mid-Holocene (MH; *ca.* 6 Ky). WorldClim infers the mean values of bioclimatic variables of the LGM based on three Global Circulation Models (GCMs) whereas it uses nine GCMs to estimate those values for current distributions. Therefore we opted for calculating the average of all available models as to reduce uncertainties linked to each specific GCM^47^. All bioclimatic data were later re-projected and re-sampled (average) to a common reference grid at 10 km spatial resolution (Datum WGS1984/UTM30N).

For portraying the ‘static’ influence of terrain morphology, wetness and its spatial heterogeneity on species distributions, we used the Topographic Ruggedness Index (TRI; as a proxy of slope and terrain complexity) and the Topographic Wetness Index (TWI; as a proxy of soil moisture and flow accumulation) both calculated from the SRTM (v.4) elevation data at 100 m of spatial resolution. For quantifying both variables, we aggregated/upscaled TRI and TWI values from their original resolution using the average (TRI_AVG) and the standard-deviation (TWI_STD).

We performed the pre-selection of bioclimatic variables based on preliminary models, using an exhaustive approach (similar to the one suggested by Cobos^48^ which assessed all combinations of two variables for temperature and two variables for precipitation. This selection aimed to maximize per species model performance and parsimony (i.e. combinations with greater predictive performance where screened), balance variables linked to both temperature and precipitation, assess and prevent prediction issues related to non-analogous climatic conditions, and take advantage of previous knowledge regarding each species ecological requirements. Finally, six environmental variables were used for model fitting per species (Supplementary Table S3).

### Modelling approach, model fitting and evaluation

Species Distribution Models (SDMs) were developed using the *biomod2* R package, which applies a multi-technique ensemble forecasting approach to analyze species-environment relations and to obtain spatiotemporal predictions^49^. Models were fitted using ten modelling techniques: GLM (Generalized Linear Models); GBM (Generalized Boosted Models); GAM (Generalized Additive Models); CTA (Classification Tree Analysis); ANN (Artificial Neural Networks); FDA (Flexible Discriminant Analysis); MARS (Multivariate Adaptive Regression Splines); RF (Random Forests); MAXENT.Phillips and MAXENT.Tsuruoka (Maximum Entropy Models) currently available in *biomod2*. Default parameters were used (with the exception of the smoothing degree term in GAM for Q*. canariensis*, which was set to $k=2$ to avoid over-fitting issues^50^. We only counted for presence data for focal species and therefore we obtained ten sets of randomly generated pseudo-absences (PA), each one with ten times the number of presences to increment the representativeness of the environmental space of the study area^51^. Since no previous information was assumed about species prevalence ($p$), model weights were adjusted to $p=0.5$ (*biomod2* default) thus giving similar weight to presences and pseudo-absences. We also verified that all species (either narrow or widespread ones) had sufficient presence records to train models following guidelines in previous research^52,53^. Holdout cross-validation was employed to evaluate the models, with 80% of the input records used for model fitting and 20% for model evaluation at each round. Twenty rounds were performed for model evaluation (plus one additional ‘full calibration’ round which uses all input records). For assessing model performance, the Area Under the Receiver-Operating Curve (AUC), the True-skill Statistic (TSS), and the Sensitivity and the Specificity values were calculated in *biomod2*^49^.

Given that ${N}_{T}=2100$ models were fitted per species (with ${{N}_{T}=N}_{techniques }\times {N}_{PA\_sets} \times {N}_{evaluation\_rounds}$), the less performant models were filtered out before the final ensemble forecasting. Hence, we selected the top 21 models (the top 1% percentile) considering the AUC rank. Based on these top-performing models, an ensemble using the average was implemented, thus reducing inter-model uncertainty. To transform the predicted probabilities into binary predictions of suitable/unsuitable areas, we used a threshold minimizing the straight-line distance between the receiver operating curve and the upper-left corner of the unit square^54^. To assess the importance of each variable in model fitting, we used *biomod2*′s internal method that calculates 1 – Pearson’s correlation between reference predictions and predictions for a ‘randomized’ version of each variable. The highest the score the greater is the influence of a variable in model predictions. A value of zero assumes no influence of a given variable. Variable importance scores were averaged across the top-performing models.

### Evaluating species range shift and overlap across time

After fitting the models and obtaining ensemble predictions for present conditions, we used the LGM or MH paleoclimate reconstructions of WorldClim (see above) to obtain model hindcasts for these periods. Niche conservatism (i.e., “*(…) retention of niche-related ecological traits over time.*”^55^ was assumed which holds that models are reasonably transferable and thus allowing to obtain spatiotemporal projections for past reference frames (see e.g.^55–57^). Then, we performed a spatial overlap between present and past projections to evaluate shifts in species distributions. This allowed us to identify stable areas (that remained suitable or unsuitable between time periods), gains (areas that gained environmental suitability between periods) and losses (areas that lost environmental suitability between periods). Afterwards, we spatially stacked all species distribution maps and obtained a binary matrix by extracting and transposing map values, later used to compute the Sorensen distance (Sd). Based on the Sd distance matrix, we then performed the hierarchical cluster analysis using the complete linkage method for each time step^58^. This allowed to identify differences and similarities of distributions between species through time.

## Supplementary information

Supplementary Legends.

Supplementary Table S1.

Supplementary Table S2.

Supplementary Table S3.

Supplementary Fig. S1.

Supplementary Fig. S2.

Supplementary Fig. S3.

Supplementary Note S1.

Supplementary Information S1.

## Data Availability

The authors agree on sharing this study data and its deposit in public repositories, upon reasonable request.
