# Supplementary material for: Late Quaternary range shifts of marcescent oaks unveil the dynamics of a major biogeographic transition in southern Europe
Source: Sci Rep. 2020 Dec 9;10:21598. doi: 10.1038/s41598-020-78576-9 (PMC7726089; doi:10.1038/s41598-020-78576-9)
Supplement: Supplementary file 1 — Supplementary Legends. [file 41598_2020_78576_MOESM1_ESM.docx]

**Supplementary material**

**Table S1** – Focal taxa, with corresponding taxonomic filiation and number of available records at their native resolution (total records) and after aggregating into the 10x10 km resolution adopted for the modelling framework (standardized records).

**Table S2-** Geographic records (Coordinate System: Datum WGS1984 / UTM 30 N) (EPSG: 32630) aggregated into 10x10 km.

**Table S3** – Variables used in SDMs for each target species (BIO1 = Annual Mean Temperature; BIO3 = Isothermality; BIO6 = Min Temperature of Coldest Month; BIO7 = Temperature Annual Range; BIO11 = Mean Temperature of Coldest Quarter; BIO12 = Annual Precipitation; BIO15 = Precipitation Seasonality; BIO18 = Precipitation of Warmest Quarter; BIO19 = Precipitation of Coldest Quarter; TRI_AVG = Topographic Ruggedness Index average and TWI_STD = Topographic Wetness Index standard-deviation).

**Figure S1 -** Importance scores of environmental variables obtained from biomod2 *per* selected species BIO_01 = Annual Mean Temperature; BIO_03 = Isothermality; BIO_06 = Min Temperature of Coldest Month; BIO_07 = Temperature Annual Range; BIO_11 = Mean Temperature of Coldest Quarter; BIO_12 = Annual Precipitation; BIO_15 = Precipitation Seasonality; BIO_18 = Precipitation of Warmest Quarter; BIO_19 = Precipitation of Coldest Quarter; TRI_AVG – Topographic Ruggedness Index average and TWI_STD Topographic Wetness Index standard-deviation.

**Figure S2 –** Distribution of the focal taxa in Iberian Peninsula

**Figure S3 -** Changes in predicted distributions of marcescent oak species and their hybrids, based in the Hierarchical cluster analysis (Sorensen Distance) obtained by the species distribution similarities for the three studied periods (LGM, MH and Present).

**Supplementary Note S1 -** Focal taxa explanation.

**Supplementary Information S1 –** Herbaria list of studied physical and virtual collections (*Quercus* L) and consulted databases.
