# Supplementary material for: Late Quaternary range shifts of marcescent oaks unveil the dynamics of a major biogeographic transition in southern Europe
Source: Sci Rep. 2020 Dec 9;10:21598. doi: 10.1038/s41598-020-78576-9 (PMC7726089; doi:10.1038/s41598-020-78576-9)
Supplement: Supplementary file 2 — Supplementary Table S1. [file 41598_2020_78576_MOESM2_ESM.pdf]

Table S1– Focal species, their corresponding taxonomic filiation and number of available records at their native resolution (total records) and after aggregating into the 10x10 km resolution adopted for the modelling framework (standardized records).

Species	Section / Subsection	Total records	Standardized Records (10 x 10 km)
Q. broteroi	Galliferae (Spach) Guerke (Gürke, (1897: 68))	402	400
Q. canariensis	Galliferae (Spach) Guerke (Gürke, (1897: 68))	44	42
Q. ×coutinhoi	Interseccional hybrid (Sect. *Quercus* vs Subsection *Galliferae*)	43	41
Q. estremadurensis	Section Quercus (Denk et. al. (2017: 13)	67	67
Q. faginea	Galliferae (Spach) Guerke (Gürke, (1897: 68))	315	315
Q. lusitanica	Galliferae (Spach) Guerke (Gürke, (1897: 68))	140	140
Q. ×marianica	Galliferae (Spach) Guerke (Gürke, (1897: 68))	86	86
Q. robur	Section Quercus Denk et. al. (2017: 13)	2549	548
		**Total: 3646**	**Total 1639**
