# Supplementary material for: Late Quaternary range shifts of marcescent oaks unveil the dynamics of a major biogeographic transition in southern Europe
Source: Sci Rep. 2020 Dec 9;10:21598. doi: 10.1038/s41598-020-78576-9 (PMC7726089; doi:10.1038/s41598-020-78576-9)
Supplement: Supplementary file 3 — Supplementary Table S2. [file 41598_2020_78576_MOESM3_ESM.pdf]

Table S2 - Records for the target species (presence-only data) aggregated to a 10×10km grid

Coordinate System: Datum WGS1984 / UTM 30 N

(EPSG: 32630)

Species	x	y
Quercus broteroi	-56906,32	4304323,32
Quercus broteroi	-56247,26	4314326,84
Quercus broteroi	-46878,31	4303737,56
Quercus broteroi	-46223,30	4313739,95
Quercus broteroi	-38838,00	4273051,80
Quercus broteroi	-38182,46	4283052,57
Quercus broteroi	-36867,27	4303054,05
Quercus broteroi	-36207,63	4313054,75
Quercus broteroi	-28824,31	4272384,83
Quercus broteroi	-28172,67	4282384,52
Quercus broteroi	-27510,99	4292383,62
Quercus broteroi	-54916,85	4334444,89
Quercus broteroi	-45566,86	4323742,31
Quercus broteroi	-44900,31	4333744,04
Quercus broteroi	-43580,29	4353748,62
Quercus broteroi	-42909,49	4363750,27
Quercus broteroi	-42245,92	4373752,49
Quercus broteroi	-35555,29	4323056,01
Quercus broteroi	-34892,90	4333056,67
Quercus broteroi	-34229,15	4343057,30
Quercus broteroi	-32230,73	4373170,75
Quercus broteroi	-31561,51	4383171,29
Quercus broteroi	-25544,18	4322382,55
Quercus broteroi	-24877,27	4332381,58
Quercus broteroi	-24217,72	4342381,17
Quercus broteroi	-23549,42	4352491,84
Quercus broteroi	-22878,59	4362490,81
Quercus broteroi	-22215,10	4372490,35
Quercus broteroi	-21550,30	4382489,87
Quercus broteroi	-20884,19	4392489,37
Quercus broteroi	-20216,78	4402488,85
Quercus broteroi	-17193,92	4140904,40
Quercus broteroi	-13991,16	4191001,29
Quercus broteroi	-12704,22	4210995,56
Quercus broteroi	-6546,08	4150287,24
Quercus broteroi	-5911,41	4160283,37
Quercus broteroi	-5266,72	4170278,96
Quercus broteroi	-4629,69	4180275,09
Quercus broteroi	-3982,67	4190270,68
Quercus broteroi	-3343,29	4200266,80
Quercus broteroi	2818,79	4139579,35
Quercus broteroi	3457,92	4149573,95
Quercus broteroi	4104,80	4159679,63
Quercus broteroi	4746,17	4169674,23
Quercus broteroi	5379,83	4179669,36
Quercus broteroi	6023,45	4189663,97

Quercus broteroi	13468,16	4148983,86
Quercus broteroi	14752,06	4168970,62
Quercus broteroi	15391,19	4178964,28
Quercus broteroi	16037,91	4189069,01
Quercus broteroi	21567,64	4118317,27
Quercus broteroi	22838,86	4138302,11
Quercus broteroi	23471,56	4148294,81
Quercus broteroi	24120,51	4158398,08
Quercus broteroi	25399,88	4178383,02
Quercus broteroi	32851,45	4137737,15
Quercus broteroi	33489,82	4147728,44
Quercus broteroi	42857,50	4137073,19
Quercus broteroi	43492,66	4147063,62
Quercus broteroi	44128,74	4157054,07
Quercus broteroi	52223,35	4126432,20
Quercus broteroi	52854,46	4136421,77
Quercus broteroi	53495,31	4146410,90
Quercus broteroi	61587,22	4115805,35
Quercus broteroi	62223,26	4125793,63
Quercus broteroi	-18811,10	4271730,47
Quercus broteroi	-18154,62	4281728,56
Quercus broteroi	-7491,95	4291082,89
Quercus broteroi	-2045,39	4220369,59
Quercus broteroi	-1393,71	4230365,16
Quercus broteroi	3833,17	4310442,95
Quercus broteroi	7960,96	4219647,81
Quercus broteroi	8609,01	4229642,43
Quercus broteroi	9264,89	4239748,13
Quercus broteroi	11863,83	4279727,18
Quercus broteroi	13182,97	4299715,93
Quercus broteroi	13839,81	4309710,57
Quercus broteroi	18618,12	4229043,21
Quercus broteroi	19910,17	4249030,62
Quercus broteroi	20566,52	4259023,82
Quercus broteroi	22530,97	4289115,57
Quercus broteroi	23191,47	4299108,80
Quercus broteroi	28620,33	4228345,35
Quercus broteroi	29920,23	4248441,54
Quercus broteroi	30572,83	4258433,87
Quercus broteroi	31226,41	4268426,21
Quercus broteroi	33193,03	4298403,31
Quercus broteroi	33850,52	4308395,71
Quercus broteroi	39275,64	4237762,43
Quercus broteroi	39923,53	4247753,90
Quercus broteroi	42533,20	4287719,47
Quercus broteroi	43854,34	4307813,18
Quercus broteroi	49282,50	4237088,10
Quercus broteroi	51888,27	4277160,96
Quercus broteroi	52544,71	4287151,22
Quercus broteroi	61888,48	4276495,54

Quercus broteroi	62541,02	4286485,05
Quercus broteroi	63203,09	4296474,12
Quercus broteroi	71888,57	4275842,73
Quercus broteroi	72545,94	4285831,05
Quercus broteroi	-14870,75	4331719,93
Quercus broteroi	-14206,73	4341717,94
Quercus broteroi	-12876,29	4361825,59
Quercus broteroi	-12208,52	4371823,56
Quercus broteroi	-11539,50	4381821,52
Quercus broteroi	-10869,25	4391819,47
Quercus broteroi	-10206,35	4401817,97
Quercus broteroi	-3535,15	4351064,64
Quercus broteroi	-2865,76	4361172,76
Quercus broteroi	-2193,71	4371169,22
Quercus broteroi	-1529,10	4381166,23
Quercus broteroi	-863,30	4391163,24
Quercus broteroi	-196,31	4401160,24
Quercus broteroi	471,87	4411157,23
Quercus broteroi	7137,33	4360421,82
Quercus broteroi	8480,90	4380524,00
Quercus broteroi	9150,86	4390519,55
Quercus broteroi	9813,34	4400515,64
Quercus broteroi	10485,55	4410511,19
Quercus broteroi	15823,71	4339805,61
Quercus broteroi	16484,91	4349800,27
Quercus broteroi	17147,19	4359794,94
Quercus broteroi	19149,11	4389778,42
Quercus broteroi	19815,69	4399773,10
Quercus broteroi	20483,34	4409767,78
Quercus broteroi	25161,74	4329089,59
Quercus broteroi	25826,33	4339082,85
Quercus broteroi	29824,74	4399154,76
Quercus broteroi	30496,43	4409148,09
Quercus broteroi	37822,48	4368461,45
Quercus broteroi	38486,76	4378453,95
Quercus broteroi	39160,61	4388445,94
Quercus broteroi	40493,94	4408431,01
Quercus broteroi	44517,44	4317804,28
Quercus broteroi	50506,48	4407837,65
Quercus broteroi	54519,23	4317122,16
Quercus broteroi	55170,45	4327113,00
Quercus broteroi	55831,20	4337103,39
Quercus broteroi	64512,24	4316453,29
Quercus broteroi	65176,70	4326442,44
Quercus broteroi	67157,51	4356411,02
Quercus broteroi	75174,20	4325785,16
Quercus broteroi	75835,30	4335773,65
Quercus broteroi	76497,13	4345762,18
Quercus broteroi	1149,80	4421153,66
Quercus broteroi	3854,89	4461252,08

Quercus broteroi	4530,16	4471249,03
Quercus broteroi	11833,32	4430502,28
Quercus broteroi	12508,87	4440497,82
Quercus broteroi	13177,00	4450493,91
Quercus broteroi	13854,79	4460489,45
Quercus broteroi	14533,69	4470484,99
Quercus broteroi	21837,38	4429867,71
Quercus broteroi	22516,78	4439861,88
Quercus broteroi	23188,69	4449856,58
Quercus broteroi	23861,66	4459851,29
Quercus broteroi	25219,29	4479840,18
Quercus broteroi	31169,13	4419141,43
Quercus broteroi	31842,82	4429134,78
Quercus broteroi	32517,50	4439128,14
Quercus broteroi	33184,65	4449122,04
Quercus broteroi	33861,34	4459115,42
Quercus broteroi	34545,89	4469219,89
Quercus broteroi	41170,61	4418423,05
Quercus broteroi	41846,33	4428526,70
Quercus broteroi	42516,33	4438519,29
Quercus broteroi	43195,81	4448511,38
Quercus broteroi	43867,70	4458504,00
Quercus broteroi	44549,04	4468496,12
Quercus broteroi	51170,00	4417829,45
Quercus broteroi	51842,97	4427820,77
Quercus broteroi	54552,17	4467785,76
Quercus broteroi	71172,45	4416458,64
Quercus broteroi	71590,02	4115179,56
Quercus broteroi	72856,70	4135154,61
Quercus broteroi	97339,35	4203836,20
Quercus broteroi	147303,48	4200641,94
Quercus broteroi	166641,29	4189380,24
Quercus broteroi	167284,35	4199361,43
Quercus broteroi	93856,61	4304535,12
Quercus broteroi	103847,79	4303806,80
Quercus broteroi	113844,60	4303202,21
Quercus broteroi	159878,25	4239922,89
Quercus broteroi	160526,16	4249904,86
Quercus broteroi	167927,65	4209342,70
Quercus broteroi	169867,73	4239286,63
Quercus broteroi	170520,70	4249267,86
Quercus broteroi	171165,14	4259249,53
Quercus broteroi	171818,53	4269230,92
Quercus broteroi	173125,91	4289193,94
Quercus broteroi	85171,63	4325140,68
Quercus broteroi	86494,88	4345115,94
Quercus broteroi	95830,47	4334495,75
Quercus broteroi	96492,56	4344482,57
Quercus broteroi	104503,97	4313792,89
Quercus broteroi	110483,92	4403778,83

Quercus broteroi	118472,50	4373099,51
Quercus broteroi	139791,77	4391776,01
Quercus broteroi	177083,11	4349194,92
Quercus broteroi	101834,35	4424488,92
Quercus broteroi	122497,27	4433123,07
Quercus broteroi	132483,96	4432411,29
Quercus broteroi	142479,51	4431712,31
Quercus broteroi	143821,22	4451681,06
Quercus broteroi	153137,11	4441010,71
Quercus broteroi	167199,84	4500348,00
Quercus broteroi	100691,42	4555081,32
Quercus broteroi	101380,19	4565069,23
Quercus broteroi	102077,93	4575056,72
Quercus broteroi	107939,93	4514371,72
Quercus broteroi	121370,95	4563670,06
Quercus broteroi	122057,85	4573656,65
Quercus broteroi	131351,29	4562936,27
Quercus broteroi	142038,76	4572311,86
Quercus broteroi	151335,26	4561620,20
Quercus broteroi	154103,58	4601558,44
Quercus broteroi	159263,35	4530975,47
Quercus broteroi	161325,06	4560927,28
Quercus broteroi	164096,58	4600863,21
Quercus broteroi	169252,56	4530297,89
Quercus broteroi	169937,16	4540281,35
Quercus broteroi	170621,93	4550264,88
Quercus broteroi	172000,36	4570231,83
Quercus broteroi	178549,65	4519540,13
Quercus broteroi	179916,72	4539506,10
Quercus broteroi	180609,48	4549600,07
Quercus broteroi	191275,45	4558820,74
Quercus broteroi	191966,59	4568803,30
Quercus broteroi	193348,93	4588768,69
Quercus broteroi	155497,64	4621527,60
Quercus broteroi	186846,39	4639448,58
Quercus broteroi	207712,78	4046362,08
Quercus broteroi	218939,09	4065763,05
Quercus broteroi	228415,15	4075121,50
Quercus broteroi	177270,72	4198739,74
Quercus broteroi	187257,31	4198130,29
Quercus broteroi	217210,92	4196153,39
Quercus broteroi	227186,31	4195482,18
Quercus broteroi	177919,33	4208720,29
Quercus broteroi	179208,31	4228681,99
Quercus broteroi	181805,80	4268605,70
Quercus broteroi	188547,74	4218090,82
Quercus broteroi	189193,14	4228071,21
Quercus broteroi	191138,77	4258012,59
Quercus broteroi	198532,28	4217381,69
Quercus broteroi	201126,23	4257301,67

Quercus broteroi	208512,54	4216796,20
Quercus broteroi	209159,59	4226775,72
Quercus broteroi	221089,22	4256028,91
Quercus broteroi	227835,23	4205460,80
Quercus broteroi	234612,40	4205015,81
Quercus broteroi	184423,11	4308530,07
Quercus broteroi	216354,67	4336554,14
Quercus broteroi	211032,26	4407119,78
Quercus broteroi	221682,22	4416397,08
Quercus broteroi	224377,69	4456319,81
Quercus broteroi	227096,36	4496243,31
Quercus broteroi	231664,84	4415706,54
Quercus broteroi	235033,83	4465608,94
Quercus broteroi	237066,40	4495551,24
Quercus broteroi	239988,01	4415085,41
Quercus broteroi	243527,41	4484988,55
Quercus broteroi	211926,79	4567430,20
Quercus broteroi	222601,56	4576690,25
Quercus broteroi	243936,92	4595251,25
Quercus broteroi	204720,70	4608094,80
Quercus broteroi	225374,96	4616617,63
Quercus broteroi	236049,77	4625890,61
Quercus broteroi	265015,85	3994975,13
Quercus broteroi	275010,81	3994934,69
Quercus broteroi	255009,36	4034892,53
Quercus broteroi	255013,09	4054881,64
Quercus broteroi	255015,55	4064987,49
Quercus broteroi	265014,39	4024954,42
Quercus broteroi	265010,47	4044941,57
Quercus broteroi	265014,80	4054935,24
Quercus broteroi	265011,41	4064929,30
Quercus broteroi	265009,24	4074923,49
Quercus broteroi	275006,72	4004926,84
Quercus broteroi	275006,49	4064882,77
Quercus broteroi	275013,43	4074986,88
Quercus broteroi	285007,51	4004896,81
Quercus broteroi	285012,36	4054966,39
Quercus broteroi	285012,52	4064958,64
Quercus broteroi	285013,80	4074951,03
Quercus broteroi	295013,50	4024970,48
Quercus broteroi	295007,77	4034961,56
Quercus broteroi	295012,07	4044952,57
Quercus broteroi	265012,38	4194977,95
Quercus broteroi	275008,10	4154939,42
Quercus broteroi	295012,16	4194950,79
Quercus broteroi	245006,85	4204907,29
Quercus broteroi	255008,37	4204934,79
Quercus broteroi	275013,99	4204915,22
Quercus broteroi	275008,29	4214911,10
Quercus broteroi	295004,56	4274906,72

Quercus broteroi	245661,71	4354883,88
Quercus broteroi	275011,81	4394975,84
Quercus broteroi	285011,23	4364918,40
Quercus broteroi	285007,14	4384914,09
Quercus broteroi	295012,10	4334880,68
Quercus broteroi	295005,70	4364870,30
Quercus broteroi	295014,62	4374977,98
Quercus broteroi	295013,19	4384975,04
Quercus broteroi	247851,32	4484841,45
Quercus broteroi	255007,95	4414922,65
Quercus broteroi	255012,31	4424923,98
Quercus broteroi	255010,12	4464931,82
Quercus broteroi	255009,33	4484936,87
Quercus broteroi	275014,11	4444973,36
Quercus broteroi	255091,25	4614874,19
Quercus broteroi	255434,32	4634873,18
Quercus broteroi	305008,40	4034952,14
Quercus broteroi	305012,80	4054932,92
Quercus broteroi	315007,39	4044943,94
Quercus broteroi	315007,44	4054933,74
Quercus broteroi	325010,31	4054946,00
Quercus broteroi	325008,62	4064935,32
Quercus broteroi	345002,98	4054894,69
Quercus broteroi	375008,00	4074933,21
Quercus broteroi	385009,00	4074903,44
Quercus broteroi	315010,10	4184927,36
Quercus broteroi	325010,89	4194922,09
Quercus broteroi	335006,10	4194936,95
Quercus broteroi	305010,02	4204922,17
Quercus broteroi	315010,15	4214904,33
Quercus broteroi	325008,03	4214905,55
Quercus broteroi	335005,19	4204927,91
Quercus broteroi	335007,89	4234901,70
Quercus broteroi	345005,09	4204954,21
Quercus broteroi	345004,99	4294874,54
Quercus broteroi	365007,36	4224911,27
Quercus broteroi	365004,60	4264981,95
Quercus broteroi	375007,07	4254941,13
Quercus broteroi	375005,51	4264930,80
Quercus broteroi	385003,83	4214947,96
Quercus broteroi	385008,49	4244913,92
Quercus broteroi	385002,58	4254903,00
Quercus broteroi	385006,04	4264892,11
Quercus broteroi	385001,42	4274881,49
Quercus broteroi	395001,95	4224912,04
Quercus broteroi	395007,27	4244888,61
Quercus broteroi	395006,43	4254877,18
Quercus broteroi	305012,45	4334958,79
Quercus broteroi	305010,79	4344954,33
Quercus broteroi	305010,84	4364945,84

Quercus broteroi	305012,56	4374941,82
Quercus broteroi	305006,81	4384938,16
Quercus broteroi	315006,87	4304955,57
Quercus broteroi	315006,58	4314949,78
Quercus broteroi	315009,16	4334938,65
Quercus broteroi	325006,22	4304950,28
Quercus broteroi	325010,64	4314943,66
Quercus broteroi	325007,40	4324937,38
Quercus broteroi	325009,07	4384902,82
Quercus broteroi	335005,33	4314950,40
Quercus broteroi	335009,08	4334936,41
Quercus broteroi	335007,96	4354923,20
Quercus broteroi	335009,12	4384904,59
Quercus broteroi	345003,66	4334954,50
Quercus broteroi	345011,47	4344946,99
Quercus broteroi	345008,47	4384919,31
Quercus broteroi	355009,92	4324882,20
Quercus broteroi	355011,12	4364961,67
Quercus broteroi	365009,29	4314935,10
Quercus broteroi	365007,31	4324926,27
Quercus broteroi	375008,26	4314881,36
Quercus broteroi	375007,41	4334973,77
Quercus broteroi	375004,57	4364947,11
Quercus broteroi	385008,45	4314951,27
Quercus broteroi	385007,00	4354912,76
Quercus broteroi	395000,88	4344892,46
Quercus broteroi	395002,20	4374974,41
Quercus broteroi	395009,08	4384964,96
Quercus broteroi	305007,40	4454916,44
Quercus broteroi	315011,33	4454886,70
Quercus broteroi	325004,16	4434878,88
Quercus broteroi	325006,03	4454870,42
Quercus broteroi	345010,32	4454877,20
Quercus broteroi	355003,47	4434913,05
Quercus broteroi	355007,01	4444906,69
Quercus broteroi	424999,16	4084886,58
Quercus broteroi	444999,64	4114909,44
Quercus broteroi	494997,69	4194943,33
Quercus broteroi	405001,97	4234887,73
Quercus broteroi	405005,68	4244875,69
Quercus broteroi	405002,50	4254974,86
Quercus broteroi	415003,63	4234887,51
Quercus broteroi	425001,43	4244887,00
Quercus broteroi	425007,07	4254874,43
Quercus broteroi	425005,44	4264973,06
Quercus broteroi	445006,51	4234960,97
Quercus broteroi	455000,60	4244885,85
Quercus broteroi	455004,61	4254983,56
Quercus broteroi	465005,84	4244947,22
Quercus broteroi	485004,22	4294929,81

Quercus broteroi	405007,34	4364966,06
Quercus broteroi	435001,14	4344901,29
Quercus broteroi	445003,94	4354923,99
Quercus broteroi	455000,79	4354970,79
Quercus broteroi	465005,44	4354919,41
Quercus broteroi	514997,42	4204941,16
Quercus canariensis	827999,88	4570198,46
Quercus canariensis	917223,05	4586424,45
Quercus canariensis	926526,37	4597108,94
Quercus canariensis	940925,52	4678490,18
Quercus canariensis	940219,59	4688493,53
Quercus canariensis	955131,18	4619197,63
Quercus canariensis	952331,27	4659204,10
Quercus canariensis	951628,44	4669197,36
Quercus canariensis	950219,26	4689195,01
Quercus canariensis	964433,47	4629900,48
Quercus canariensis	963734,32	4639894,81
Quercus canariensis	963033,43	4649900,25
Quercus canariensis	961628,54	4669899,98
Quercus canariensis	960923,82	4679905,39
Quercus canariensis	974436,26	4630590,86
Quercus canariensis	973736,39	4640597,40
Quercus canariensis	970220,01	4690607,69
Quercus canariensis	2825,21	4139612,41
Quercus canariensis	3462,34	4149618,27
Quercus canariensis	12834,26	4138978,96
Quercus canariensis	13471,71	4148983,65
Quercus canariensis	177272,48	4198717,44
Quercus canariensis	275009,91	3994934,71
Quercus canariensis	255010,71	4014937,30
Quercus canariensis	255010,62	4034936,91
Quercus canariensis	265010,38	4014939,06
Quercus canariensis	265010,17	4044930,48
Quercus canariensis	265010,33	4054935,36
Quercus canariensis	275009,70	4004937,87
Quercus canariensis	275009,77	4024933,58
Quercus canariensis	275009,73	4064938,20
Quercus canariensis	285009,99	4024935,22
Quercus canariensis	285009,51	4044929,98
Quercus canariensis	285009,73	4054933,15
Quercus canariensis	285009,28	4064936,52
Quercus canariensis	295009,11	4054932,83
Quercus canariensis	375006,02	4254930,05
Quercus canariensis	375005,51	4264930,80
Quercus canariensis	385005,51	4254925,16
Quercus canariensis	2495,15	4157680,05
Quercus canariensis	-9383,73	4150282,41
Quercus canariensis	-8760,06	4150613,84
Quercus canariensis	12771,85	4151242,69
Quercus canariensis	9863,19	4145327,09

Quercus x coutinhoi	-56238,57	4314326,23
Quercus x coutinhoi	-46223,30	4313739,95
Quercus x coutinhoi	40493,94	4408431,01
Quercus x coutinhoi	-8773,94	4421808,10
Quercus x coutinhoi	33861,34	4459115,42
Quercus x coutinhoi	34545,89	4469219,89
Quercus x coutinhoi	41846,33	4428526,70
Quercus x coutinhoi	42516,33	4438519,29
Quercus x coutinhoi	52516,83	4437812,11
Quercus x coutinhoi	53867,17	4457794,86
Quercus x coutinhoi	55229,51	4477777,19
Quercus x coutinhoi	81392,36	4566412,06
Quercus x coutinhoi	91386,24	4565733,75
Quercus x coutinhoi	170520,70	4249267,86
Quercus x coutinhoi	100691,42	4555081,32
Quercus x coutinhoi	101380,19	4565069,23
Quercus x coutinhoi	102077,93	4575056,72
Quercus x coutinhoi	111376,69	4564306,99
Quercus x coutinhoi	121370,95	4563670,06
Quercus x coutinhoi	122057,85	4573656,65
Quercus x coutinhoi	131351,29	4562936,27
Quercus x coutinhoi	132739,84	4582907,43
Quercus x coutinhoi	140661,74	4552341,55
Quercus x coutinhoi	151335,26	4561620,20
Quercus x coutinhoi	152718,88	4581589,17
Quercus x coutinhoi	161325,06	4560927,28
Quercus x coutinhoi	182682,19	4579549,02
Quercus x coutinhoi	191966,59	4568803,30
Quercus x coutinhoi	208512,54	4216796,20
Quercus x coutinhoi	213312,51	4587394,07
Quercus x coutinhoi	325006,22	4304950,28
Quercus x coutinhoi	325010,64	4314943,66
Quercus x coutinhoi	355007,92	4724909,92
Quercus x coutinhoi	455002,83	4784950,65
Quercus x coutinhoi	485000,85	4774934,59
Quercus x coutinhoi	465098,11	4803659,99
Quercus x coutinhoi	111376,69	4564306,99
Quercus x coutinhoi	101380,19	4565069,23
Quercus x coutinhoi	54279,95	4142486,71
Quercus x coutinhoi	61566,16	4129404,73
Quercus x coutinhoi	-3732,95	4166785,37
Quercus x coutinhoi	6056,54	4166574,48
Quercus x coutinhoi	-9622,19	4157997,57
Quercus estremadurensis	-47096,63	4304757,36
Quercus estremadurensis	-46223,30	4313739,95
Quercus estremadurensis	-21550,30	4382489,87
Quercus estremadurensis	-20649,68	4392250,32
Quercus estremadurensis	-18230,74	4402243,11
Quercus estremadurensis	-14968,63	4419433,17
Quercus estremadurensis	-5911,41	4160283,37

Quercus estremadurensis	-5266,72	4170278,96
Quercus estremadurensis	-3343,29	4200266,80
Quercus estremadurensis	2818,79	4139579,35
Quercus estremadurensis	4746,17	4169674,23
Quercus estremadurensis	53495,31	4146410,90
Quercus estremadurensis	62223,26	4125793,63
Quercus estremadurensis	8609,01	4229642,43
Quercus estremadurensis	9264,89	4239748,13
Quercus estremadurensis	51888,27	4277160,96
Quercus estremadurensis	-10869,25	4391819,47
Quercus estremadurensis	40493,94	4408431,01
Quercus estremadurensis	48489,08	4377752,80
Quercus estremadurensis	1820,34	4431150,64
Quercus estremadurensis	14533,69	4470484,99
Quercus estremadurensis	23861,66	4459851,29
Quercus estremadurensis	24544,21	4469845,46
Quercus estremadurensis	27267,81	4509823,29
Quercus estremadurensis	34545,89	4469219,89
Quercus estremadurensis	37274,99	4509193,03
Quercus estremadurensis	43195,81	4448511,38
Quercus estremadurensis	44549,04	4468496,12
Quercus estremadurensis	45231,30	4478488,26
Quercus estremadurensis	53867,17	4457794,86
Quercus estremadurensis	87273,42	4505801,50
Quercus estremadurensis	81392,36	4566412,06
Quercus estremadurensis	89322,62	4535767,68
Quercus estremadurensis	91386,24	4565733,75
Quercus estremadurensis	92081,02	4575722,20
Quercus estremadurensis	92768,05	4585711,17
Quercus estremadurensis	170520,70	4249267,86
Quercus estremadurensis	117808,62	4363114,08
Quercus estremadurensis	106579,18	4494397,42
Quercus estremadurensis	132483,96	4432411,29
Quercus estremadurensis	133163,61	4442395,83
Quercus estremadurensis	135869,07	4482335,48
Quercus estremadurensis	101380,19	4565069,23
Quercus estremadurensis	102077,93	4575056,72
Quercus estremadurensis	103458,38	4595032,76
Quercus estremadurensis	111376,69	4564306,99
Quercus estremadurensis	112060,61	4574294,47
Quercus estremadurensis	121370,95	4563670,06
Quercus estremadurensis	122057,85	4573656,65
Quercus estremadurensis	131351,29	4562936,27
Quercus estremadurensis	140661,74	4552341,55
Quercus estremadurensis	152718,88	4581589,17
Quercus estremadurensis	161325,06	4560927,28
Quercus estremadurensis	170621,93	4550264,88
Quercus estremadurensis	181297,49	4559583,09
Quercus estremadurensis	182682,19	4579549,02
Quercus estremadurensis	191966,59	4568803,30

Quercus estremadurensis	125532,67	4623588,72
Quercus estremadurensis	144813,54	4612252,45
Quercus estremadurensis	166879,56	4640800,01
Quercus estremadurensis	195430,89	4618717,13
Quercus estremadurensis	208512,54	4216796,20
Quercus estremadurensis	213312,51	4587394,07
Quercus estremadurensis	295013,19	4384975,04
Quercus estremadurensis	265013,36	4434942,99
Quercus estremadurensis	265007,46	4444944,02
Quercus estremadurensis	325010,64	4314943,66
Quercus faginea	762933,60	4495551,24
Quercus faginea	783565,83	4486968,00
Quercus faginea	782889,91	4496949,45
Quercus faginea	755447,45	4504961,45
Quercus faginea	762250,88	4505532,04
Quercus faginea	772224,39	4506224,46
Quercus faginea	770170,81	4536167,94
Quercus faginea	782205,63	4506930,68
Quercus faginea	781525,70	4516800,98
Quercus faginea	780841,68	4526782,42
Quercus faginea	792190,52	4507539,36
Quercus faginea	790820,59	4527502,68
Quercus faginea	790131,52	4537484,32
Quercus faginea	802085,94	4508269,13
Quercus faginea	751596,46	4675144,69
Quercus faginea	793189,45	4638042,42
Quercus faginea	792487,45	4648024,94
Quercus faginea	757630,67	4715729,35
Quercus faginea	777581,61	4717165,09
Quercus faginea	805959,87	4598751,53
Quercus faginea	837984,47	4570911,25
Quercus faginea	847968,87	4571604,45
Quercus faginea	846584,72	4591573,57
Quercus faginea	802470,56	4648665,72
Quercus faginea	813153,61	4639448,58
Quercus faginea	811751,25	4659415,22
Quercus faginea	822438,58	4650101,07
Quercus faginea	821735,65	4660084,77
Quercus faginea	833120,44	4640800,01
Quercus faginea	860272,45	4682877,22
Quercus faginea	954998,60	4619334,20
Quercus faginea	52854,46	4136421,77
Quercus faginea	91386,24	4565733,75
Quercus faginea	151849,59	4270629,97
Quercus faginea	171165,14	4259249,53
Quercus faginea	178410,63	4369158,55
Quercus faginea	101834,35	4424488,92
Quercus faginea	135869,07	4482335,48
Quercus faginea	101380,19	4565069,23
Quercus faginea	102077,93	4575056,72

Quercus faginea	111376,69	4564306,99
Quercus faginea	112060,61	4574294,47
Quercus faginea	121370,95	4563670,06
Quercus faginea	122057,85	4573656,65
Quercus faginea	131351,29	4562936,27
Quercus faginea	140661,74	4552341,55
Quercus faginea	141345,88	4562326,88
Quercus faginea	150648,07	4551635,62
Quercus faginea	152718,88	4581589,17
Quercus faginea	161325,06	4560927,28
Quercus faginea	162015,53	4570911,25
Quercus faginea	162706,22	4580895,29
Quercus faginea	172693,99	4580215,24
Quercus faginea	179228,91	4529523,26
Quercus faginea	179916,72	4539506,10
Quercus faginea	181297,49	4559583,09
Quercus faginea	182682,19	4579549,02
Quercus faginea	183370,48	4589532,30
Quercus faginea	144813,54	4612252,45
Quercus faginea	154800,49	4611542,98
Quercus faginea	164787,87	4610847,49
Quercus faginea	174775,71	4610165,96
Quercus faginea	186149,43	4629465,22
Quercus faginea	186846,39	4639448,58
Quercus faginea	195430,89	4618717,13
Quercus faginea	196829,94	4638682,76
Quercus faginea	191770,80	4709221,44
Quercus faginea	213312,51	4587394,07
Quercus faginea	204720,70	4608094,80
Quercus faginea	214697,77	4607358,37
Quercus faginea	234654,26	4605927,28
Quercus faginea	246298,49	4635192,48
Quercus faginea	236672,59	4776335,85
Quercus faginea	275012,57	4054889,52
Quercus faginea	275006,49	4064882,77
Quercus faginea	285012,36	4054966,39
Quercus faginea	285012,52	4064958,64
Quercus faginea	285013,80	4074951,03
Quercus faginea	285007,29	4084943,79
Quercus faginea	295005,95	4064935,46
Quercus faginea	295013,42	4074926,90
Quercus faginea	255013,23	4474934,13
Quercus faginea	255015,39	4494939,46
Quercus faginea	275008,19	4564875,44
Quercus faginea	255434,32	4634873,18
Quercus faginea	265009,45	4744946,03
Quercus faginea	265016,18	4754952,55
Quercus faginea	275010,84	4754951,17
Quercus faginea	295010,25	4724869,83
Quercus faginea	325008,62	4064935,32

Quercus faginea	335173,71	4045644,25
Quercus faginea	374343,11	4066731,75
Quercus faginea	375008,00	4074933,21
Quercus faginea	375007,64	4094906,87
Quercus faginea	385009,00	4074903,44
Quercus faginea	385004,48	4094987,21
Quercus faginea	395000,69	4074885,52
Quercus faginea	395008,20	4094968,28
Quercus faginea	335005,40	4164965,11
Quercus faginea	375009,23	4124979,50
Quercus faginea	375007,59	4134967,07
Quercus faginea	375006,61	4144954,79
Quercus faginea	385004,46	4124947,51
Quercus faginea	385008,63	4134934,53
Quercus faginea	385004,55	4144921,83
Quercus faginea	395000,43	4134914,06
Quercus faginea	325008,03	4214905,55
Quercus faginea	375007,17	4224973,19
Quercus faginea	375003,53	4234962,40
Quercus faginea	375005,51	4264930,80
Quercus faginea	385004,75	4224936,46
Quercus faginea	385006,31	4234925,12
Quercus faginea	385008,49	4244913,92
Quercus faginea	325006,22	4304950,28
Quercus faginea	325010,64	4314943,66
Quercus faginea	345003,66	4334954,50
Quercus faginea	375009,16	4384930,05
Quercus faginea	335007,97	4494960,64
Quercus faginea	385001,56	4444946,65
Quercus faginea	305008,92	4574899,43
Quercus faginea	395003,85	4574929,06
Quercus faginea	395006,36	4584922,92
Quercus faginea	305007,44	4674905,06
Quercus faginea	305012,51	4684906,48
Quercus faginea	315013,55	4684969,00
Quercus faginea	355003,86	4624933,22
Quercus faginea	385008,77	4654928,99
Quercus faginea	325010,55	4744937,89
Quercus faginea	345004,36	4784902,73
Quercus faginea	365004,42	4774921,68
Quercus faginea	365007,50	4784920,45
Quercus faginea	375005,30	4734961,92
Quercus faginea	395001,63	4734962,10
Quercus faginea	395002,53	4764952,15
Quercus faginea	405002,64	4094961,26
Quercus faginea	465005,64	4094944,65
Quercus faginea	475003,89	4084925,21
Quercus faginea	405008,12	4144891,77
Quercus faginea	425004,99	4164881,76
Quercus faginea	435006,36	4144936,71

Quercus faginea	435002,73	4174894,16
Quercus faginea	455004,41	4114960,95
Quercus faginea	455005,13	4134930,62
Quercus faginea	465002,49	4104928,97
Quercus faginea	464999,53	4114913,44
Quercus faginea	465003,94	4174934,32
Quercus faginea	425001,43	4244887,00
Quercus faginea	425007,07	4254874,43
Quercus faginea	425005,44	4264973,06
Quercus faginea	435000,76	4254898,42
Quercus faginea	455000,60	4244885,85
Quercus faginea	464999,64	4204891,39
Quercus faginea	475001,36	4204965,64
Quercus faginea	445003,94	4354923,99
Quercus faginea	435003,46	4484869,42
Quercus faginea	445001,95	4434948,86
Quercus faginea	445002,37	4454928,90
Quercus faginea	465003,70	4464910,46
Quercus faginea	464999,34	4474900,52
Quercus faginea	475004,43	4434901,78
Quercus faginea	475002,29	4454880,82
Quercus faginea	485005,00	4454965,30
Quercus faginea	485002,38	4474944,80
Quercus faginea	484996,97	4484934,80
Quercus faginea	415004,69	4544911,26
Quercus faginea	415002,27	4594875,82
Quercus faginea	425004,58	4524929,17
Quercus faginea	435001,13	4504962,58
Quercus faginea	435004,30	4514953,86
Quercus faginea	435000,84	4564912,78
Quercus faginea	435006,38	4574904,99
Quercus faginea	445004,92	4504881,74
Quercus faginea	445000,39	4584925,59
Quercus faginea	445001,27	4594917,90
Quercus faginea	465001,53	4514973,23
Quercus faginea	464998,26	4524964,06
Quercus faginea	475000,21	4534914,24
Quercus faginea	484999,65	4564971,28
Quercus faginea	494996,61	4514892,16
Quercus faginea	405000,20	4604883,31
Quercus faginea	405006,94	4624871,27
Quercus faginea	415005,03	4614862,72
Quercus faginea	415008,54	4624967,44
Quercus faginea	425003,44	4614973,25
Quercus faginea	445000,84	4684966,93
Quercus faginea	454998,29	4644922,51
Quercus faginea	455003,73	4654915,52
Quercus faginea	465002,05	4634873,46
Quercus faginea	465001,45	4644866,18
Quercus faginea	475004,99	4644934,96

Quercus faginea	475000,56	4694900,24
Quercus faginea	494996,93	4624908,00
Quercus faginea	405000,45	4764917,04
Quercus faginea	415006,15	4764896,45
Quercus faginea	425006,54	4704919,27
Quercus faginea	425007,49	4714914,07
Quercus faginea	425005,13	4744899,49
Quercus faginea	424999,16	4774886,40
Quercus faginea	435003,21	4714924,41
Quercus faginea	435006,66	4724919,04
Quercus faginea	435006,64	4744908,83
Quercus faginea	445006,80	4714949,08
Quercus faginea	445001,65	4734938,05
Quercus faginea	464998,67	4734918,75
Quercus faginea	465000,37	4744913,04
Quercus faginea	465002,29	4754907,48
Quercus faginea	475004,91	4734875,27
Quercus faginea	485000,41	4744951,44
Quercus faginea	495003,03	4714955,31
Quercus faginea	495002,03	4724949,02
Quercus faginea	495001,06	4734942,88
Quercus faginea	494998,39	4764925,43
Quercus faginea	494793,04	4794576,38
Quercus faginea	515001,22	4084901,69
Quercus faginea	515001,37	4094885,65
Quercus faginea	524998,69	4074941,34
Quercus faginea	524996,11	4084925,21
Quercus faginea	505002,31	4194943,33
Quercus faginea	544995,35	4124945,71
Quercus faginea	504996,21	4204928,93
Quercus faginea	514997,42	4204941,16
Quercus faginea	525002,97	4234923,73
Quercus faginea	525000,61	4274980,95
Quercus faginea	535002,43	4234960,80
Quercus faginea	554998,52	4264921,91
Quercus faginea	584996,37	4234887,51
Quercus faginea	515001,28	4314904,34
Quercus faginea	504999,95	4494911,50
Quercus faginea	565000,13	4464887,96
Quercus faginea	574993,52	4484963,07
Quercus faginea	574994,68	4494954,37
Quercus faginea	505001,69	4504901,75
Quercus faginea	504996,63	4524882,72
Quercus faginea	534998,47	4514973,23
Quercus faginea	544999,68	4544889,72
Quercus faginea	574995,63	4514937,42
Quercus faginea	574997,72	4594875,99
Quercus faginea	505000,36	4604923,89
Quercus faginea	525000,36	4614957,71
Quercus faginea	525001,19	4654927,72

Quercus faginea	524998,93	4664920,60
Quercus faginea	534997,95	4634873,46
Quercus faginea	534998,43	4674956,29
Quercus faginea	534997,58	4694943,14
Quercus faginea	544998,57	4634929,60
Quercus faginea	544994,79	4684895,61
Quercus faginea	555000,08	4634888,76
Quercus faginea	564992,62	4634972,90
Quercus faginea	564998,10	4674947,41
Quercus faginea	575000,00	4624966,65
Quercus faginea	574994,65	4634960,12
Quercus faginea	584993,76	4644955,37
Quercus faginea	594992,56	4614877,16
Quercus faginea	504996,97	4714955,31
Quercus faginea	504997,97	4724949,02
Quercus faginea	504998,94	4734942,88
Quercus faginea	504999,86	4744936,91
Quercus faginea	515002,26	4754945,67
Quercus faginea	525001,69	4714887,46
Quercus faginea	534994,60	4724924,58
Quercus faginea	535001,33	4734918,75
Quercus faginea	574997,31	4734904,22
Quercus faginea	575000,84	4774886,40
Quercus faginea	604996,95	4174973,20
Quercus faginea	624996,99	4194895,78
Quercus faginea	684989,41	4284967,59
Quercus faginea	684991,80	4294961,51
Quercus faginea	694991,27	4284872,96
Quercus faginea	694987,09	4294978,54
Quercus faginea	634993,89	4334917,59
Quercus faginea	634993,97	4354900,69
Quercus faginea	644990,12	4344977,13
Quercus faginea	654996,34	4334954,50
Quercus faginea	654988,53	4344946,99
Quercus faginea	664990,88	4384904,59
Quercus faginea	604996,76	4444912,30
Quercus faginea	604994,14	4454904,03
Quercus faginea	604999,39	4464896,04
Quercus faginea	614994,02	4424962,60
Quercus faginea	664989,65	4414887,50
Quercus faginea	664992,53	4474969,12
Quercus faginea	674993,97	4454870,42
Quercus faginea	694992,60	4454916,44
Quercus faginea	614991,57	4594958,32
Quercus faginea	624992,34	4584907,45
Quercus faginea	644996,11	4534968,67
Quercus faginea	644992,28	4544963,98
Quercus faginea	654991,42	4534950,38
Quercus faginea	654990,82	4544946,40
Quercus faginea	604998,20	4714969,55

Quercus faginea	624994,70	4734961,92
Quercus faginea	634990,65	4734927,95
Quercus faginea	644992,08	4724909,92
Quercus faginea	644995,47	4734908,60
Quercus faginea	644991,17	4754906,27
Quercus faginea	694990,01	4704910,20
Quercus faginea	704991,28	4284901,87
Quercus faginea	714992,02	4284943,31
Quercus faginea	714991,56	4294939,57
Quercus faginea	724992,35	4294883,35
Quercus faginea	734990,35	4284952,61
Quercus faginea	714989,88	4304935,98
Quercus faginea	724995,46	4304880,78
Quercus faginea	714994,48	4424907,47
Quercus faginea	724989,09	4424973,87
Quercus faginea	724985,86	4464973,68
Quercus faginea	734991,59	4464946,24
Quercus faginea	744986,77	4474934,13
Quercus faginea	744990,67	4484936,87
Quercus faginea	752489,96	4464846,38
Quercus faginea	704991,39	4624957,46
Quercus faginea	704986,93	4634958,86
Quercus faginea	704990,55	4664964,65
Quercus faginea	714988,81	4664928,61
Quercus faginea	714989,26	4684935,20
Quercus lusitanica	-66255,33	4315037,03
Quercus lusitanica	-56906,32	4304323,32
Quercus lusitanica	-56247,26	4314326,84
Quercus lusitanica	-38838,00	4273051,80
Quercus lusitanica	-38182,46	4283052,57
Quercus lusitanica	-37525,55	4293053,32
Quercus lusitanica	-28824,31	4272384,83
Quercus lusitanica	-28172,67	4282384,52
Quercus lusitanica	-27510,99	4292383,62
Quercus lusitanica	-45566,86	4323742,31
Quercus lusitanica	-35555,29	4323056,01
Quercus lusitanica	-32889,95	4363169,60
Quercus lusitanica	-32230,73	4373170,75
Quercus lusitanica	-31561,51	4383171,29
Quercus lusitanica	-25544,18	4322382,55
Quercus lusitanica	-24217,72	4342381,17
Quercus lusitanica	-23549,42	4352491,84
Quercus lusitanica	4748,10	4762787,50
Quercus lusitanica	-29112,01	4111534,42
Quercus lusitanica	-28475,93	4121532,70
Quercus lusitanica	-17829,39	4130907,22
Quercus lusitanica	-17193,92	4140904,40
Quercus lusitanica	-13348,30	4200998,43
Quercus lusitanica	-7179,57	4140291,11
Quercus lusitanica	-6546,08	4150287,24

Quercus lusitanica	-5911,41	4160283,37
Quercus lusitanica	-5266,72	4170278,96
Quercus lusitanica	-3982,67	4190270,68
Quercus lusitanica	-3343,29	4200266,80
Quercus lusitanica	2818,79	4139579,35
Quercus lusitanica	3457,92	4149573,95
Quercus lusitanica	4104,80	4159679,63
Quercus lusitanica	4746,17	4169674,23
Quercus lusitanica	5379,83	4179669,36
Quercus lusitanica	6668,18	4199658,58
Quercus lusitanica	12832,24	4138990,23
Quercus lusitanica	13468,16	4148983,86
Quercus lusitanica	22838,86	4138302,11
Quercus lusitanica	23471,56	4148294,81
Quercus lusitanica	33489,82	4147728,44
Quercus lusitanica	43492,66	4147063,62
Quercus lusitanica	44128,74	4157054,07
Quercus lusitanica	52854,46	4136421,77
Quercus lusitanica	53495,31	4146410,90
Quercus lusitanica	62223,26	4125793,63
Quercus lusitanica	62860,11	4135781,93
Quercus lusitanica	-18154,62	4281728,56
Quercus lusitanica	-8145,76	4281085,82
Quercus lusitanica	-2045,39	4220369,59
Quercus lusitanica	1855,73	4280344,64
Quercus lusitanica	2521,25	4290451,29
Quercus lusitanica	3172,28	4300447,39
Quercus lusitanica	3833,17	4310442,95
Quercus lusitanica	9264,89	4239748,13
Quercus lusitanica	11210,26	4269732,55
Quercus lusitanica	11863,83	4279727,18
Quercus lusitanica	12518,50	4289721,82
Quercus lusitanica	13182,97	4299715,93
Quercus lusitanica	19910,17	4249030,62
Quercus lusitanica	20566,52	4259023,82
Quercus lusitanica	23191,47	4299108,80
Quercus lusitanica	23844,29	4309102,56
Quercus lusitanica	29920,23	4248441,54
Quercus lusitanica	33193,03	4298403,31
Quercus lusitanica	33850,52	4308395,71
Quercus lusitanica	51888,27	4277160,96
Quercus lusitanica	52544,71	4287151,22
Quercus lusitanica	53202,02	4297141,51
Quercus lusitanica	61236,75	4266506,06
Quercus lusitanica	61888,48	4276495,54
Quercus lusitanica	62541,02	4286485,05
Quercus lusitanica	71888,57	4275842,73
Quercus lusitanica	-11539,50	4381821,52
Quercus lusitanica	-863,30	4391163,24
Quercus lusitanica	471,87	4411157,23

Quercus lusitanica	10485,55	4410511,19
Quercus lusitanica	25161,74	4329089,59
Quercus lusitanica	27149,93	4359069,93
Quercus lusitanica	30496,43	4409148,09
Quercus lusitanica	34508,99	4318388,12
Quercus lusitanica	35835,42	4338484,05
Quercus lusitanica	36496,81	4348476,50
Quercus lusitanica	46494,85	4347778,70
Quercus lusitanica	47820,56	4367761,58
Quercus lusitanica	48489,08	4377752,80
Quercus lusitanica	49835,29	4397846,37
Quercus lusitanica	65833,29	4336432,09
Quercus lusitanica	67157,51	4356411,02
Quercus lusitanica	67831,30	4366511,35
Quercus lusitanica	77159,70	4355750,75
Quercus lusitanica	77829,04	4365850,41
Quercus lusitanica	78493,08	4375839,05
Quercus lusitanica	3854,89	4461252,08
Quercus lusitanica	5215,10	4481245,42
Quercus lusitanica	14533,69	4470484,99
Quercus lusitanica	15902,00	4490587,15
Quercus lusitanica	16584,22	4500582,69
Quercus lusitanica	23188,69	4449856,58
Quercus lusitanica	25219,29	4479840,18
Quercus lusitanica	25903,92	4489834,37
Quercus lusitanica	26581,11	4499829,10
Quercus lusitanica	27267,81	4509823,29
Quercus lusitanica	33184,65	4449122,04
Quercus lusitanica	33861,34	4459115,42
Quercus lusitanica	34545,89	4469219,89
Quercus lusitanica	35224,57	4479213,29
Quercus lusitanica	43195,81	4448511,38
Quercus lusitanica	44549,04	4468496,12
Quercus lusitanica	45231,30	4478488,26
Quercus lusitanica	51170,00	4417829,45
Quercus lusitanica	61171,24	4417137,45
Quercus lusitanica	63866,63	4457099,08
Quercus lusitanica	74541,38	4466406,24
Quercus lusitanica	24168,30	4610644,67
Quercus lusitanica	30015,18	4549912,29
Quercus lusitanica	37957,60	4519186,48
Quercus lusitanica	38641,18	4529179,94
Quercus lusitanica	72222,98	4125167,06
Quercus lusitanica	72856,70	4135154,61
Quercus lusitanica	167284,35	4199361,43
Quercus lusitanica	148595,57	4220606,61
Quercus lusitanica	170520,70	4249267,86
Quercus lusitanica	117808,62	4363114,08
Quercus lusitanica	177270,72	4198739,74
Quercus lusitanica	208512,54	4216796,20

Quercus lusitanica	265015,85	3994975,13
Quercus lusitanica	275010,81	3994934,69
Quercus lusitanica	255009,36	4034892,53
Quercus lusitanica	265014,16	4004968,08
Quercus lusitanica	265014,39	4024954,42
Quercus lusitanica	265010,47	4044941,57
Quercus lusitanica	265014,80	4054935,24
Quercus lusitanica	275006,72	4004926,84
Quercus lusitanica	275010,99	4024911,35
Quercus lusitanica	275012,57	4054889,52
Quercus lusitanica	275006,49	4064882,77
Quercus lusitanica	285011,11	4014888,12
Quercus lusitanica	285012,36	4054966,39
Quercus lusitanica	295013,50	4024970,48
Quercus lusitanica	295008,48	4054943,94
Quercus marianica	-56899,63	4304356,34
Quercus marianica	-56240,66	4314371,04
Quercus marianica	-46225,60	4313706,61
Quercus marianica	-22885,89	4362446,66
Quercus marianica	-5908,55	4160272,05
Quercus marianica	-5268,49	4170279,07
Quercus marianica	-4626,57	4180297,20
Quercus marianica	-3984,15	4190304,22
Quercus marianica	-3339,69	4200311,18
Quercus marianica	2825,21	4139612,41
Quercus marianica	3462,34	4149618,27
Quercus marianica	4101,26	4159635,25
Quercus marianica	4741,53	4169641,06
Quercus marianica	5382,92	4179646,88
Quercus marianica	6026,10	4189663,81
Quercus marianica	6669,73	4199669,64
Quercus marianica	7314,47	4209675,46
Quercus marianica	13471,71	4148983,65
Quercus marianica	14109,35	4158988,40
Quercus marianica	14750,48	4169004,15
Quercus marianica	15391,14	4179008,86
Quercus marianica	16033,76	4189013,52
Quercus marianica	25398,36	4178371,97
Quercus marianica	42852,45	4137062,32
Quercus marianica	62224,15	4125793,58
Quercus marianica	-2047,44	4220336,26
Quercus marianica	7961,21	4219681,24
Quercus marianica	8609,73	4229698,13
Quercus marianica	9258,69	4239703,91
Quercus marianica	33192,38	4298436,79
Quercus marianica	39276,76	4237751,22
Quercus marianica	51887,52	4277116,44
Quercus marianica	-10871,83	4391819,64
Quercus marianica	40498,32	4408475,34
Quercus marianica	65176,19	4326464,74

Quercus marianica	13859,50	4460522,61
Quercus marianica	23863,52	4459840,02
Quercus marianica	157296,91	4200006,55
Quercus marianica	170519,43	4249301,27
Quercus marianica	171170,00	4259282,69
Quercus marianica	171822,09	4269275,25
Quercus marianica	177272,48	4198717,44
Quercus marianica	195319,02	4167482,75
Quercus marianica	225905,67	4175546,91
Quercus marianica	181807,99	4268616,73
Quercus marianica	198532,22	4217403,92
Quercus marianica	239985,34	4415107,73
Quercus marianica	275009,91	3994934,71
Quercus marianica	255010,96	4044931,42
Quercus marianica	255011,09	4054937,22
Quercus marianica	255011,28	4074938,25
Quercus marianica	265010,57	4004934,87
Quercus marianica	265010,17	4044930,48
Quercus marianica	265010,33	4054935,36
Quercus marianica	265010,51	4064929,32
Quercus marianica	275009,96	4044929,99
Quercus marianica	285009,51	4044929,98
Quercus marianica	285009,73	4054933,15
Quercus marianica	285009,28	4064936,52
Quercus marianica	285009,67	4074928,93
Quercus marianica	295009,12	4024937,29
Quercus marianica	295009,04	4034939,33
Quercus marianica	295008,86	4044930,45
Quercus marianica	295009,11	4054932,83
Quercus marianica	295009,22	4074938,10
Quercus marianica	265010,76	4414919,72
Quercus marianica	305008,79	4034929,93
Quercus marianica	305008,95	4044931,38
Quercus marianica	305008,33	4054933,02
Quercus marianica	315008,05	4044932,82
Quercus marianica	315008,34	4054933,72
Quercus marianica	325007,40	4054934,96
Quercus marianica	335007,36	4054936,72
Quercus marianica	375006,02	4254930,05
Quercus marianica	375005,51	4264930,80
Quercus marianica	385005,47	4224925,35
Quercus marianica	385005,51	4254925,16
Quercus marianica	305008,56	4374919,70
Quercus marianica	315008,03	4334927,57
Quercus marianica	325007,55	4314921,52
Quercus marianica	365005,64	4384920,98
Quercus marianica	405004,34	4084931,21
Quercus marianica	415004,00	4084936,28
Quercus marianica	445002,43	4264933,00
Quercus marianica	455001,72	4244930,23

Quercus marianica	455001,68	4254928,10
Quercus robur	-61575,38	4309766,61
Quercus robur	-61575,38	4309766,61
Quercus robur	-61575,38	4309766,61
Quercus robur	-61575,38	4309766,61
Quercus robur	-14193,88	4417250,33
Quercus robur	-14193,88	4417250,33
Quercus robur	8523,36	4455933,70
Quercus robur	10561,36	4485953,37
Quercus robur	18529,54	4455256,33
Quercus robur	18529,54	4455256,33
Quercus robur	20567,04	4485272,24
Quercus robur	20567,04	4485272,24
Quercus robur	20567,04	4485272,24
Quercus robur	21248,70	4495277,21
Quercus robur	21931,61	4505282,02
Quercus robur	29891,22	4474587,40
Quercus robur	30571,47	4484591,29
Quercus robur	30571,47	4484591,29
Quercus robur	30571,47	4484591,29
Quercus robur	31252,96	4494595,01
Quercus robur	31935,71	4504598,57
Quercus robur	31935,71	4504598,57
Quercus robur	31935,71	4504598,57
Quercus robur	36512,91	4423892,22
Quercus robur	36512,91	4423892,22
Quercus robur	37186,73	4433895,67
Quercus robur	37186,73	4433895,67
Quercus robur	37861,81	4443898,96
Quercus robur	37861,81	4443898,96
Quercus robur	37861,81	4443898,96
Quercus robur	38538,14	4453902,09
Quercus robur	38538,14	4453902,09
Quercus robur	38538,14	4453902,09
Quercus robur	39215,72	4463905,05
Quercus robur	39215,72	4463905,05
Quercus robur	39215,72	4463905,05
Quercus robur	39215,72	4463905,05
Quercus robur	39215,72	4463905,05
Quercus robur	39215,72	4463905,05
Quercus robur	39215,72	4463905,05
Quercus robur	39215,72	4463905,05
Quercus robur	39894,56	4473907,85
Quercus robur	39894,56	4473907,85
Quercus robur	39894,56	4473907,85
Quercus robur	39894,56	4473907,85
Quercus robur	39894,56	4473907,85
Quercus robur	40574,65	4483910,49
Quercus robur	46515,81	4423219,11
Quercus robur	46515,81	4423219,11

Quercus robur	46515,81	4423219,11
Quercus robur	46515,81	4423219,11
Quercus robur	47189,47	4433221,30
Quercus robur	47189,47	4433221,30
Quercus robur	47864,39	4443223,34
Quercus robur	47864,39	4443223,34
Quercus robur	47864,39	4443223,34
Quercus robur	47864,39	4443223,34
Quercus robur	47864,39	4443223,34
Quercus robur	47864,39	4443223,34
Quercus robur	47864,39	4443223,34
Quercus robur	47864,39	4443223,34
Quercus robur	47864,39	4443223,34
Quercus robur	48540,56	4453225,21
Quercus robur	48540,56	4453225,21
Quercus robur	48540,56	4453225,21
Quercus robur	48540,56	4453225,21
Quercus robur	48540,56	4453225,21
Quercus robur	49896,66	4473228,47
Quercus robur	51257,76	4493231,09
Quercus robur	51940,17	4503232,16
Quercus robur	51940,17	4503232,16
Quercus robur	51940,17	4503232,16
Quercus robur	51940,17	4503232,16
Quercus robur	51940,17	4503232,16
Quercus robur	55845,22	4412545,06
Quercus robur	55845,22	4412545,06
Quercus robur	56517,46	4422546,16
Quercus robur	56517,46	4422546,16
Quercus robur	56517,46	4422546,16
Quercus robur	56517,46	4422546,16
Quercus robur	56517,46	4422546,16
Quercus robur	57190,96	4432547,09
Quercus robur	57865,71	4442547,87
Quercus robur	58541,72	4452548,49
Quercus robur	58541,72	4452548,49
Quercus robur	60577,27	4482549,39
Quercus robur	61258,28	4492549,38
Quercus robur	61258,28	4492549,38
Quercus robur	61258,28	4492549,38
Quercus robur	61940,54	4502549,20
Quercus robur	61940,54	4502549,20
Quercus robur	68541,64	4451871,93
Quercus robur	68541,64	4451871,93
Quercus robur	69218,74	4461871,14
Quercus robur	69218,74	4461871,14
Quercus robur	69218,74	4461871,14
Quercus robur	70576,71	4481869,09
Quercus robur	70576,71	4481869,09
Quercus robur	70576,71	4481869,09

Quercus robur	71257,56	4491867,82
Quercus robur	76516,98	4421200,73
Quercus robur	76516,98	4421200,73
Quercus robur	77864,60	4441197,42
Quercus robur	78540,30	4451195,53
Quercus robur	78540,30	4451195,53
Quercus robur	79217,25	4461193,49
Quercus robur	79217,25	4461193,49
Quercus robur	79217,25	4461193,49
Quercus robur	79217,25	4461193,49
Quercus robur	79217,25	4461193,49
Quercus robur	79217,25	4461193,49
Quercus robur	79217,25	4461193,49
Quercus robur	80574,90	4481188,93
Quercus robur	80574,90	4481188,93
Quercus robur	80574,90	4481188,93
Quercus robur	81255,59	4491186,42
Quercus robur	81255,59	4491186,42
Quercus robur	8820,31	4606713,02
Quercus robur	8820,31	4606713,02
Quercus robur	8820,31	4606713,02
Quercus robur	16050,81	4565998,40
Quercus robur	16050,81	4565998,40
Quercus robur	16742,56	4576003,27
Quercus robur	17435,54	4586007,98
Quercus robur	17435,54	4586007,98
Quercus robur	17435,54	4586007,98
Quercus robur	18129,76	4596012,51
Quercus robur	18129,76	4596012,51
Quercus robur	18129,76	4596012,51
Quercus robur	18129,76	4596012,51
Quercus robur	18129,76	4596012,51
Quercus robur	18825,20	4606016,88
Quercus robur	18825,20	4606016,88
Quercus robur	18825,20	4606016,88
Quercus robur	18825,20	4606016,88
Quercus robur	18825,20	4606016,88
Quercus robur	18825,20	4606016,88
Quercus robur	18825,20	4606016,88
Quercus robur	18825,20	4606016,88
Quercus robur	18825,20	4606016,88
Quercus robur	18825,20	4606016,88
Quercus robur	18825,20	4606016,88
Quercus robur	18825,20	4606016,88
Quercus robur	22615,76	4515286,66
Quercus robur	22615,76	4515286,66
Quercus robur	22615,76	4515286,66
Quercus robur	23301,16	4525291,13
Quercus robur	24675,68	4545299,57
Quercus robur	24675,68	4545299,57

Quercus robur	24675,68	4545299,57
Quercus robur	26055,14	4565307,35
Quercus robur	26055,14	4565307,35
Quercus robur	26055,14	4565307,35
Quercus robur	26055,14	4565307,35
Quercus robur	26746,73	4575310,99
Quercus robur	27439,55	4585314,46
Quercus robur	27439,55	4585314,46
Quercus robur	28133,59	4595317,77
Quercus robur	28133,59	4595317,77
Quercus robur	28133,59	4595317,77
Quercus robur	28828,87	4605320,90
Quercus robur	28828,87	4605320,90
Quercus robur	28828,87	4605320,90
Quercus robur	28828,87	4605320,90
Quercus robur	28828,87	4605320,90
Quercus robur	32619,70	4514601,96
Quercus robur	32619,70	4514601,96
Quercus robur	33304,93	4524605,19
Quercus robur	33304,93	4524605,19
Quercus robur	33304,93	4524605,19
Quercus robur	33304,93	4524605,19
Quercus robur	33304,93	4524605,19
Quercus robur	33304,93	4524605,19
Quercus robur	33304,93	4524605,19
Quercus robur	33304,93	4524605,19
Quercus robur	33304,93	4524605,19
Quercus robur	33991,40	4534608,26
Quercus robur	33991,40	4534608,26
Quercus robur	33991,40	4534608,26
Quercus robur	34679,11	4544611,16
Quercus robur	34679,11	4544611,16
Quercus robur	34679,11	4544611,16
Quercus robur	34679,11	4544611,16
Quercus robur	35368,06	4554613,89
Quercus robur	35368,06	4554613,89
Quercus robur	35368,06	4554613,89
Quercus robur	35368,06	4554613,89
Quercus robur	35368,06	4554613,89
Quercus robur	35368,06	4554613,89
Quercus robur	36058,25	4564616,46
Quercus robur	36058,25	4564616,46
Quercus robur	36058,25	4564616,46
Quercus robur	36058,25	4564616,46
Quercus robur	36058,25	4564616,46
Quercus robur	36058,25	4564616,46
Quercus robur	36749,67	4574618,87
Quercus robur	36749,67	4574618,87
Quercus robur	37442,32	4584621,11
Quercus robur	37442,32	4584621,11

Quercus robur	37442,32	4584621,11
Quercus robur	38136,20	4594623,19
Quercus robur	38136,20	4594623,19
Quercus robur	38136,20	4594623,19
Quercus robur	38831,31	4604625,10
Quercus robur	38831,31	4604625,10
Quercus robur	38831,31	4604625,10
Quercus robur	42622,39	4513917,43
Quercus robur	42622,39	4513917,43
Quercus robur	42622,39	4513917,43
Quercus robur	43307,46	4523919,42
Quercus robur	43307,46	4523919,42
Quercus robur	43307,46	4523919,42
Quercus robur	43307,46	4523919,42
Quercus robur	43307,46	4523919,42
Quercus robur	43307,46	4523919,42
Quercus robur	43993,77	4533921,25
Quercus robur	43993,77	4533921,25
Quercus robur	43993,77	4533921,25
Quercus robur	43993,77	4533921,25
Quercus robur	43993,77	4533921,25
Quercus robur	44681,31	4543922,91
Quercus robur	44681,31	4543922,91
Quercus robur	44681,31	4543922,91
Quercus robur	45370,10	4553924,41
Quercus robur	45370,10	4553924,41
Quercus robur	45370,10	4553924,41
Quercus robur	45370,10	4553924,41
Quercus robur	45370,10	4553924,41
Quercus robur	45370,10	4553924,41
Quercus robur	45370,10	4553924,41
Quercus robur	46060,12	4563925,74
Quercus robur	46751,38	4573926,92
Quercus robur	46751,38	4573926,92
Quercus robur	47443,86	4583927,93
Quercus robur	47443,86	4583927,93
Quercus robur	47443,86	4583927,93
Quercus robur	48137,58	4593928,77
Quercus robur	48137,58	4593928,77
Quercus robur	48832,53	4603929,46
Quercus robur	48832,53	4603929,46
Quercus robur	52623,84	4513233,07
Quercus robur	52623,84	4513233,07
Quercus robur	52623,84	4513233,07
Quercus robur	52623,84	4513233,07
Quercus robur	52623,84	4513233,07
Quercus robur	53308,74	4523233,82
Quercus robur	53308,74	4523233,82
Quercus robur	53308,74	4523233,82
Quercus robur	53308,74	4523233,82

Quercus robur	53308,74	4523233,82
Quercus robur	53308,74	4523233,82
Quercus robur	53308,74	4523233,82
Quercus robur	53308,74	4523233,82
Quercus robur	53308,74	4523233,82
Quercus robur	53308,74	4523233,82
Quercus robur	53308,74	4523233,82
Quercus robur	53308,74	4523233,82
Quercus robur	53994,89	4533234,40
Quercus robur	53994,89	4533234,40
Quercus robur	53994,89	4533234,40
Quercus robur	53994,89	4533234,40
Quercus robur	53994,89	4533234,40
Quercus robur	53994,89	4533234,40
Quercus robur	53994,89	4533234,40
Quercus robur	54682,28	4543234,82
Quercus robur	54682,28	4543234,82
Quercus robur	54682,28	4543234,82
Quercus robur	55370,90	4553235,09
Quercus robur	55370,90	4553235,09
Quercus robur	55370,90	4553235,09
Quercus robur	55370,90	4553235,09
Quercus robur	56060,76	4563235,19
Quercus robur	56060,76	4563235,19
Quercus robur	56751,85	4573235,13
Quercus robur	56751,85	4573235,13
Quercus robur	56751,85	4573235,13
Quercus robur	56751,85	4573235,13
Quercus robur	57444,18	4583234,91
Quercus robur	58137,73	4593234,52
Quercus robur	58137,73	4593234,52
Quercus robur	58137,73	4593234,52
Quercus robur	58137,73	4593234,52
Quercus robur	58137,73	4593234,52
Quercus robur	58137,73	4593234,52
Quercus robur	58832,51	4603233,98
Quercus robur	62624,04	4512548,86
Quercus robur	62624,04	4512548,86
Quercus robur	62624,04	4512548,86
Quercus robur	63308,79	4522548,37
Quercus robur	63308,79	4522548,37
Quercus robur	63994,77	4532547,71
Quercus robur	63994,77	4532547,71
Quercus robur	63994,77	4532547,71
Quercus robur	63994,77	4532547,71
Quercus robur	65370,46	4552545,93
Quercus robur	65370,46	4552545,93
Quercus robur	65370,46	4552545,93
Quercus robur	65370,46	4552545,93
Quercus robur	65370,46	4552545,93

Quercus robur	65370,46	4552545,93
Quercus robur	65370,46	4552545,93
Quercus robur	65370,46	4552545,93
Quercus robur	66060,16	4562544,79
Quercus robur	66060,16	4562544,79
Quercus robur	66060,16	4562544,79
Quercus robur	66751,09	4572543,50
Quercus robur	66751,09	4572543,50
Quercus robur	67443,26	4582542,05
Quercus robur	67443,26	4582542,05
Quercus robur	68136,65	4592540,43
Quercus robur	68136,65	4592540,43
Quercus robur	68136,65	4592540,43
Quercus robur	68136,65	4592540,43
Quercus robur	68831,28	4602538,66
Quercus robur	68831,28	4602538,66
Quercus robur	68831,28	4602538,66
Quercus robur	68831,28	4602538,66
Quercus robur	68831,28	4602538,66
Quercus robur	68831,28	4602538,66
Quercus robur	68831,28	4602538,66
Quercus robur	73307,59	4521863,08
Quercus robur	73307,59	4521863,08
Quercus robur	73307,59	4521863,08
Quercus robur	73307,59	4521863,08
Quercus robur	73993,42	4531861,19
Quercus robur	73993,42	4531861,19
Quercus robur	73993,42	4531861,19
Quercus robur	73993,42	4531861,19
Quercus robur	73993,42	4531861,19
Quercus robur	73993,42	4531861,19
Quercus robur	73993,42	4531861,19
Quercus robur	73993,42	4531861,19
Quercus robur	74680,49	4541859,13
Quercus robur	74680,49	4541859,13
Quercus robur	74680,49	4541859,13
Quercus robur	74680,49	4541859,13
Quercus robur	74680,49	4541859,13
Quercus robur	74680,49	4541859,13
Quercus robur	74680,49	4541859,13
Quercus robur	74680,49	4541859,13
Quercus robur	74680,49	4541859,13
Quercus robur	74680,49	4541859,13
Quercus robur	74680,49	4541859,13
Quercus robur	74680,49	4541859,13
Quercus robur	76058,33	4561854,56
Quercus robur	76058,33	4561854,56
Quercus robur	76058,33	4561854,56
Quercus robur	76749,10	4571852,03
Quercus robur	76749,10	4571852,03

Quercus robur	76749,10	4571852,03
Quercus robur	76749,10	4571852,03
Quercus robur	76749,10	4571852,03
Quercus robur	76749,10	4571852,03
Quercus robur	76749,10	4571852,03
Quercus robur	76749,10	4571852,03
Quercus robur	76749,10	4571852,03
Quercus robur	76749,10	4571852,03
Quercus robur	76749,10	4571852,03
Quercus robur	76749,10	4571852,03
Quercus robur	76749,10	4571852,03
Quercus robur	76749,10	4571852,03
Quercus robur	76749,10	4571852,03
Quercus robur	77441,11	4581849,35
Quercus robur	77441,11	4581849,35
Quercus robur	77441,11	4581849,35
Quercus robur	77441,11	4581849,35
Quercus robur	77441,11	4581849,35
Quercus robur	77441,11	4581849,35
Quercus robur	78134,35	4591846,50
Quercus robur	78134,35	4591846,50
Quercus robur	78134,35	4591846,50
Quercus robur	78134,35	4591846,50
Quercus robur	78828,81	4601843,50
Quercus robur	78828,81	4601843,50
Quercus robur	78828,81	4601843,50
Quercus robur	78828,81	4601843,50
Quercus robur	78828,81	4601843,50
Quercus robur	82620,73	4511180,93
Quercus robur	83305,16	4521177,95
Quercus robur	83305,16	4521177,95
Quercus robur	83305,16	4521177,95
Quercus robur	83990,83	4531174,82
Quercus robur	83990,83	4531174,82
Quercus robur	87437,73	4581156,80
Quercus robur	87437,73	4581156,80
Quercus robur	87437,73	4581156,80
Quercus robur	87437,73	4581156,80
Quercus robur	88130,81	4591152,73
Quercus robur	88130,81	4591152,73
Quercus robur	88130,81	4591152,73
Quercus robur	88130,81	4591152,73
Quercus robur	88130,81	4591152,73
Quercus robur	88130,81	4591152,73
Quercus robur	88825,12	4601148,50
Quercus robur	88825,12	4601148,50
Quercus robur	88825,12	4601148,50
Quercus robur	88825,12	4601148,50
Quercus robur	88825,12	4601148,50
Quercus robur	9517,15	4616718,44

Quercus robur	9517,15	4616718,44
Quercus robur	9517,15	4616718,44
Quercus robur	9517,15	4616718,44
Quercus robur	9517,15	4616718,44
Quercus robur	10215,21	4626723,70
Quercus robur	10215,21	4626723,70
Quercus robur	10215,21	4626723,70
Quercus robur	10215,21	4626723,70
Quercus robur	10215,21	4626723,70
Quercus robur	10215,21	4626723,70
Quercus robur	10914,50	4636728,78
Quercus robur	10914,50	4636728,78
Quercus robur	10914,50	4636728,78
Quercus robur	10914,50	4636728,78
Quercus robur	10914,50	4636728,78
Quercus robur	10914,50	4636728,78
Quercus robur	10914,50	4636728,78
Quercus robur	10914,50	4636728,78
Quercus robur	10914,50	4636728,78
Quercus robur	10914,50	4636728,78
Quercus robur	10914,50	4636728,78
Quercus robur	10914,50	4636728,78
Quercus robur	11615,01	4646733,69
Quercus robur	11615,01	4646733,69
Quercus robur	11615,01	4646733,69
Quercus robur	11615,01	4646733,69
Quercus robur	11615,01	4646733,69
Quercus robur	12316,75	4656738,43
Quercus robur	19521,87	4616021,08
Quercus robur	19521,87	4616021,08
Quercus robur	19521,87	4616021,08
Quercus robur	19521,87	4616021,08
Quercus robur	19521,87	4616021,08
Quercus robur	19521,87	4616021,08
Quercus robur	19521,87	4616021,08
Quercus robur	19521,87	4616021,08
Quercus robur	19521,87	4616021,08
Quercus robur	19521,87	4616021,08
Quercus robur	20219,77	4626025,10
Quercus robur	20219,77	4626025,10
Quercus robur	20219,77	4626025,10
Quercus robur	20219,77	4626025,10
Quercus robur	20219,77	4626025,10
Quercus robur	20219,77	4626025,10
Quercus robur	20219,77	4626025,10
Quercus robur	20219,77	4626025,10
Quercus robur	20219,77	4626025,10
Quercus robur	20219,77	4626025,10
Quercus robur	20219,77	4626025,10
Quercus robur	20219,77	4626025,10

Quercus robur	20219,77	4626025,10
Quercus robur	20219,77	4626025,10
Quercus robur	20918,89	4636028,96
Quercus robur	20918,89	4636028,96
Quercus robur	20918,89	4636028,96
Quercus robur	20918,89	4636028,96
Quercus robur	20918,89	4636028,96
Quercus robur	20918,89	4636028,96
Quercus robur	20918,89	4636028,96
Quercus robur	20918,89	4636028,96
Quercus robur	21619,23	4646032,66
Quercus robur	21619,23	4646032,66
Quercus robur	21619,23	4646032,66
Quercus robur	21619,23	4646032,66
Quercus robur	21619,23	4646032,66
Quercus robur	21619,23	4646032,66
Quercus robur	21619,23	4646032,66
Quercus robur	21619,23	4646032,66
Quercus robur	21619,23	4646032,66
Quercus robur	21619,23	4646032,66
Quercus robur	22320,79	4656036,18
Quercus robur	22320,79	4656036,18
Quercus robur	22320,79	4656036,18
Quercus robur	22320,79	4656036,18
Quercus robur	22320,79	4656036,18
Quercus robur	22320,79	4656036,18
Quercus robur	23023,57	4666039,53
Quercus robur	23023,57	4666039,53
Quercus robur	29525,37	4615323,88
Quercus robur	29525,37	4615323,88
Quercus robur	29525,37	4615323,88
Quercus robur	29525,37	4615323,88
Quercus robur	29525,37	4615323,88
Quercus robur	29525,37	4615323,88
Quercus robur	29525,37	4615323,88
Quercus robur	29525,37	4615323,88
Quercus robur	29525,37	4615323,88
Quercus robur	29525,37	4615323,88
Quercus robur	29525,37	4615323,88
Quercus robur	29525,37	4615323,88
Quercus robur	29525,37	4615323,88
Quercus robur	29525,37	4615323,88
Quercus robur	29525,37	4615323,88
Quercus robur	29525,37	4615323,88
Quercus robur	29525,37	4615323,88
Quercus robur	29525,37	4615323,88
Quercus robur	29525,37	4615323,88
Quercus robur	29525,37	4615323,88
Quercus robur	30223,10	4625326,68
Quercus robur	30223,10	4625326,68

Quercus robur	30223,10	4625326,68
Quercus robur	30223,10	4625326,68
Quercus robur	30223,10	4625326,68
Quercus robur	30223,10	4625326,68
Quercus robur	30223,10	4625326,68
Quercus robur	30223,10	4625326,68
Quercus robur	30223,10	4625326,68
Quercus robur	30223,10	4625326,68
Quercus robur	30223,10	4625326,68
Quercus robur	30223,10	4625326,68
Quercus robur	30223,10	4625326,68
Quercus robur	30223,10	4625326,68
Quercus robur	30223,10	4625326,68
Quercus robur	30223,10	4625326,68
Quercus robur	30922,05	4635329,32
Quercus robur	30922,05	4635329,32
Quercus robur	30922,05	4635329,32
Quercus robur	30922,05	4635329,32
Quercus robur	30922,05	4635329,32
Quercus robur	30922,05	4635329,32
Quercus robur	30922,05	4635329,32
Quercus robur	30922,05	4635329,32
Quercus robur	30922,05	4635329,32
Quercus robur	30922,05	4635329,32
Quercus robur	30922,05	4635329,32
Quercus robur	30922,05	4635329,32
Quercus robur	31622,23	4645331,79
Quercus robur	31622,23	4645331,79
Quercus robur	31622,23	4645331,79
Quercus robur	31622,23	4645331,79
Quercus robur	31622,23	4645331,79
Quercus robur	31622,23	4645331,79
Quercus robur	31622,23	4645331,79
Quercus robur	31622,23	4645331,79
Quercus robur	31622,23	4645331,79
Quercus robur	31622,23	4645331,79
Quercus robur	31622,23	4645331,79
Quercus robur	31622,23	4645331,79
Quercus robur	31622,23	4645331,79
Quercus robur	31622,23	4645331,79
Quercus robur	32323,62	4655334,09
Quercus robur	32323,62	4655334,09
Quercus robur	32323,62	4655334,09
Quercus robur	32323,62	4655334,09
Quercus robur	33026,23	4665336,23
Quercus robur	33026,23	4665336,23
Quercus robur	33026,23	4665336,23
Quercus robur	33026,23	4665336,23
Quercus robur	33026,23	4665336,23
Quercus robur	33026,23	4665336,23

Quercus robur	39527,65	4614626,84
Quercus robur	39527,65	4614626,84
Quercus robur	39527,65	4614626,84
Quercus robur	39527,65	4614626,84
Quercus robur	39527,65	4614626,84
Quercus robur	39527,65	4614626,84
Quercus robur	39527,65	4614626,84
Quercus robur	39527,65	4614626,84
Quercus robur	39527,65	4614626,84
Quercus robur	39527,65	4614626,84
Quercus robur	39527,65	4614626,84
Quercus robur	39527,65	4614626,84
Quercus robur	40225,21	4624628,42
Quercus robur	40225,21	4624628,42
Quercus robur	40225,21	4624628,42
Quercus robur	40225,21	4624628,42
Quercus robur	40225,21	4624628,42
Quercus robur	40225,21	4624628,42
Quercus robur	40225,21	4624628,42
Quercus robur	40225,21	4624628,42
Quercus robur	40225,21	4624628,42
Quercus robur	40225,21	4624628,42
Quercus robur	40924,00	4634629,84
Quercus robur	40924,00	4634629,84
Quercus robur	40924,00	4634629,84
Quercus robur	40924,00	4634629,84
Quercus robur	41624,00	4644631,09
Quercus robur	41624,00	4644631,09
Quercus robur	41624,00	4644631,09
Quercus robur	41624,00	4644631,09
Quercus robur	41624,00	4644631,09
Quercus robur	41624,00	4644631,09
Quercus robur	41624,00	4644631,09
Quercus robur	41624,00	4644631,09
Quercus robur	41624,00	4644631,09
Quercus robur	41624,00	4644631,09
Quercus robur	41624,00	4644631,09
Quercus robur	42325,23	4654632,17
Quercus robur	42325,23	4654632,17
Quercus robur	42325,23	4654632,17
Quercus robur	42325,23	4654632,17
Quercus robur	42325,23	4654632,17
Quercus robur	42325,23	4654632,17
Quercus robur	42325,23	4654632,17
Quercus robur	42325,23	4654632,17
Quercus robur	42325,23	4654632,17
Quercus robur	42325,23	4654632,17
Quercus robur	42325,23	4654632,17
Quercus robur	42325,23	4654632,17
Quercus robur	42325,23	4654632,17

Quercus robur	42325,23	4654632,17
Quercus robur	42325,23	4654632,17
Quercus robur	42325,23	4654632,17
Quercus robur	42325,23	4654632,17
Quercus robur	42325,23	4654632,17
Quercus robur	42325,23	4654632,17
Quercus robur	42325,23	4654632,17
Quercus robur	42325,23	4654632,17
Quercus robur	42325,23	4654632,17
Quercus robur	42325,23	4654632,17
Quercus robur	42325,23	4654632,17
Quercus robur	42325,23	4654632,17
Quercus robur	42325,23	4654632,17
Quercus robur	42325,23	4654632,17
Quercus robur	42325,23	4654632,17
Quercus robur	42325,23	4654632,17
Quercus robur	43027,68	4664633,09
Quercus robur	43027,68	4664633,09
Quercus robur	43027,68	4664633,09
Quercus robur	43027,68	4664633,09
Quercus robur	43027,68	4664633,09
Quercus robur	43027,68	4664633,09
Quercus robur	43027,68	4664633,09
Quercus robur	49528,70	4613929,98
Quercus robur	49528,70	4613929,98
Quercus robur	49528,70	4613929,98
Quercus robur	50226,10	4623930,33
Quercus robur	50226,10	4623930,33
Quercus robur	50226,10	4623930,33
Quercus robur	50226,10	4623930,33
Quercus robur	50226,10	4623930,33
Quercus robur	50924,72	4633930,52
Quercus robur	50924,72	4633930,52
Quercus robur	50924,72	4633930,52
Quercus robur	50924,72	4633930,52
Quercus robur	50924,72	4633930,52
Quercus robur	50924,72	4633930,52
Quercus robur	50924,72	4633930,52
Quercus robur	51624,56	4643930,55
Quercus robur	51624,56	4643930,55
Quercus robur	51624,56	4643930,55
Quercus robur	51624,56	4643930,55
Quercus robur	51624,56	4643930,55
Quercus robur	51624,56	4643930,55
Quercus robur	51624,56	4643930,55
Quercus robur	51624,56	4643930,55
Quercus robur	51624,56	4643930,55
Quercus robur	51624,56	4643930,55
Quercus robur	51624,56	4643930,55
Quercus robur	52325,62	4653930,42

Quercus robur	52325,62	4653930,42
Quercus robur	52325,62	4653930,42
Quercus robur	52325,62	4653930,42
Quercus robur	52325,62	4653930,42
Quercus robur	52325,62	4653930,42
Quercus robur	52325,62	4653930,42
Quercus robur	52325,62	4653930,42
Quercus robur	52325,62	4653930,42
Quercus robur	52325,62	4653930,42
Quercus robur	52325,62	4653930,42
Quercus robur	52325,62	4653930,42
Quercus robur	52325,62	4653930,42
Quercus robur	52325,62	4653930,42
Quercus robur	52325,62	4653930,42
Quercus robur	52325,62	4653930,42
Quercus robur	52325,62	4653930,42
Quercus robur	52325,62	4653930,42
Quercus robur	52325,62	4653930,42
Quercus robur	52325,62	4653930,42
Quercus robur	52325,62	4653930,42
Quercus robur	52325,62	4653930,42
Quercus robur	52325,62	4653930,42
Quercus robur	52325,62	4653930,42
Quercus robur	52325,62	4653930,42
Quercus robur	52325,62	4653930,42
Quercus robur	52325,62	4653930,42
Quercus robur	52325,62	4653930,42
Quercus robur	52325,62	4653930,42
Quercus robur	52325,62	4653930,42
Quercus robur	52325,62	4653930,42
Quercus robur	52325,62	4653930,42
Quercus robur	52325,62	4653930,42
Quercus robur	52325,62	4653930,42
Quercus robur	52325,62	4653930,42
Quercus robur	52325,62	4653930,42
Quercus robur	52325,62	4653930,42
Quercus robur	52325,62	4653930,42
Quercus robur	52325,62	4653930,42
Quercus robur	52325,62	4653930,42
Quercus robur	52325,62	4653930,42
Quercus robur	52325,62	4653930,42
Quercus robur	52325,62	4653930,42
Quercus robur	52325,62	4653930,42
Quercus robur	52325,62	4653930,42
Quercus robur	52325,62	4653930,42
Quercus robur	52325,62	4653930,42
Quercus robur	52325,62	4653930,42
Quercus robur	52325,62	4653930,42
Quercus robur	52325,62	4653930,42
Quercus robur	53027,90	4663930,12

Quercus robur	53027,90	4663930,12
Quercus robur	53027,90	4663930,12
Quercus robur	53027,90	4663930,12
Quercus robur	53027,90	4663930,12
Quercus robur	53027,90	4663930,12
Quercus robur	53027,90	4663930,12
Quercus robur	53027,90	4663930,12
Quercus robur	53027,90	4663930,12
Quercus robur	53027,90	4663930,12
Quercus robur	53731,40	4673929,66
Quercus robur	53731,40	4673929,66
Quercus robur	53731,40	4673929,66
Quercus robur	53731,40	4673929,66
Quercus robur	59528,53	4613233,27
Quercus robur	59528,53	4613233,27
Quercus robur	59528,53	4613233,27
Quercus robur	59528,53	4613233,27
Quercus robur	59528,53	4613233,27
Quercus robur	59528,53	4613233,27
Quercus robur	59528,53	4613233,27
Quercus robur	59528,53	4613233,27
Quercus robur	59528,53	4613233,27
Quercus robur	60225,76	4623232,40
Quercus robur	60225,76	4623232,40
Quercus robur	60225,76	4623232,40
Quercus robur	60225,76	4623232,40
Quercus robur	60225,76	4623232,40
Quercus robur	60225,76	4623232,40
Quercus robur	60225,76	4623232,40
Quercus robur	60924,22	4633231,37
Quercus robur	60924,22	4633231,37
Quercus robur	60924,22	4633231,37
Quercus robur	60924,22	4633231,37
Quercus robur	60924,22	4633231,37
Quercus robur	60924,22	4633231,37
Quercus robur	60924,22	4633231,37
Quercus robur	60924,22	4633231,37
Quercus robur	60924,22	4633231,37
Quercus robur	60924,22	4633231,37
Quercus robur	60924,22	4633231,37
Quercus robur	60924,22	4633231,37
Quercus robur	61623,90	4643230,18
Quercus robur	61623,90	4643230,18
Quercus robur	61623,90	4643230,18
Quercus robur	62324,80	4653228,83
Quercus robur	62324,80	4653228,83
Quercus robur	62324,80	4653228,83
Quercus robur	62324,80	4653228,83
Quercus robur	62324,80	4653228,83
Quercus robur	62324,80	4653228,83

Quercus robur	63026,92	4663227,31
Quercus robur	63026,92	4663227,31
Quercus robur	63026,92	4663227,31
Quercus robur	63026,92	4663227,31
Quercus robur	63026,92	4663227,31
Quercus robur	63026,92	4663227,31
Quercus robur	63026,92	4663227,31
Quercus robur	63026,92	4663227,31
Quercus robur	63730,25	4673225,64
Quercus robur	63730,25	4673225,64
Quercus robur	63730,25	4673225,64
Quercus robur	63730,25	4673225,64
Quercus robur	63730,25	4673225,64
Quercus robur	63730,25	4673225,64
Quercus robur	69527,13	4612536,73
Quercus robur	69527,13	4612536,73
Quercus robur	70224,20	4622534,64
Quercus robur	70224,20	4622534,64
Quercus robur	70224,20	4622534,64
Quercus robur	70224,20	4622534,64
Quercus robur	70224,20	4622534,64
Quercus robur	70224,20	4622534,64
Quercus robur	70224,20	4622534,64
Quercus robur	70224,20	4622534,64
Quercus robur	70224,20	4622534,64
Quercus robur	70224,20	4622534,64
Quercus robur	70224,20	4622534,64
Quercus robur	70224,20	4622534,64
Quercus robur	70224,20	4622534,64
Quercus robur	70922,50	4632532,38
Quercus robur	70922,50	4632532,38
Quercus robur	70922,50	4632532,38
Quercus robur	70922,50	4632532,38
Quercus robur	70922,50	4632532,38
Quercus robur	70922,50	4632532,38
Quercus robur	70922,50	4632532,38
Quercus robur	70922,50	4632532,38
Quercus robur	70922,50	4632532,38
Quercus robur	70922,50	4632532,38
Quercus robur	70922,50	4632532,38
Quercus robur	70922,50	4632532,38
Quercus robur	70922,50	4632532,38
Quercus robur	70922,50	4632532,38
Quercus robur	70922,50	4632532,38
Quercus robur	70922,50	4632532,38
Quercus robur	70922,50	4632532,38
Quercus robur	70922,50	4632532,38
Quercus robur	70922,50	4632532,38
Quercus robur	70922,50	4632532,38
Quercus robur	70922,50	4632532,38

Quercus robur	70922,50	4632532,38
Quercus robur	70922,50	4632532,38
Quercus robur	70922,50	4632532,38
Quercus robur	70922,50	4632532,38
Quercus robur	70922,50	4632532,38
Quercus robur	70922,50	4632532,38
Quercus robur	70922,50	4632532,38
Quercus robur	70922,50	4632532,38
Quercus robur	70922,50	4632532,38
Quercus robur	70922,50	4632532,38
Quercus robur	70922,50	4632532,38
Quercus robur	70922,50	4632532,38
Quercus robur	70922,50	4632532,38
Quercus robur	70922,50	4632532,38
Quercus robur	70922,50	4632532,38
Quercus robur	70922,50	4632532,38
Quercus robur	70922,50	4632532,38
Quercus robur	70922,50	4632532,38
Quercus robur	73024,71	4662524,67
Quercus robur	79524,50	4611840,35
Quercus robur	79524,50	4611840,35
Quercus robur	79524,50	4611840,35
Quercus robur	79524,50	4611840,35
Quercus robur	79524,50	4611840,35
Quercus robur	79524,50	4611840,35
Quercus robur	79524,50	4611840,35
Quercus robur	80221,42	4621837,03
Quercus robur	80221,42	4621837,03
Quercus robur	80221,42	4621837,03
Quercus robur	80221,42	4621837,03
Quercus robur	80221,42	4621837,03
Quercus robur	80221,42	4621837,03
Quercus robur	80221,42	4621837,03
Quercus robur	80221,42	4621837,03
Quercus robur	80221,42	4621837,03
Quercus robur	80221,42	4621837,03
Quercus robur	80221,42	4621837,03
Quercus robur	80919,56	4631833,55
Quercus robur	80919,56	4631833,55
Quercus robur	81618,92	4641829,92
Quercus robur	81618,92	4641829,92
Quercus robur	81618,92	4641829,92
Quercus robur	81618,92	4641829,92
Quercus robur	89520,65	4611144,12
Quercus robur	89520,65	4611144,12
Quercus robur	89520,65	4611144,12
Quercus robur	90217,41	4621139,58
Quercus robur	90217,41	4621139,58
Quercus robur	90915,39	4631134,88
Quercus robur	90915,39	4631134,88

Quercus robur	90915,39	4631134,88
Quercus robur	90915,39	4631134,88
Quercus robur	90915,39	4631134,88
Quercus robur	90915,39	4631134,88
Quercus robur	90915,39	4631134,88
Quercus robur	90915,39	4631134,88
Quercus robur	91614,59	4641130,03
Quercus robur	91614,59	4641130,03
Quercus robur	92315,02	4651125,02
Quercus robur	112491,92	4358548,25
Quercus robur	112491,92	4358548,25
Quercus robur	112491,92	4358548,25
Quercus robur	113156,93	4368542,53
Quercus robur	89214,50	4460515,99
Quercus robur	89892,55	4470512,54
Quercus robur	89892,55	4470512,54
Quercus robur	89892,55	4470512,54
Quercus robur	89892,55	4470512,54
Quercus robur	89892,55	4470512,54
Quercus robur	89892,55	4470512,54
Quercus robur	89892,55	4470512,54
Quercus robur	90571,84	4480508,94
Quercus robur	90571,84	4480508,94
Quercus robur	90571,84	4480508,94
Quercus robur	90571,84	4480508,94
Quercus robur	90571,84	4480508,94
Quercus robur	90571,84	4480508,94
Quercus robur	91252,38	4490505,18
Quercus robur	91252,38	4490505,18
Quercus robur	91252,38	4490505,18
Quercus robur	91252,38	4490505,18
Quercus robur	91252,38	4490505,18
Quercus robur	91252,38	4490505,18
Quercus robur	91252,38	4490505,18
Quercus robur	91934,17	4500501,27
Quercus robur	100567,54	4479829,09
Quercus robur	100567,54	4479829,09
Quercus robur	101247,92	4489824,09
Quercus robur	101929,56	4499818,93
Quercus robur	101929,56	4499818,93
Quercus robur	107853,52	4439172,91
Quercus robur	108528,76	4449167,26
Quercus robur	109882,99	4469155,51
Quercus robur	110561,98	4479149,40
Quercus robur	110561,98	4479149,40
Quercus robur	93986,99	4530488,60
Quercus robur	93986,99	4530488,60
Quercus robur	96050,97	4560474,56
Quercus robur	96050,97	4560474,56
Quercus robur	96050,97	4560474,56

Quercus robur	96050,97	4560474,56
Quercus robur	96050,97	4560474,56
Quercus robur	97433,12	4580464,42
Quercus robur	98820,20	4600453,66
Quercus robur	98820,20	4600453,66
Quercus robur	98820,20	4600453,66
Quercus robur	98820,20	4600453,66
Quercus robur	98820,20	4600453,66
Quercus robur	98820,20	4600453,66
Quercus robur	102612,44	4509813,62
Quercus robur	105356,36	4549790,86
Quercus robur	106045,44	4559784,79
Quercus robur	108814,05	4599758,97
Quercus robur	108814,05	4599758,97
Quercus robur	108814,05	4599758,97
Quercus robur	116038,67	4559095,17
Quercus robur	122599,18	4508446,92
Quercus robur	127411,92	4578388,18
Quercus robur	127411,92	4578388,18
Quercus robur	133274,36	4517754,61
Quercus robur	99515,58	4610448,05
Quercus robur	99515,58	4610448,05
Quercus robur	99515,58	4610448,05
Quercus robur	100212,18	4620442,29
Quercus robur	101609,05	4640430,30
Quercus robur	101609,05	4640430,30
Quercus robur	101609,05	4640430,30
Quercus robur	101609,05	4640430,30
Quercus robur	120198,05	4619048,16
Quercus robur	131585,11	4638332,01
Quercus robur	141574,70	4637632,88
Quercus robur	141574,70	4637632,88
Quercus robur	152262,57	4646921,58
Quercus robur	152262,57	4646921,58
Quercus robur	182219,91	4644821,85
Quercus robur	182219,91	4644821,85
Quercus robur	43880,31	4483498,05
Quercus robur	89553,22	4642890,30
Quercus robur	39830,70	4570701,69
Quercus robur	43684,89	4586997,33
Quercus robur	51494,46	4614408,97
Quercus robur	81411,09	4463536,71
Quercus robur	72186,91	4636756,44
Quercus robur	59609,92	4600048,35
Quercus robur	39321,09	4658660,14
Quercus robur	71362,88	4631176,41
Quercus robur	68096,91	4661853,46
Quercus robur	68227,46	4662179,96
Quercus robur	73086,90	4666779,07
Quercus robur	72748,94	4665450,57

Quercus robur	67788,14	4636653,80
Quercus robur	42780,33	4652535,34
Quercus robur	72824,62	4638624,41
Quercus robur	68227,46	4662179,96
Quercus robur	69114,58	4636919,13
Quercus robur	67669,79	4635334,31
Quercus robur	81131,29	4463496,56
Quercus robur	75321,23	4629680,44
Quercus robur	39537,87	4570842,87
Quercus robur	46518,48	4522972,00
Quercus robur	73763,11	4640741,93
Quercus robur	115823,53	4483370,92
Quercus robur	80133,96	4486792,49
Quercus robur	58516,08	4459371,61
Quercus robur	96146,76	4594368,23
Quercus robur	68662,04	4664082,23
Quercus robur	96815,01	4593640,16
Quercus robur	44162,47	4652981,83
Quercus robur	39789,05	4653743,78
Quercus robur	44352,04	4652936,19
Quercus robur	57375,42	4649926,39
Quercus robur	65069,95	4657612,47
Quercus robur	64368,26	4653953,88
Quercus robur	63302,15	4653071,70
Quercus robur	66658,76	4618472,48
Quercus robur	33136,80	4544772,02
Quercus robur	30672,48	4546256,38
Quercus robur	30375,87	4532121,55
Quercus robur	21212,94	4646882,76
Quercus robur	44018,55	4567115,19
Quercus robur	37344,63	4643327,41
Quercus robur	42131,71	4635711,98
Quercus robur	47183,12	4534693,98
Quercus robur	52350,67	4534233,23
Quercus robur	20043,89	4590879,19
Quercus robur	80001,28	4626127,09
Quercus robur	75523,73	4468244,88
Quercus robur	81109,88	4626964,87
Quercus robur	37233,99	4624560,76
Quercus robur	38735,75	4626348,95
Quercus robur	34605,44	4616264,36
Quercus robur	35380,59	4633225,70
Quercus robur	42357,61	4633410,95
Quercus robur	42412,67	4633318,19
Quercus robur	42623,74	4633103,90
Quercus robur	37598,58	4624816,07
Quercus robur	38743,13	4626359,63
Quercus robur	37317,44	4614862,09
Quercus robur	37222,19	4614801,30
Quercus robur	31722,25	4612725,86

Quercus robur	39022,97	4628092,84
Quercus robur	39209,54	4628158,89
Quercus robur	33881,55	4617025,25
Quercus robur	34586,06	4638687,93
Quercus robur	31935,90	4638091,66
Quercus robur	38666,78	4614384,91
Quercus robur	38647,09	4615278,50
Quercus robur	36272,01	4623027,85
Quercus robur	22518,82	4610687,03
Quercus robur	37891,20	4614312,06
Quercus robur	37043,66	4615359,38
Quercus robur	23426,83	4609466,44
Quercus robur	34464,12	4624316,40
Quercus robur	34467,11	4509781,12
Quercus robur	28563,79	4504186,77
Quercus robur	20992,31	4608646,20
Quercus robur	25036,90	4624277,19
Quercus robur	35749,37	4622738,20
Quercus robur	21724,70	4631705,92
Quercus robur	35493,25	4633865,38
Quercus robur	35100,40	4636891,79
Quercus robur	38454,99	4634342,59
Quercus robur	31377,34	4622688,15
Quercus robur	32150,09	4631607,02
Quercus robur	38786,39	4647528,53
Quercus robur	37720,90	4647664,67
Quercus robur	40803,32	4621319,44
Quercus robur	36557,48	4618547,49
Quercus robur	38679,33	4649643,79
Quercus robur	39174,48	4651786,90
Quercus robur	41841,88	4649305,28
Quercus robur	21538,18	4605976,67
Quercus robur	21370,19	4653054,36
Quercus robur	92314,63	4637505,30
Quercus robur	96178,34	4640695,03
Quercus robur	105061,34	4646948,34
Quercus robur	27144,53	4651640,78
Quercus robur	29559,27	4652908,52
Quercus robur	26102,79	4648262,40
Quercus robur	31603,41	4643223,16
Quercus robur	53914,30	4637084,43
Quercus robur	56650,82	4636958,38
Quercus robur	18076,62	4609197,92
Quercus robur	21473,89	4619459,70
Quercus robur	19415,11	4625723,49
Quercus robur	20169,57	4621789,69
Quercus robur	76278,00	4628319,53
Quercus robur	82537,86	4631172,25
Quercus robur	89540,49	4637040,43
Quercus robur	84912,54	4626888,08

Quercus robur	44415,83	4629007,62
Quercus robur	42536,28	4591430,34
Quercus robur	74527,43	4532328,67
Quercus robur	77939,36	4538116,46
Quercus robur	84660,50	4595988,83
Quercus robur	22180,03	4652486,47
Quercus robur	34893,69	4605102,06
Quercus robur	35812,62	4601428,80
Quercus robur	28258,33	4599966,62
Quercus robur	48117,16	4528114,24
Quercus robur	60325,87	4618958,24
Quercus robur	53238,36	4617612,10
Quercus robur	85907,47	4522511,15
Quercus robur	98875,66	4506558,61
Quercus robur	28616,15	4609269,66
Quercus robur	89536,08	4496073,50
Quercus robur	50409,68	4632520,27
Quercus robur	48798,37	4631774,31
Quercus robur	48261,02	4626243,24
Quercus robur	47642,07	4622468,23
Quercus robur	54477,79	4507360,97
Quercus robur	44262,22	4483006,55
Quercus robur	35025,47	4528715,43
Quercus robur	28918,35	4634597,43
Quercus robur	26272,81	4632038,92
Quercus robur	25539,24	4631708,25
Quercus robur	88269,58	4590478,19
Quercus robur	87985,25	4590438,67
Quercus robur	49975,80	4634610,74
Quercus robur	45576,53	4650180,79
Quercus robur	46116,08	4648160,97
Quercus robur	44804,16	4652215,63
Quercus robur	40258,97	4653691,00
Quercus robur	24072,99	4657380,72
Quercus robur	63356,49	4549122,07
Quercus robur	56989,76	4532236,10
Quercus robur	50375,62	4534420,40
Quercus robur	66475,83	4676547,50
Quercus robur	43021,04	4614764,63
Quercus robur	43023,66	4437229,07
Quercus robur	72466,35	4608214,57
Quercus robur	66703,01	4601268,75
Quercus robur	63874,72	4647339,02
Quercus robur	61377,99	4643969,24
Quercus robur	55116,18	4642518,04
Quercus robur	49875,83	4643115,12
Quercus robur	45237,98	4530822,09
Quercus robur	24727,35	4629307,50
Quercus robur	28971,88	4626349,05
Quercus robur	54734,97	4536697,40

Quercus robur	89462,27	4582989,54
Quercus robur	18234,56	4651358,16
Quercus robur	74361,50	4570820,18
Quercus robur	77911,44	4562611,99
Quercus robur	71203,55	4576132,53
Quercus robur	31680,03	4631158,10
Quercus robur	34248,00	4630108,98
Quercus robur	16884,37	4648124,14
Quercus robur	16523,50	4644164,49
Quercus robur	17719,31	4644128,00
Quercus robur	80250,19	4576139,46
Quercus robur	15219,15	4636217,79
Quercus robur	16528,49	4644164,15
Quercus robur	36359,63	4627339,12
Quercus robur	17093,71	4626616,22
Quercus robur	20096,68	4624539,58
Quercus robur	28909,40	4609585,14
Quercus robur	37999,76	4614840,48
Quercus robur	35349,71	4652895,22
Quercus robur	33015,59	4652501,76
Quercus robur	22691,68	4652083,81
Quercus robur	42549,64	4591362,58
Quercus robur	31108,22	4643980,95
Quercus robur	70200,43	4498467,07
Quercus robur	79267,21	4498852,08
Quercus robur	70200,43	4498467,07
Quercus robur	29006,27	4517531,09
Quercus robur	81345,79	4463406,65
Quercus robur	60286,85	4556752,02
Quercus robur	60006,98	4554160,31
Quercus robur	129127,90	4508505,20
Quercus robur	26882,77	4624835,60
Quercus robur	26882,77	4624835,60
Quercus robur	97627,75	4640367,86
Quercus robur	104141,88	4640972,09
Quercus robur	92230,08	4637543,56
Quercus robur	35693,67	4601380,69
Quercus robur	45619,35	4609960,38
Quercus robur	50186,94	4608502,43
Quercus robur	39387,92	4649820,92
Quercus robur	32145,20	4639104,30
Quercus robur	60876,29	4626472,68
Quercus robur	62319,39	4627008,62
Quercus robur	70625,31	4632703,34
Quercus robur	69076,65	4625405,59
Quercus robur	68813,54	4624451,57
Quercus robur	64615,84	4620948,54
Quercus robur	37283,19	4670651,76
Quercus robur	54473,62	4672177,65
Quercus robur	62155,37	4675298,28

Quercus robur	53828,32	4629451,18
Quercus robur	61540,39	4635897,92
Quercus robur	27030,21	4651514,50
Quercus robur	17851,96	4626676,69
Quercus robur	21189,53	4602306,64
Quercus robur	47493,02	4562148,62
Quercus robur	65258,18	4513710,44
Quercus robur	64708,79	4513330,30
Quercus robur	91213,00	4607281,29
Quercus robur	99695,83	4609801,45
Quercus robur	98176,86	4612337,29
Quercus robur	67191,51	4628830,36
Quercus robur	67827,21	4638234,50
Quercus robur	85550,88	4627898,64
Quercus robur	93740,96	4652166,45
Quercus robur	24210,36	4629910,93
Quercus robur	38329,84	4519207,86
Quercus robur	45745,99	4519451,55
Quercus robur	19594,45	4644123,94
Quercus robur	21931,97	4642091,87
Quercus robur	23797,88	4642669,84
Quercus robur	90203,64	4615105,39
Quercus robur	137028,05	4524968,26
Quercus robur	68194,28	4521819,43
Quercus robur	91789,69	4607304,27
Quercus robur	31098,66	4665358,51
Quercus robur	31286,11	4665625,01
Quercus robur	39987,46	4670173,78
Quercus robur	37875,05	4668426,41
Quercus robur	51407,27	4627338,91
Quercus robur	56661,97	4593617,21
Quercus robur	37471,01	4663432,67
Quercus robur	36470,64	4661858,22
Quercus robur	79697,83	4629522,04
Quercus robur	27041,64	4551709,77
Quercus robur	28651,58	4662519,76
Quercus robur	31770,77	4666597,04
Quercus robur	41552,62	4630919,49
Quercus robur	78940,34	4577129,05
Quercus robur	75742,26	4543903,94
Quercus robur	100560,80	4481029,26
Quercus robur	-56696,32	4310662,28
Quercus robur	44920,46	4525367,04
Quercus robur	61469,05	4647230,58
Quercus robur	59130,13	4645122,86
Quercus robur	59918,80	4650437,37
Quercus robur	59078,21	4650534,11
Quercus robur	58156,47	4643677,93
Quercus robur	37431,19	4497341,13
Quercus robur	46068,22	4660990,74

Quercus robur	44198,48	4663765,63
Quercus robur	46452,33	4658891,52
Quercus robur	23184,73	4572966,23
Quercus robur	92405,25	4469650,39
Quercus robur	49728,77	4544360,46
Quercus robur	38244,35	4523127,27
Quercus robur	55881,67	4661606,08
Quercus robur	54020,97	4660507,52
Quercus robur	51139,07	4657332,72
Quercus robur	51511,85	4648654,85
Quercus robur	97712,90	4470110,73
Quercus robur	45702,37	4669837,10
Quercus robur	46432,28	4671931,74
Quercus robur	47233,27	4671980,67
Quercus robur	43452,26	4658593,47
Quercus robur	40021,27	4656539,79
Quercus robur	80952,57	4571418,54
Quercus robur	81124,98	4571620,35
Quercus robur	84024,38	4577538,27
Quercus robur	52003,37	4589377,33
Quercus robur	57619,30	4592956,52
Quercus robur	59385,85	4601366,48
Quercus robur	28694,41	4643883,77
Quercus robur	28536,06	4644139,72
Quercus robur	66287,16	4597837,99
Quercus robur	70183,72	4610747,17
Quercus robur	45076,07	4669174,71
Quercus robur	16993,47	4617371,95
Quercus robur	18377,29	4609791,51
Quercus robur	46920,83	4434398,51
Quercus robur	56451,16	4432153,90
Quercus robur	58935,40	4430794,81
Quercus robur	61337,75	4501079,01
Quercus robur	116572,20	4559555,14
Quercus robur	45041,52	4483104,09
Quercus robur	73203,12	4562116,74
Quercus robur	53429,72	4529106,44
Quercus robur	57162,30	4528324,14
Quercus robur	53402,37	4513155,40
Quercus robur	53381,26	4513100,93
Quercus robur	91457,92	4495522,35
Quercus robur	91731,45	4485994,39
Quercus robur	91729,15	4485905,40
Quercus robur	79762,44	4486969,34
Quercus robur	49727,85	4633990,70
Quercus robur	32905,42	4651828,46
Quercus robur	32949,69	4571005,86
Quercus robur	78517,19	4487852,78
Quercus robur	93119,29	4496021,89
Quercus robur	95657,97	4492920,88

Quercus robur	94611,86	4494124,94
Quercus robur	19801,22	4605958,36
Quercus robur	40775,31	4617573,59
Quercus robur	103768,13	4501094,93
Quercus robur	18328,58	4621589,56
Quercus robur	18582,81	4623179,37
Quercus robur	38904,86	4481517,08
Quercus robur	76885,21	4551582,98
Quercus robur	59994,32	4554974,84
Quercus robur	84213,50	4528165,97
Quercus robur	87527,38	4603457,67
Quercus robur	87404,48	4605481,66
Quercus robur	40074,79	4602672,64
Quercus robur	40300,38	4603104,39
Quercus robur	40805,29	4604009,08
Quercus robur	44718,16	4612024,83
Quercus robur	74788,96	4529861,94
Quercus robur	77110,32	4530876,26
Quercus robur	84014,36	4516868,35
Quercus robur	32390,14	4590617,70
Quercus robur	29099,07	4592102,79
Quercus robur	24422,40	4593312,39
Quercus robur	22309,75	4657432,39
Quercus robur	70934,70	4638927,45
Quercus robur	43796,90	4658604,73
Quercus robur	72075,33	4618760,38
Quercus robur	72926,32	4617751,04
Quercus robur	68739,58	4622070,40
Quercus robur	69338,07	4567682,59
Quercus robur	77945,23	4548557,61
Quercus robur	98837,45	4486835,04
Quercus robur	23327,36	4642277,30
Quercus robur	19263,37	4605860,33
Quercus robur	69114,50	4599262,72
Quercus robur	68862,54	4597572,28
Quercus robur	74561,85	4600466,51
Quercus robur	76305,80	4600687,09
Quercus robur	43535,87	4651661,30
Quercus robur	63021,92	4676203,44
Quercus robur	76234,82	4627463,82
Quercus robur	93303,04	4636512,81
Quercus robur	73150,34	4637557,05
Quercus robur	100701,60	4484307,28
Quercus robur	97287,25	4631127,82
Quercus robur	99708,39	4629665,87
Quercus robur	107350,32	4625186,89
Quercus robur	82487,39	4626460,83
Quercus robur	88358,85	4458263,79
Quercus robur	78294,32	4469793,67
Quercus robur	78294,32	4469793,67

Quercus robur	85885,05	4463256,03
Quercus robur	87817,70	4463617,89
Quercus robur	81885,94	4463521,48
Quercus robur	83360,76	4470458,14
Quercus robur	87310,63	4632276,81
Quercus robur	99554,02	4606756,68
Quercus robur	99935,73	4609810,38
Quercus robur	103017,99	4609862,37
Quercus robur	77361,37	4584999,75
Quercus robur	67016,19	4537725,86
Quercus robur	102178,14	4613997,42
Quercus robur	101113,27	4612853,27
Quercus robur	35187,05	4512401,24
Quercus robur	98176,02	4612337,33
Quercus robur	101906,31	4610781,66
Quercus robur	34792,30	4670201,91
Quercus robur	18167,59	4655224,07
Quercus robur	22493,89	4649240,42
Quercus robur	22282,96	4648808,24
Quercus robur	58944,00	4447633,42
Quercus robur	120169,83	4361017,13
Quercus robur	56492,99	4432017,73
Quercus robur	49650,19	4441559,48
Quercus robur	49666,81	4441536,20
Quercus robur	57827,78	4447464,48
Quercus robur	56503,90	4432028,23
Quercus robur	45178,04	4427758,62
Quercus robur	48591,96	4560040,12
Quercus robur	132322,11	4639995,90
Quercus robur	87647,20	4620656,48
Quercus robur	87095,91	4619707,56
Quercus robur	88388,22	4620792,18
Quercus robur	88259,46	4620966,73
Quercus robur	39204,95	4635164,20
Quercus robur	68901,40	4607546,63
Quercus robur	64974,48	4614014,76
Quercus robur	51607,73	4540354,51
Quercus robur	56532,95	4539398,11
Quercus robur	72574,59	4539585,22
Quercus robur	56678,39	4514965,33
Quercus robur	62341,88	4522252,63
Quercus robur	58387,09	4530257,24
Quercus robur	26141,31	4652265,57
Quercus robur	46796,73	4534249,45
Quercus robur	112870,63	4604246,03
Quercus robur	98579,89	4602499,30
Quercus robur	95254,56	4602195,03
Quercus robur	94768,79	4494962,97
Quercus robur	98295,22	4601044,52
Quercus robur	100630,41	4582299,16

Quercus robur	56026,05	4673384,54
Quercus robur	63852,32	4676742,94
Quercus robur	64589,43	4676608,13
Quercus robur	33618,46	4626557,91
Quercus robur	33696,21	4639359,66
Quercus robur	20910,66	4623804,42
Quercus robur	30274,59	4612586,05
Quercus robur	59363,60	4516287,70
Quercus robur	58539,84	4611943,28
Quercus robur	101388,70	4487790,05
Quercus robur	93822,40	4474006,77
Quercus robur	56456,92	4515335,28
Quercus robur	60441,97	4522911,50
Quercus robur	87393,06	4485686,62
Quercus robur	87455,50	4485660,90
Quercus robur	92886,12	4495176,90
Quercus robur	106790,15	4496012,24
Quercus robur	92812,15	4495426,00
Quercus robur	47064,12	4614608,91
Quercus robur	28214,76	4517436,46
Quercus robur	110397,80	4500433,14
Quercus robur	41820,60	4472229,43
Quercus robur	41820,60	4472229,43
Quercus robur	41820,60	4472229,43
Quercus robur	41820,60	4472229,43
Quercus robur	41820,60	4472229,43
Quercus robur	41820,60	4472229,43
Quercus robur	41820,60	4472229,43
Quercus robur	40628,82	4462491,04
Quercus robur	96024,26	4470702,51
Quercus robur	35848,28	4565344,33
Quercus robur	36737,15	4624124,38
Quercus robur	86219,69	4530950,00
Quercus robur	52166,38	4521735,92
Quercus robur	26800,68	4570688,85
Quercus robur	120908,46	4625179,75
Quercus robur	53747,76	4507627,72
Quercus robur	96825,39	4470548,11
Quercus robur	96253,21	4470757,03
Quercus robur	70374,93	4559529,65
Quercus robur	70374,93	4559529,65
Quercus robur	39554,76	4670347,01
Quercus robur	70457,81	4633070,14
Quercus robur	98970,73	4480011,59
Quercus robur	95026,24	4493957,63
Quercus robur	51158,52	4657298,03
Quercus robur	57348,90	4661837,54
Quercus robur	22705,75	4623930,06
Quercus robur	26052,86	4661599,82
Quercus robur	97642,55	4477532,22

Quercus robur	98213,28	4477512,76
Quercus robur	77107,09	4524937,09
Quercus robur	75210,50	4539454,78
Quercus robur	40675,74	4670541,70
Quercus robur	101534,73	4482302,71
Quercus robur	22779,46	4580300,25
Quercus robur	33702,90	4618208,16
Quercus robur	69899,95	4560817,05
Quercus robur	38767,40	4615025,38
Quercus robur	54173,96	4672129,67
Quercus robur	50044,41	4672927,28
Quercus robur	79939,73	4626074,98
Quercus robur	90812,30	4485532,21
Quercus robur	75273,23	4626818,63
Quercus robur	93352,70	4639852,94
Quercus robur	98690,20	4640775,88
Quercus robur	47539,85	4655319,79
Quercus robur	53357,17	4644680,10
Quercus robur	62040,25	4675182,78
Quercus robur	54443,50	4672134,94
Quercus robur	64938,29	4561579,05
Quercus robur	23925,07	4661340,90
Quercus robur	49792,96	4672932,17
Quercus robur	62154,58	4456184,06
Quercus robur	65150,92	4622900,49
Quercus robur	83174,73	4454537,99
Quercus robur	57718,27	4455662,42
Quercus robur	72370,57	4452289,50
Quercus robur	83719,10	4596176,76
Quercus robur	63419,45	4622113,90
Quercus robur	84103,79	4596199,17
Quercus robur	81185,23	4625968,53
Quercus robur	83633,09	4595747,10
Quercus robur	81697,31	4625537,35
Quercus robur	74524,68	4452123,40
Quercus robur	83408,29	4582454,42
Quercus robur	44830,07	4641651,42
Quercus robur	86075,75	4623065,06
Quercus robur	83200,96	4628826,52
Quercus robur	75348,67	4629734,55
Quercus robur	105034,74	4647038,95
Quercus robur	36448,93	4553015,78
Quercus robur	36473,49	4551988,19
Quercus robur	37589,77	4547456,76
Quercus robur	49692,59	4544708,31
Quercus robur	50452,91	4544862,41
Quercus robur	55388,26	4642947,09
Quercus robur	59640,97	4643284,86
Quercus robur	61933,16	4643165,71
Quercus robur	63477,90	4642569,07

Quercus robur	81952,25	4588990,53
Quercus robur	82517,97	4586216,49
Quercus robur	80115,76	4584806,24
Quercus robur	59760,21	4591521,46
Quercus robur	62760,41	4595765,33
Quercus robur	62906,71	4594797,74
Quercus robur	59912,52	4632474,76
Quercus robur	73954,93	4640685,87
Quercus robur	74797,62	4640858,45
Quercus robur	78138,38	4640024,44
Quercus robur	27123,63	4625377,54
Quercus robur	63013,57	4549945,07
Quercus robur	88674,82	4643944,00
Quercus robur	87904,88	4643542,78
Quercus robur	87609,03	4646000,57
Quercus robur	64474,98	4502878,65
Quercus robur	111498,97	4559578,16
Quercus robur	39368,17	4626140,90
Quercus robur	53650,96	4574624,82
Quercus robur	55753,34	4590473,05
Quercus robur	29397,42	4507881,29
Quercus robur	38623,29	4649859,38
Quercus robur	39982,08	4653764,73
Quercus robur	40727,38	4652790,59
Quercus robur	45743,07	4651285,50
Quercus robur	48263,86	4647633,78
Quercus robur	96241,30	4533090,22
Quercus robur	96241,30	4533090,22
Quercus robur	55390,44	4558943,28
Quercus robur	55390,44	4558943,28
Quercus robur	78554,76	4540354,41
Quercus robur	84721,08	4538755,61
Quercus robur	77570,08	4539608,59
Quercus robur	77570,08	4539608,59
Quercus robur	76386,54	4538629,17
Quercus robur	83321,68	4561443,18
Quercus robur	82445,55	4544790,01
Quercus robur	82407,68	4542608,21
Quercus robur	96959,65	4560555,26
Quercus robur	97956,23	4561436,10
Quercus robur	97956,23	4561436,10
Quercus robur	97956,23	4561436,10
Quercus robur	106544,97	4558540,10
Quercus robur	79359,45	4540130,04
Quercus robur	71958,28	4537960,52
Quercus robur	72181,41	4538248,45
Quercus robur	64558,08	4546921,65
Quercus robur	87634,92	4537767,20
Quercus robur	82225,52	4542451,42
Quercus robur	82400,94	4542653,16

Quercus robur	82400,94	4542653,16
Quercus robur	82830,70	4543330,72
Quercus robur	74739,01	4552219,90
Quercus robur	75078,77	4554975,14
Quercus robur	74222,09	4556596,33
Quercus robur	82132,03	4541342,47
Quercus robur	81332,32	4542067,70
Quercus robur	76447,78	4543462,03
Quercus robur	38663,02	4524874,17
Quercus robur	39358,06	4524786,21
Quercus robur	40527,99	4524479,21
Quercus robur	48226,57	4470827,16
Quercus robur	69837,86	4560764,98
Quercus robur	75381,58	4555336,47
Quercus robur	39315,71	4563440,58
Quercus robur	37448,29	4489646,21
Quercus robur	41152,84	4639868,47
Quercus robur	60359,67	4643563,78
Quercus robur	60708,16	4643642,61
Quercus robur	61919,72	4643177,68
Quercus robur	36448,19	4489719,33
Quercus robur	33716,22	4638822,86
Quercus robur	116201,55	4446085,78
Quercus robur	85870,18	4448942,61
Quercus robur	27022,65	4462674,62
Quercus robur	69347,86	4648064,41
Quercus robur	26023,25	4613121,74
Quercus robur	58932,52	4506816,55
Quercus robur	62741,07	4509076,95
Quercus robur	56872,64	4450150,54
Quercus robur	21762,02	4631513,73
Quercus robur	24101,55	4632986,60
Quercus robur	24101,55	4632986,60
Quercus robur	25378,02	4633850,07
Quercus robur	47105,81	4531119,70
Quercus robur	48328,72	4530130,46
Quercus robur	55737,31	4448222,11
Quercus robur	67254,48	4573056,00
Quercus robur	66643,76	4572613,04
Quercus robur	66514,01	4570235,37
Quercus robur	56771,20	4478287,81
Quercus robur	21790,41	4601731,22
Quercus robur	29777,83	4650316,71
Quercus robur	65283,53	4568190,81
Quercus robur	31308,55	4638701,85
Quercus robur	31980,81	4638903,12
Quercus robur	54911,21	4653225,14
Quercus robur	55261,86	4655176,99
Quercus robur	55560,12	4654912,95
Quercus robur	55541,24	4654412,32

Quercus robur	55249,41	4654274,49
Quercus robur	186814,05	4646382,02
Quercus robur	185184,01	4645986,72
Quercus robur	38569,55	4615818,88
Quercus robur	131770,54	4578925,95
Quercus robur	63446,86	4642593,27
Quercus robur	38587,75	4616141,18
Quercus robur	94823,02	4634108,58
Quercus robur	39589,57	4535934,13
Quercus robur	36118,78	4532928,99
Quercus robur	36132,18	4532805,48
Quercus robur	36146,70	4532726,50
Quercus robur	36263,31	4532708,01
Quercus robur	22072,32	4644816,36
Quercus robur	41737,63	4539301,54
Quercus robur	41959,25	4538986,66
Quercus robur	39413,36	4563824,74
Quercus robur	39298,13	4564311,57
Quercus robur	39287,18	4563977,69
Quercus robur	101732,85	4641797,22
Quercus robur	100295,28	4641810,94
Quercus robur	43086,44	4620381,66
Quercus robur	49736,46	4448621,19
Quercus robur	41069,41	4565259,40
Quercus robur	38734,45	4565150,15
Quercus robur	37847,36	4563756,44
Quercus robur	34393,68	4624198,27
Quercus robur	41935,25	4619239,39
Quercus robur	39007,56	4616560,37
Quercus robur	97104,94	4584599,75
Quercus robur	40665,34	4572054,19
Quercus robur	23238,78	4487605,91
Quercus robur	22170,29	4486670,27
Quercus robur	22267,80	4631848,13
Quercus robur	117995,69	4600887,34
Quercus robur	41275,81	4524544,27
Quercus robur	27464,66	4659519,29
Quercus robur	52844,04	4672738,04
Quercus robur	95132,20	4581267,02
Quercus robur	91078,75	4591076,56
Quercus robur	90959,30	4590983,04
Quercus robur	48610,45	4560038,98
Quercus robur	49006,14	4560215,25
Quercus robur	21144,50	4624759,56
Quercus robur	43364,66	4618769,04
Quercus robur	42187,12	4620338,65
Quercus robur	62390,63	4618386,00
Quercus robur	65425,21	4619271,76
Quercus robur	66515,45	4618302,77
Quercus robur	21881,49	4587488,56

Quercus robur	52060,12	4617540,59
Quercus robur	54376,65	4451188,78
Quercus robur	54908,67	4450934,55
Quercus robur	76652,15	4583993,50
Quercus robur	89701,76	4595165,87
Quercus robur	89469,27	4594543,95
Quercus robur	42223,90	4553422,03
Quercus robur	27919,35	4634797,31
Quercus robur	28474,03	4634994,94
Quercus robur	77348,47	4629426,52
Quercus robur	78714,32	4628933,49
Quercus robur	82327,36	4627740,71
Quercus robur	50612,17	4627366,51
Quercus robur	144952,18	4640679,93
Quercus robur	145193,59	4640445,22
Quercus robur	50643,63	4629516,84
Quercus robur	76346,38	4608118,84
Quercus robur	71992,88	4603672,62
Quercus robur	73785,27	4605316,33
Quercus robur	75580,39	4606380,64
Quercus robur	76272,97	4606885,99
Quercus robur	79795,87	4606579,19
Quercus robur	73200,80	4664509,12
Quercus robur	43150,62	4554992,23
Quercus robur	51766,34	4658876,67
Quercus robur	51660,91	4658894,50
Quercus robur	26569,27	4612985,34
Quercus robur	39989,17	4618427,02
Quercus robur	-59406,18	4310294,89
Quercus robur	55893,40	4661594,19
Quercus robur	54903,57	4661321,82
Quercus robur	54874,30	4661345,97
Quercus robur	54786,83	4661318,01
Quercus robur	54735,71	4661298,93
Quercus robur	54802,40	4661261,27
Quercus robur	54870,19	4661201,25
Quercus robur	54765,19	4661185,55
Quercus robur	54883,93	4661088,87
Quercus robur	54866,21	4661045,37
Quercus robur	54871,14	4660978,15
Quercus robur	54905,65	4660931,37
Quercus robur	54937,84	4660940,50
Quercus robur	55043,02	4660800,07
Quercus robur	54717,68	4661210,84
Quercus robur	54625,55	4661227,79
Quercus robur	55124,34	4661140,66
Quercus robur	55054,42	4661312,34
Quercus robur	54736,68	4661499,60
Quercus robur	54815,16	4661516,97
Quercus robur	54413,55	4661653,76

Quercus robur	54352,89	4661813,71
Quercus robur	54185,66	4661880,01
Quercus robur	54307,72	4661638,12
Quercus robur	54247,70	4661385,41
Quercus robur	54081,86	4661473,92
Quercus robur	53976,71	4661614,37
Quercus robur	53984,22	4661680,81
Quercus robur	53523,67	4661542,57
Quercus robur	53085,59	4661246,81
Quercus robur	52992,44	4661353,06
Quercus robur	52875,51	4661438,51
Quercus robur	55669,45	4661552,49
Quercus robur	50960,28	4661314,42
Quercus robur	50913,12	4661384,33
Quercus robur	50810,47	4661379,69
Quercus robur	50452,09	4661279,76
Quercus robur	50709,36	4661609,16
Quercus robur	50900,70	4661608,17
Quercus robur	51035,43	4661633,08
Quercus robur	51120,55	4661649,99
Quercus robur	51164,27	4661591,45
Quercus robur	51069,16	4661430,19
Quercus robur	51314,04	4661604,26
Quercus robur	51688,12	4661335,19
Quercus robur	51775,73	4661351,95
Quercus robur	51966,67	4661239,48
Quercus robur	52089,52	4661142,49
Quercus robur	52133,60	4661273,53
Quercus robur	52411,08	4661200,21
Quercus robur	54427,40	4661530,22
Quercus robur	54426,26	4661485,68
Quercus robur	54414,35	4661441,82
Quercus robur	54546,62	4661533,86
Quercus robur	54535,93	4661456,47
Quercus robur	54532,64	4661311,70
Quercus robur	54547,69	4661299,61
Quercus robur	54554,58	4661276,87
Quercus robur	54580,79	4661230,61
Quercus robur	54551,23	4661422,05
Quercus robur	73514,69	4637412,63
Quercus robur	73494,07	4637402,71
Quercus robur	73632,26	4637383,30
Quercus robur	73632,26	4637383,30
Quercus robur	73632,26	4637383,30
Quercus robur	73675,17	4637347,29
Quercus robur	75433,09	4637231,25
Quercus robur	73990,50	4637094,36
Quercus robur	71579,34	4636659,15
Quercus robur	73366,59	4636373,65
Quercus robur	72862,64	4636403,81

Quercus robur	72862,64	4636403,81
Quercus robur	72925,17	4636388,92
Quercus robur	72883,59	4636391,41
Quercus robur	73358,11	4636329,57
Quercus robur	73477,20	4636199,83
Quercus robur	72659,05	4635736,02
Quercus robur	72698,47	4635711,36
Quercus robur	116614,85	4478909,97
Quercus robur	113843,05	4470567,58
Quercus robur	72849,00	4637207,24
Quercus robur	72862,64	4636403,81
Quercus robur	73358,11	4636329,57
Quercus robur	73355,95	4636307,40
Quercus robur	72612,64	4635727,65
Quercus robur	72963,31	4639073,14
Quercus robur	76598,73	4639491,57
Quercus robur	77183,95	4639991,85
Quercus robur	76541,43	4640141,49
Quercus robur	75969,83	4640543,33
Quercus robur	75024,68	4640778,01
Quercus robur	-57347,89	4313153,63
Quercus robur	18250,73	4609956,20
Quercus robur	57763,26	4542958,16
Quercus robur	59336,32	4541002,03
Quercus robur	59187,01	4530900,51
Quercus robur	59120,33	4531428,41
Quercus robur	58848,33	4531199,44
Quercus robur	59957,12	4534733,64
Quercus robur	46836,77	4579283,58
Quercus robur	17732,20	4626216,07
Quercus robur	47016,97	4579406,16
Quercus robur	21704,97	4642218,70
Quercus robur	20205,69	4643814,89
Quercus robur	19623,12	4606081,81
Quercus robur	58620,76	4516510,27
Quercus robur	42690,55	4619726,55
Quercus robur	39657,35	4627761,97
Quercus robur	63372,16	4523317,46
Quercus robur	63235,85	4523593,02
Quercus robur	131764,95	4578915,10
Quercus robur	72591,95	4661201,46
Quercus robur	72552,77	4663333,12
Quercus robur	73093,21	4666800,99
Quercus robur	81335,95	4577826,42
Quercus robur	83162,40	4577921,98
Quercus robur	84838,77	4577380,26
Quercus robur	84910,76	4577153,28
Quercus robur	85024,83	4576823,62
Quercus robur	84098,01	4577634,35
Quercus robur	44954,13	4657214,13

Quercus robur	44916,83	4656736,90
Quercus robur	43460,64	4652357,69
Quercus robur	63864,72	4671468,05
Quercus robur	61570,90	4668042,01
Quercus robur	59847,67	4665651,39
Quercus robur	59863,98	4664657,94
Quercus robur	58158,21	4664563,62
Quercus robur	58389,48	4664538,02
Quercus robur	54301,14	4664794,59
Quercus robur	23777,21	4608930,01
Quercus robur	99797,24	4609171,90
Quercus robur	51698,72	4635762,47
Quercus robur	53933,90	4637384,29
Quercus robur	48675,51	4632038,57
Quercus robur	80308,07	4625730,15
Quercus robur	80059,47	4626079,10
Quercus robur	65948,66	4677973,73
Quercus robur	65892,58	4677966,03
Quercus robur	84765,80	4624935,15
Quercus robur	67245,10	4677827,01
Quercus robur	91116,92	4629361,14
Quercus robur	72933,06	4667602,19
Quercus robur	73079,56	4667125,11
Quercus robur	74568,91	4640883,25
Quercus robur	73732,53	4641067,03
Quercus robur	25003,33	4571910,25
Quercus robur	29364,36	4601935,91
Quercus robur	24692,69	4602331,53
Quercus robur	28766,81	4582709,26
Quercus robur	67492,60	4677789,50
Quercus robur	51075,15	4453557,54
Quercus robur	111346,69	4512733,44
Quercus robur	34621,80	4565690,14
Quercus robur	52919,72	4560532,22
Quercus robur	50668,51	4565163,69
Quercus robur	19955,60	4644043,78
Quercus robur	20158,32	4643929,68
Quercus robur	70180,62	4630433,66
Quercus robur	76615,75	4640493,76
Quercus robur	77652,26	4574462,25
Quercus robur	77195,34	4574622,53
Quercus robur	77255,71	4574184,38
Quercus robur	153701,56	4648787,38
Quercus robur	155034,32	4647364,40
Quercus robur	58141,85	4540393,73
Quercus robur	58869,02	4539937,62
Quercus robur	57877,79	4532372,29
Quercus robur	57883,52	4532594,90
Quercus robur	57145,29	4532315,94
Quercus robur	57454,26	4532341,97

Quercus robur	47488,67	4579276,39
Quercus robur	58844,40	4581688,29
Quercus robur	60011,80	4581450,20
Quercus robur	59906,57	4582170,08
Quercus robur	17661,55	4643953,36
Quercus robur	19360,74	4642789,43
Quercus robur	57557,64	4594844,51
Quercus robur	45663,69	4613950,14
Quercus robur	40200,44	4626087,39
Quercus robur	113699,00	4605716,37
Quercus robur	105035,32	4604894,23
Quercus robur	18949,90	4643988,92
Quercus robur	19359,56	4642041,83
Quercus robur	19509,75	4641507,21
Quercus robur	86602,34	4615189,28
Quercus robur	19457,60	4627104,33
Quercus robur	19337,77	4626666,02
Quercus robur	22022,25	4623674,37
Quercus robur	40471,93	4618786,46
Quercus robur	63058,43	4491348,39
Quercus robur	65557,70	4498636,05
Quercus robur	37448,29	4489646,21
Quercus robur	44041,46	4483176,07
Quercus robur	45109,90	4484103,31
Quercus robur	44041,46	4483176,07
Quercus robur	85471,59	4605146,91
Quercus robur	84778,08	4609377,01
Quercus robur	86722,10	4614914,93
Quercus robur	87125,14	4614735,73
Quercus robur	84165,10	4578644,55
Quercus robur	85091,32	4576028,67
Quercus robur	83811,53	4586899,84
Quercus robur	67735,69	4524777,36
Quercus robur	29309,33	4614155,27
Quercus robur	98735,46	4564634,98
Quercus robur	79756,61	4524407,06
Quercus robur	26851,56	4520077,59
Quercus robur	69705,58	4520605,75
Quercus robur	35695,86	4656475,96
Quercus robur	35747,64	4656706,85
Quercus robur	29813,93	4505836,30
Quercus robur	33138,50	4648968,23
Quercus robur	41950,08	4649922,95
Quercus robur	40741,77	4657374,31
Quercus robur	73371,81	4640085,37
Quercus robur	43232,09	4659879,27
Quercus robur	30787,44	4637865,91
Quercus robur	35819,38	4656568,27
Quercus robur	30596,28	4639340,01
Quercus robur	68655,75	4678108,37

Quercus robur	36706,31	4652326,84
Quercus robur	30646,62	4652356,77
Quercus robur	34181,40	4656162,53
Quercus robur	35647,17	4656088,69
Quercus robur	64330,27	4676847,16
Quercus robur	73632,63	4640415,32
Quercus robur	35518,97	4655963,21
Quercus robur	38424,08	4655806,96
Quercus robur	101108,11	4639793,26
Quercus robur	31346,18	4653392,78
Quercus robur	38030,61	4654895,52
Quercus robur	33523,72	4655525,22
Quercus robur	77129,33	4640195,73
Quercus robur	30962,19	4639594,84
Quercus robur	32695,78	4653393,02
Quercus robur	64016,18	4676855,45
Quercus robur	75580,51	4640700,28
Quercus robur	101094,40	4639816,31
Quercus robur	20816,93	4649018,53
Quercus robur	62827,29	4675970,20
Quercus robur	73675,85	4640691,42
Quercus robur	28165,13	4517417,32
Quercus robur	76601,77	4640539,18
Quercus robur	28744,24	4515573,64
Quercus robur	116461,02	4630796,64
Quercus robur	41739,38	4658748,70
Quercus robur	73709,76	4640700,54
Quercus robur	36450,69	4656616,21
Quercus robur	62296,55	4675512,53
Quercus robur	43181,43	4651684,10
Quercus robur	18523,26	4652097,45
Quercus robur	75052,60	4640798,64
Quercus robur	41075,77	4658390,08
Quercus robur	34161,71	4655304,78
Quercus robur	43314,22	4651597,47
Quercus robur	32757,29	4653567,48
Quercus robur	70779,27	4631958,34
Quercus robur	73495,66	4640300,90
Quercus robur	31818,84	4653506,61
Quercus robur	43915,63	4653031,15
Quercus robur	36409,75	4656830,85
Quercus robur	44370,81	4654396,16
Quercus robur	75086,50	4640807,77
Quercus robur	32567,26	4648804,97
Quercus robur	73788,88	4640628,92
Quercus robur	35582,69	4655523,95
Quercus robur	76636,85	4640525,95
Quercus robur	18720,47	4652742,51
Quercus robur	32558,18	4648984,07
Quercus robur	27253,21	4663137,49

Quercus robur	42210,86	4658506,27
Quercus robur	30989,10	4651798,60
Quercus robur	27163,86	4663121,14
Quercus robur	27031,78	4651525,56
Quercus robur	65671,39	4678080,03
Quercus robur	71498,61	4642059,48
Quercus robur	76456,21	4640447,51
Quercus robur	70894,27	4631940,28
Quercus robur	75925,78	4610149,87
Quercus robur	39903,23	4649653,66
Quercus robur	38354,92	4655755,68
Quercus robur	71955,49	4642098,88
Quercus robur	26953,19	4651597,74
Quercus robur	72927,77	4639732,97
Quercus robur	39755,52	4571152,54
Quercus robur	35634,68	4656457,66
Quercus robur	31096,15	4639485,62
Quercus robur	34370,56	4656150,10
Quercus robur	75513,19	4640704,29
Quercus robur	66138,86	4513313,36
Quercus robur	32121,00	4649425,64
Quercus robur	70338,91	4679688,94
Quercus robur	69016,31	4678142,03
Quercus robur	29789,30	4505581,34
Quercus robur	43191,64	4671148,09
Quercus robur	64348,06	4676812,61
Quercus robur	35637,05	4655654,26
Quercus robur	30414,01	4652684,56
Quercus robur	31695,08	4653525,94
Quercus robur	35059,60	4657064,32
Quercus robur	75849,55	4611001,46
Quercus robur	73603,04	4640339,06
Quercus robur	44405,54	4654405,08
Quercus robur	29321,89	4652823,84
Quercus robur	44435,68	4654537,00
Quercus robur	63089,57	4512010,02
Quercus robur	31188,86	4651629,21
Quercus robur	61133,13	4498081,74
Quercus robur	70886,28	4631918,47
Quercus robur	13169,94	4650799,51
Quercus robur	75411,29	4640743,81
Quercus robur	43712,12	4651783,82
Quercus robur	40814,87	4655172,06
Quercus robur	29991,63	4510821,43
Quercus robur	33952,55	4655976,75
Quercus robur	27583,92	4651790,06
Quercus robur	31008,33	4638810,84
Quercus robur	30815,44	4506787,90
Quercus robur	38763,07	4650586,55
Quercus robur	29961,60	4506261,91

Quercus robur	30882,44	4638852,60
Quercus robur	39158,66	4573320,43
Quercus robur	34037,38	4655391,04
Quercus robur	30887,74	4665327,87
Quercus robur	30948,34	4638825,95
Quercus robur	75509,20	4640693,38
Quercus robur	74967,66	4640814,86
Quercus robur	77738,40	4640115,02
Quercus robur	31340,86	4651228,68
Quercus robur	37260,44	4655492,36
Quercus robur	21803,82	4642223,22
Quercus robur	35528,81	4655884,47
Quercus robur	71550,80	4642067,48
Quercus robur	38138,94	4657900,45
Quercus robur	32060,37	4507869,24
Quercus robur	55933,77	4486284,62
Quercus robur	64302,02	4676860,06
Quercus robur	31870,01	4666411,95
Quercus robur	77829,27	4640198,80
Quercus robur	43223,16	4651659,10
Quercus robur	42108,41	4649856,97
Quercus robur	32935,59	4649104,29
Quercus robur	30362,15	4504686,40
Quercus robur	35553,44	4655548,18
Quercus robur	30461,42	4652670,26
Quercus robur	30769,58	4639663,30
Quercus robur	43170,05	4660362,92
Quercus robur	31593,51	4649951,29
Quercus robur	75861,11	4640594,39
Quercus robur	30443,16	4652671,47
Quercus robur	32533,24	4648896,46
Quercus robur	98686,88	4640776,07
Quercus robur	39720,83	4649888,58
Quercus robur	32913,34	4649641,26
Quercus robur	18029,08	4651427,90
Quercus robur	38925,25	4649784,00
Quercus robur	31602,22	4653520,91
Quercus robur	29871,74	4510672,89
Quercus robur	31666,03	4650482,04
Quercus robur	75851,18	4610432,92
Quercus robur	35880,92	4652949,71
Quercus robur	30955,67	4651153,70
Quercus robur	75840,56	4610522,71
Quercus robur	38956,60	4649971,60
Quercus robur	32411,29	4649261,49
Quercus robur	33318,19	4648922,96
Quercus robur	42300,72	4651171,95
Quercus robur	75005,73	4640768,00
Quercus robur	31235,15	4653377,80
Quercus robur	29184,28	4505240,44

Quercus robur	41756,39	4649957,75
Quercus robur	33212,57	4648762,55
Quercus robur	75263,19	4640763,79
Quercus robur	43174,10	4651751,49
Quercus robur	73258,98	4640861,30
Quercus robur	74985,94	4640813,77
Quercus robur	71270,29	4636543,95
Quercus robur	69669,58	4541158,93
Quercus robur	35489,77	4655619,29
Quercus robur	68615,24	4678110,85
Quercus robur	31711,85	4650177,78
Quercus robur	73234,66	4639759,17
Quercus robur	39009,30	4573396,80
Quercus robur	68792,50	4678200,33
Quercus robur	32022,81	4649454,42
Quercus robur	26988,78	4651506,11
Quercus robur	30976,71	4651118,84
Quercus robur	31409,48	4651067,96
Quercus robur	32991,39	4657222,61
Quercus robur	28611,78	4516162,03
Quercus robur	39319,72	4649836,49
Quercus robur	20709,08	4643602,43
Quercus robur	34443,97	4652519,49
Quercus robur	40286,15	4649283,07
Quercus robur	32470,22	4649168,36
Quercus robur	30204,68	4505142,45
Quercus robur	39599,34	4571028,58
Quercus robur	72837,34	4639727,24
Quercus robur	43019,21	4659625,30
Quercus robur	76624,72	4640504,37
Quercus robur	32836,03	4649099,67
Quercus robur	73615,18	4640639,31
Quercus robur	58062,37	4459420,46
Quercus robur	39769,50	4571107,04
Quercus robur	63976,89	4676891,33
Quercus robur	32727,56	4653457,87
Quercus robur	40557,41	4650369,85
Quercus robur	35584,05	4655646,58
Quercus robur	70407,92	4679840,82
Quercus robur	30975,26	4638790,71
Quercus robur	73328,76	4640021,07
Quercus robur	30780,91	4638312,60
Quercus robur	38943,38	4573646,34
Quercus robur	74955,86	4640826,71
Quercus robur	-9897,94	4768926,97
Quercus robur	-1321,72	4748200,22
Quercus robur	-607,54	4758205,65
Quercus robur	-178,46	4764208,83
Quercus robur	77760,13	4469327,46
Quercus robur	77760,13	4469327,46

Quercus robur	81352,33	4463057,50
Quercus robur	82826,84	4469987,91
Quercus robur	85351,27	4462786,39
Quercus robur	88011,66	4457584,70
Quercus robur	33304,93	4524605,19
Quercus robur	59760,97	4514754,10
Quercus robur	63308,79	4522548,37
Quercus robur	5839,23	4707468,07
Quercus robur	11615,01	4646733,69
Quercus robur	12316,75	4656738,43
Quercus robur	20137,67	4696402,11
Quercus robur	20996,60	4694330,75
Quercus robur	14429,24	4686751,63
Quercus robur	15135,83	4696755,69
Quercus robur	23023,57	4666039,53
Quercus robur	23727,57	4676042,71
Quercus robur	24432,78	4686045,73
Quercus robur	25139,20	4696048,58
Quercus robur	25846,83	4706051,25
Quercus robur	33730,06	4675338,20
Quercus robur	35848,82	4705343,10
Quercus robur	19389,93	4657247,22
Quercus robur	27705,71	4703909,14
Quercus robur	38449,21	4670985,48
Quercus robur	43660,92	4673633,78
Quercus robur	46872,35	4676422,63
Quercus robur	62353,11	4696433,19
Quercus robur	63989,50	4705360,75
Quercus robur	68917,56	4704007,11
Quercus robur	74797,96	4673451,09
Quercus robur	75901,05	4646248,73
Quercus robur	78320,95	4652106,62
Quercus robur	16043,01	4766065,24
Quercus robur	54436,11	4683929,03
Quercus robur	65847,53	4703219,64
Quercus robur	71622,02	4642529,97
Quercus robur	74432,27	4682518,72
Quercus robur	75137,87	4692515,51
Quercus robur	75844,68	4702512,14
Quercus robur	83021,29	4661822,18
Quercus robur	83724,31	4671818,08
Quercus robur	85133,97	4691809,39
Quercus robur	85840,63	4701804,81
Quercus robur	93719,51	4671114,53
Quercus robur	94423,58	4681109,06
Quercus robur	95128,87	4691103,42
Quercus robur	7258,80	4727477,74
Quercus robur	10112,40	4767495,02
Quercus robur	11546,40	4787502,62
Quercus robur	17974,29	4736770,21

Quercus robur	19400,75	4756776,45
Quercus robur	27265,72	4726056,10
Quercus robur	23687,67	4760491,66
Quercus robur	27976,98	4736058,27
Quercus robur	29403,09	4756062,10
Quercus robur	30117,95	4766063,76
Quercus robur	31551,26	4786066,58
Quercus robur	32269,71	4796067,73
Quercus robur	36557,49	4715344,40
Quercus robur	37267,37	4725345,53
Quercus robur	34618,12	4758705,33
Quercus robur	37978,46	4735346,50
Quercus robur	38690,75	4745347,29
Quercus robur	39404,24	4755347,92
Quercus robur	40118,93	4765348,39
Quercus robur	41551,91	4785348,81
Quercus robur	47267,82	4724635,13
Quercus robur	48690,86	4744634,49
Quercus robur	50118,71	4764633,18
Quercus robur	50834,43	4774632,28
Quercus robur	57267,06	4723924,90
Quercus robur	58689,77	4743921,85
Quercus robur	61549,61	4783913,78
Quercus robur	62986,69	4803908,76
Quercus robur	67975,69	4733212,18
Quercus robur	63549,11	4783770,31
Quercus robur	65407,61	4725356,36
Quercus robur	68472,15	4754277,46
Quercus robur	68687,48	4743209,37
Quercus robur	72983,43	4803189,11
Quercus robur	77972,36	4732501,06
Quercus robur	79396,83	4752492,88
Quercus robur	88679,30	4741784,90
Quercus robur	90105,85	4761773,99
Quercus robur	81979,47	4802541,55
Quercus robur	82752,34	4757276,53
Quercus robur	82184,03	4735215,33
Quercus robur	84751,38	4757133,73
Quercus robur	88252,25	4777979,73
Quercus robur	89677,38	4755777,33
Quercus robur	92891,32	4772624,57
Quercus robur	96104,23	4775407,92
Quercus robur	99473,06	4808313,85
Quercus robur	100544,35	4809241,13
Quercus robur	97672,59	4755206,37
Quercus robur	97886,73	4758204,42
Quercus robur	90200,28	4819027,96
Quercus robur	92126,92	4817884,32
Quercus robur	94197,77	4818739,44
Quercus robur	99771,70	4826373,08

Quercus robur	99843,92	4827372,36
Quercus robur	103564,50	4837148,00
Quercus robur	102903,07	4814093,37
Quercus robur	92973,32	4801750,28
Quercus robur	99385,92	4751066,32
Quercus robur	100099,64	4761059,59
Quercus robur	101530,67	4781045,66
Quercus robur	102966,48	4801031,11
Quercus robur	64428,55	4823903,09
Quercus robur	73703,60	4813185,16
Quercus robur	74424,95	4823181,06
Quercus robur	83698,98	4812464,48
Quercus robur	103686,17	4811023,59
Quercus robur	105129,12	4831008,10
Quercus robur	105852,37	4841000,11
Quercus robur	106576,80	4850991,97
Quercus robur	91934,17	4500501,27
Quercus robur	105828,89	4700390,61
Quercus robur	133688,20	4668301,91
Quercus robur	134391,66	4678291,58
Quercus robur	142975,24	4657610,53
Quercus robur	185028,42	4684756,54
Quercus robur	106536,43	4710383,45
Quercus robur	107245,19	4720376,14
Quercus robur	107955,14	4730368,68
Quercus robur	109378,67	4750353,28
Quercus robur	112240,11	4790320,64
Quercus robur	112958,45	4800312,09
Quercus robur	120798,24	4769621,99
Quercus robur	122231,04	4789602,97
Quercus robur	122949,23	4799593,23
Quercus robur	126519,55	4708967,46
Quercus robur	125782,26	4655806,05
Quercus robur	130356,94	4649461,24
Quercus robur	124946,40	4715103,36
Quercus robur	126370,81	4721026,70
Quercus robur	126512,70	4723024,76
Quercus robur	127440,77	4721954,79
Quercus robur	128510,72	4722882,85
Quercus robur	129296,92	4719814,92
Quercus robur	129367,84	4720813,92
Quercus robur	135145,73	4787671,34
Quercus robur	131365,81	4720672,07
Quercus robur	138713,01	4725170,05
Quercus robur	146271,78	4774825,76
Quercus robur	147198,97	4773755,46
Quercus robur	146769,06	4753708,96
Quercus robur	150906,65	4755421,15
Quercus robur	151192,07	4759415,98
Quercus robur	151905,36	4755349,82

Quercus robur	151976,69	4756348,52
Quercus robur	155623,13	4793227,54
Quercus robur	153474,99	4749215,08
Quercus robur	153831,41	4754208,49
Quercus robur	137927,10	4728237,68
Quercus robur	140062,82	4758203,55
Quercus robur	142927,21	4798155,96
Quercus robur	146497,84	4707552,07
Quercus robur	147205,99	4717539,93
Quercus robur	147915,35	4727527,65
Quercus robur	148625,91	4737515,22
Quercus robur	150050,62	4757489,92
Quercus robur	158194,98	4801072,79
Quercus robur	158410,65	4804068,57
Quercus robur	156757,75	4738943,65
Quercus robur	156900,03	4740940,95
Quercus robur	157897,34	4754921,87
Quercus robur	159326,13	4774894,12
Quercus robur	162404,92	4803780,95
Quercus robur	159751,96	4752782,02
Quercus robur	159753,65	4738730,27
Quercus robur	159967,07	4741726,11
Quercus robur	160750,56	4752710,73
Quercus robur	160041,63	4728673,05
Quercus robur	161820,44	4753638,02
Quercus robur	167677,62	4793364,75
Quercus robur	152196,68	4787450,86
Quercus robur	152914,42	4797437,55
Quercus robur	157193,18	4716831,26
Quercus robur	157902,39	4726817,77
Quercus robur	158612,80	4736804,14
Quercus robur	159324,41	4746790,36
Quercus robur	167677,62	4793364,75
Quercus robur	164320,09	4732383,32
Quercus robur	171032,03	4798141,67
Quercus robur	172669,90	4793005,89
Quercus robur	174017,58	4741728,45
Quercus robur	177590,14	4791648,70
Quercus robur	182014,19	4797351,61
Quercus robur	183084,32	4798278,10
Quercus robur	181077,52	4742228,78
Quercus robur	181575,89	4749217,23
Quercus robur	182503,00	4748147,66
Quercus robur	186510,31	4804052,19
Quercus robur	187923,45	4739734,20
Quercus robur	188850,61	4738664,81
Quercus robur	191629,98	4749503,15
Quercus robur	194624,59	4749289,45
Quercus robur	202409,01	4801904,01
Quercus robur	202396,11	4745725,36

Quercus robur	206257,50	4799620,55
Quercus robur	204534,66	4747579,14
Quercus robur	161466,44	4776748,15
Quercus robur	168598,49	4736093,20
Quercus robur	172885,26	4796001,17
Quercus robur	179294,31	4745366,22
Quercus robur	182151,59	4785300,08
Quercus robur	189277,46	4744654,37
Quercus robur	190703,43	4764619,19
Quercus robur	199971,66	4753923,94
Quercus robur	200685,10	4763905,08
Quercus robur	202832,59	4793847,67
Quercus robur	115614,25	4823219,67
Quercus robur	117841,84	4840131,61
Quercus robur	117756,76	4825073,47
Quercus robur	118539,31	4822004,13
Quercus robur	119261,40	4831994,61
Quercus robur	119466,22	4820932,93
Quercus robur	124389,18	4819573,29
Quercus robur	124677,74	4823569,23
Quercus robur	125748,88	4824496,03
Quercus robur	137386,72	4832691,69
Quercus robur	137375,02	4818635,90
Quercus robur	139228,50	4816494,09
Quercus robur	141671,04	4836397,74
Quercus robur	143512,00	4820200,87
Quercus robur	144450,45	4833184,61
Quercus robur	116567,99	4850267,03
Quercus robur	123668,61	4809583,33
Quercus robur	124389,18	4819573,29
Quercus robur	125110,94	4829563,09
Quercus robur	125833,88	4839552,74
Quercus robur	126558,00	4849542,23
Quercus robur	134378,46	4818852,20
Quercus robur	135100,07	4828840,81
Quercus robur	135822,86	4838829,28
Quercus robur	143646,29	4808143,68
Quercus robur	144366,56	4818131,26
Quercus robur	146075,80	4813992,17
Quercus robur	147952,74	4839958,72
Quercus robur	148927,91	4811778,68
Quercus robur	149143,98	4814774,77
Quercus robur	149998,61	4812705,36
Quercus robur	150070,64	4813704,04
Quercus robur	150719,38	4822692,15
Quercus robur	156362,24	4831318,94
Quercus robur	156134,56	4814270,50
Quercus robur	157277,25	4816195,67
Quercus robur	158914,31	4811058,68
Quercus robur	160345,05	4816978,07

Quercus robur	160777,64	4822969,41
Quercus robur	162126,02	4813838,33
Quercus robur	163484,82	4818758,94
Quercus robur	167685,17	4807415,37
Quercus robur	169610,17	4806273,03
Quercus robur	171833,11	4823174,45
Quercus robur	173613,60	4820035,00
Quercus robur	173891,69	4809978,95
Quercus robur	176958,82	4810761,41
Quercus robur	177823,42	4822741,70
Quercus robur	153633,35	4807424,08
Quercus robur	154353,47	4817410,47
Quercus robur	163619,22	4806704,63
Quercus robur	173603,90	4805985,33
Quercus robur	183587,39	4805266,16
Quercus robur	184307,08	4815248,99
Quercus robur	186026,21	4825159,53
Quercus robur	188012,09	4810967,97
Quercus robur	191294,72	4814744,83
Quercus robur	194711,67	4806471,56
Quercus robur	195143,39	4812460,48
Quercus robur	195359,41	4815454,91
Quercus robur	198076,48	4825291,99
Quercus robur	202552,75	4803900,14
Quercus robur	233072,33	4480847,33
Quercus robur	240736,64	4490349,49
Quercus robur	241734,38	4490281,46
Quercus robur	241802,42	4491279,20
Quercus robur	242256,22	4483229,35
Quercus robur	229800,66	4679591,86
Quercus robur	194289,24	4814528,78
Quercus robur	203550,81	4803828,26
Quercus robur	204990,81	4823789,02
Quercus robur	231076,61	4480983,16
Quercus robur	239699,10	4460348,83
Quercus robur	214269,61	4672663,68
Quercus robur	224248,90	4671960,82
Quercus robur	219219,71	4742519,64
Quercus robur	219931,68	4752498,52
Quercus robur	232769,21	4791695,42
Quercus robur	246951,12	4793684,07
Quercus robur	216256,87	4826987,37
Quercus robur	227437,91	4815146,37
Quercus robur	231140,96	4810867,49
Quercus robur	233435,70	4828754,05
Quercus robur	235214,61	4825616,75
Quercus robur	236345,29	4813500,73
Quercus robur	239205,17	4825328,26
Quercus robur	240058,56	4823260,93
Quercus robur	245899,84	4820833,31

Quercus robur	247606,74	4816699,11
Quercus robur	250455,26	4814488,11
Quercus robur	252721,00	4790261,26
Quercus robur	213530,74	4803109,52
Quercus robur	214250,00	4813088,78
Quercus robur	224948,92	4822346,93
Quercus robur	244182,24	4810929,80
Quercus robur	251308,79	4812421,17
Quercus robur	252090,44	4809356,81
Quercus robur	253026,45	4822323,80
Quercus robur	253438,54	4800235,90
Quercus robur	253725,90	4804225,72
Quercus robur	256511,95	4815053,63
Quercus robur	257000,00	4780000,00
Quercus robur	279000,00	4795000,00
Quercus robur	257000,00	4815000,00
Quercus robur	259000,00	4808000,00
Quercus robur	260000,00	4818000,00
Quercus robur	264000,00	4810000,00
Quercus robur	265000,00	4818000,00
Quercus robur	266000,00	4825000,00
Quercus robur	269000,00	4814000,00
Quercus robur	271000,00	4822000,00
Quercus robur	271000,00	4833000,00
Quercus robur	280000,00	4450000,00
Quercus robur	260000,00	4790000,00
Quercus robur	270000,00	4760000,00
Quercus robur	270000,00	4790000,00
Quercus robur	280000,00	4740000,00
Quercus robur	290000,00	4790000,00
Quercus robur	250000,00	4810000,00
Quercus robur	260000,00	4800000,00
Quercus robur	260000,00	4810000,00
Quercus robur	264000,00	4810000,00
Quercus robur	265000,00	4818000,00
Quercus robur	266000,00	4825000,00
Quercus robur	269000,00	4814000,00
Quercus robur	271000,00	4822000,00
Quercus robur	271000,00	4833000,00
Quercus robur	272000,00	4812000,00
Quercus robur	275000,00	4809000,00
Quercus robur	278000,00	4801000,00
Quercus robur	288000,00	4811000,00
Quercus robur	288000,00	4822000,00
Quercus robur	290000,00	4804000,00
Quercus robur	331000,00	4789000,00
Quercus robur	270000,00	4820000,00
Quercus robur	270000,00	4830000,00
Quercus robur	280000,00	4820000,00
Quercus robur	290000,00	4810000,00

Quercus robur	300000,00	4770000,00
Quercus robur	300000,00	4780000,00
Quercus robur	300000,00	4790000,00
Quercus robur	310000,00	4770000,00
Quercus robur	330000,00	4780000,00
Quercus robur	344000,00	4796000,00
Quercus robur	300000,00	4800000,00
Quercus robur	310000,00	4810000,00
Quercus robur	320000,00	4800000,00
Quercus robur	320000,00	4810000,00
Quercus robur	330000,00	4800000,00
Quercus robur	330000,00	4810000,00
Quercus robur	332000,00	4802000,00
Quercus robur	360000,00	4800000,00
Quercus robur	383000,00	4804000,00
Quercus robur	385000,00	4802000,00
Quercus robur	385000,00	4804000,00
Quercus robur	387000,00	4802000,00
Quercus robur	389000,00	4804000,00
Quercus robur	390000,00	4800000,00
Quercus robur	401000,00	4787000,00
Quercus robur	400000,00	4790000,00
Quercus robur	417000,00	4762000,00
Quercus robur	410000,00	4780000,00
Quercus robur	420000,00	4794000,00
Quercus robur	421000,00	4794000,00
Quercus robur	431000,00	4794000,00
Quercus robur	434000,00	4784000,00
Quercus robur	430000,00	4770000,00
Quercus robur	430000,00	4790000,00
Quercus robur	440000,00	4790000,00
Quercus robur	450000,00	4790000,00
Quercus robur	470000,00	4790000,00
Quercus robur	479000,00	4790000,00
Quercus robur	481000,00	4779000,00
Quercus robur	486000,00	4773000,00
Quercus robur	486000,00	4795000,00
Quercus robur	480000,00	4770000,00
Quercus robur	487000,00	4792000,00
Quercus robur	490000,00	4735000,00
Quercus robur	492000,00	4789000,00
Quercus robur	496000,00	4776000,00
Quercus robur	400000,00	4800000,00
Quercus robur	420000,00	4800000,00
Quercus robur	430000,00	4800000,00
Quercus robur	430000,00	4801000,00
Quercus robur	430000,00	4810000,00
Quercus robur	440000,00	4800000,00
Quercus robur	440000,00	4810000,00
Quercus robur	452000,00	4814000,00

Quercus robur	460000,00	4810000,00
Quercus robur	466000,00	4802000,00
Quercus robur	470000,00	4800000,00
Quercus robur	477000,00	4800000,00
Quercus robur	486000,00	4800000,00
Quercus robur	490000,00	4800000,00
Quercus robur	501000,00	4766000,00
Quercus robur	500000,00	4750000,00
Quercus robur	505000,00	4765000,00
Quercus robur	500000,00	4760000,00
Quercus robur	506000,00	4768000,00
Quercus robur	500000,00	4770000,00
Quercus robur	510000,00	4750000,00
Quercus robur	510000,00	4760000,00
Quercus robur	517000,00	4771000,00
Quercus robur	510000,00	4790000,00
Quercus robur	520000,00	4750000,00
Quercus robur	520000,00	4760000,00
Quercus robur	520000,00	4770000,00
Quercus robur	520000,00	4790000,00
Quercus robur	526000,00	4760000,00
Quercus robur	530000,00	4751000,00
Quercus robur	535000,00	4740000,00
Quercus robur	535000,00	4758000,00
Quercus robur	535000,00	4782000,00
Quercus robur	536000,00	4761000,00
Quercus robur	530000,00	4770000,00
Quercus robur	530000,00	4780000,00
Quercus robur	538000,00	4722000,00
Quercus robur	540000,00	4753000,00
Quercus robur	540000,00	4771000,00
Quercus robur	540000,00	4720000,00
Quercus robur	540000,00	4750000,00
Quercus robur	540000,00	4760000,00
Quercus robur	540000,00	4780000,00
Quercus robur	552000,00	4762000,00
Quercus robur	552000,00	4763000,00
Quercus robur	550000,00	4740000,00
Quercus robur	550000,00	4750000,00
Quercus robur	550000,00	4760000,00
Quercus robur	562000,00	4780000,00
Quercus robur	560000,00	4740000,00
Quercus robur	560000,00	4770000,00
Quercus robur	560000,00	4780000,00
Quercus robur	560000,00	4790000,00
Quercus robur	571000,00	4756000,00
Quercus robur	573000,00	4750000,00
Quercus robur	570000,00	4750000,00
Quercus robur	581000,00	4792000,00
Quercus robur	584000,00	4778000,00

Quercus robur	580000,00	4760000,00
Quercus robur	580000,00	4770000,00
Quercus robur	580000,00	4780000,00
Quercus robur	589000,00	4759000,00
Quercus robur	590000,00	4761000,00
Quercus robur	592000,00	4795000,00
Quercus robur	590000,00	4750000,00
Quercus robur	590000,00	4760000,00
Quercus robur	596000,00	4791000,00
Quercus robur	590000,00	4780000,00
Quercus robur	590000,00	4790000,00
Quercus robur	599000,00	4772000,00
Quercus robur	599000,00	4795000,00
Quercus robur	519000,00	4806000,00
Quercus robur	510000,00	4800000,00
Quercus robur	519000,00	4806000,00
Quercus robur	520000,00	4800000,00
Quercus robur	520000,00	4810000,00
Quercus robur	525000,00	4801000,00
Quercus robur	526000,00	4803000,00
Quercus robur	593000,00	4802000,00
Quercus robur	597000,00	4804000,00
Quercus robur	633000,00	4645000,00
Quercus robur	601000,00	4761000,00
Quercus robur	602000,00	4791000,00
Quercus robur	602000,00	4794000,00
Quercus robur	604000,00	4784000,00
Quercus robur	604000,00	4786000,00
Quercus robur	600000,00	4750000,00
Quercus robur	605000,00	4764000,00
Quercus robur	605000,00	4785000,00
Quercus robur	600000,00	4760000,00
Quercus robur	600000,00	4780000,00
Quercus robur	600000,00	4790000,00
Quercus robur	610000,00	4750000,00
Quercus robur	610000,00	4760000,00
Quercus robur	610000,00	4770000,00
Quercus robur	620000,00	4770000,00
Quercus robur	620000,00	4780000,00
Quercus robur	630000,00	4760000,00
Quercus robur	638000,00	4770000,00
Quercus robur	638000,00	4772000,00
Quercus robur	969776,90	4625326,68
Quercus robur	969077,95	4635329,32
Quercus robur	968377,77	4645331,79
Quercus robur	974359,14	4631677,84
Quercus robur	964858,65	4695341,63
Quercus robur	964151,18	4705343,10
Quercus robur	975567,22	4686045,73
Quercus robur	975567,22	4686045,73
