# Supplementary material for: Late Quaternary range shifts of marcescent oaks unveil the dynamics of a major biogeographic transition in southern Europe
Source: Sci Rep. 2020 Dec 9;10:21598. doi: 10.1038/s41598-020-78576-9 (PMC7726089; doi:10.1038/s41598-020-78576-9)
Supplement: Supplementary file 4 — Supplementary Table S3. [file 41598_2020_78576_MOESM4_ESM.pdf]

Table S3 – Variables used in SDMs for each target species (BIO1 = Annual Mean Temperature; BIO3 = Isothermality; BIO6 = Min Temperature of Coldest Month; BIO7 = Temperature Annual Range; BIO11 = Mean Temperature of Coldest Quarter; BIO12 = Annual Precipitation; BIO15 = Precipitation Seasonality; BIO18 = Precipitation of Warmest Quarter; BIO19 = Precipitation of Coldest Quarter; TRI_AVG – Topographic Ruggedness Index average; TWI_STD – Topographic Wetness Index standard-deviation).

Species	Temperature related Bioclimatic variables					Precipitation related Bioclimatic variables				Topographic variables	
	**BIO_01**	**BIO_03**	**BIO_06**	**BIO_07**	**BIO_11**	**BIO_12**	**BIO_15**	**BIO_18**	**BIO_19**	**TRI_AVG**	**TWI_STD**
Q. broteroi	✓				✓	✓			✓	✓	✓
Q. canariensis	✓	✓				✓		✓		✓	✓
Q. coutinhoi	✓				✓	✓		✓		✓	✓
Q. estremadurensis	✓				✓	✓		✓		✓	✓
Q. faginea	✓			✓			✓	✓		✓	✓
Q. lusitanica		✓	✓			✓		✓		✓	✓
Q. marianica	✓				✓	✓		✓		✓	✓
Q. robur	✓		✓				✓	✓		✓	✓
