# Supplementary figures and images for: Late Quaternary range shifts of marcescent oaks unveil the dynamics of a major biogeographic transition in southern Europe
Source: Sci Rep. 2020 Dec 9;10:21598. doi: 10.1038/s41598-020-78576-9 (PMC7726089; doi:10.1038/s41598-020-78576-9)

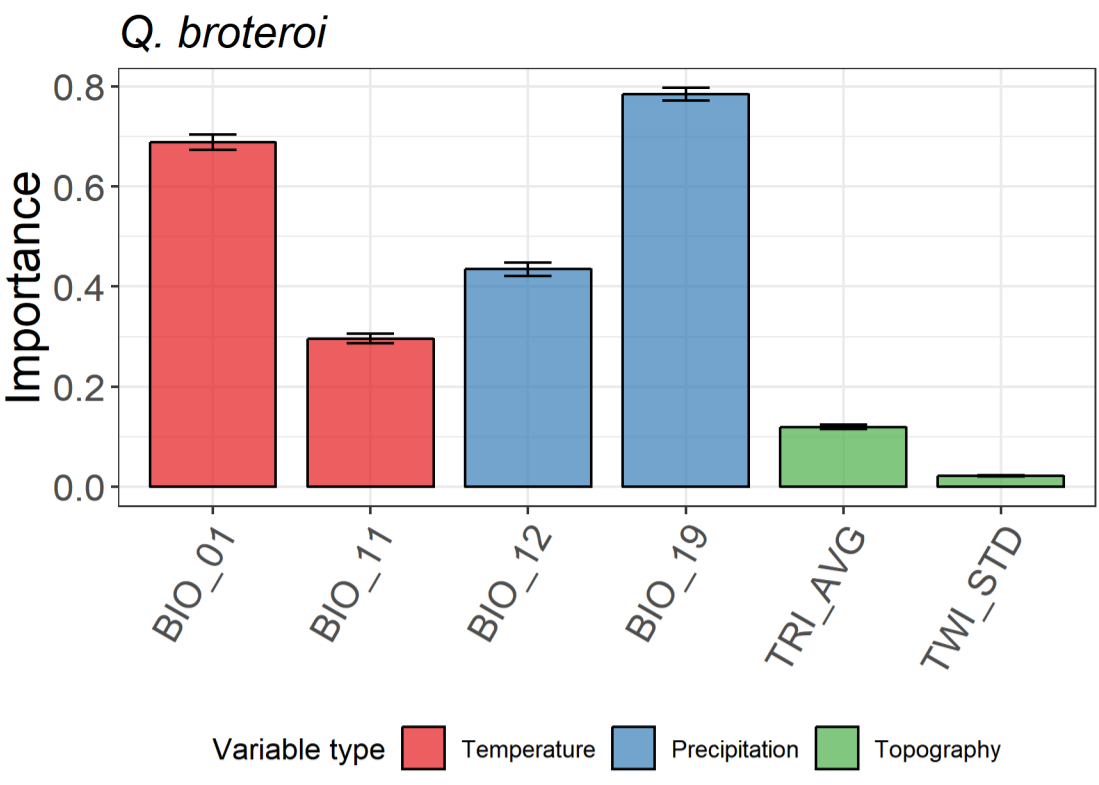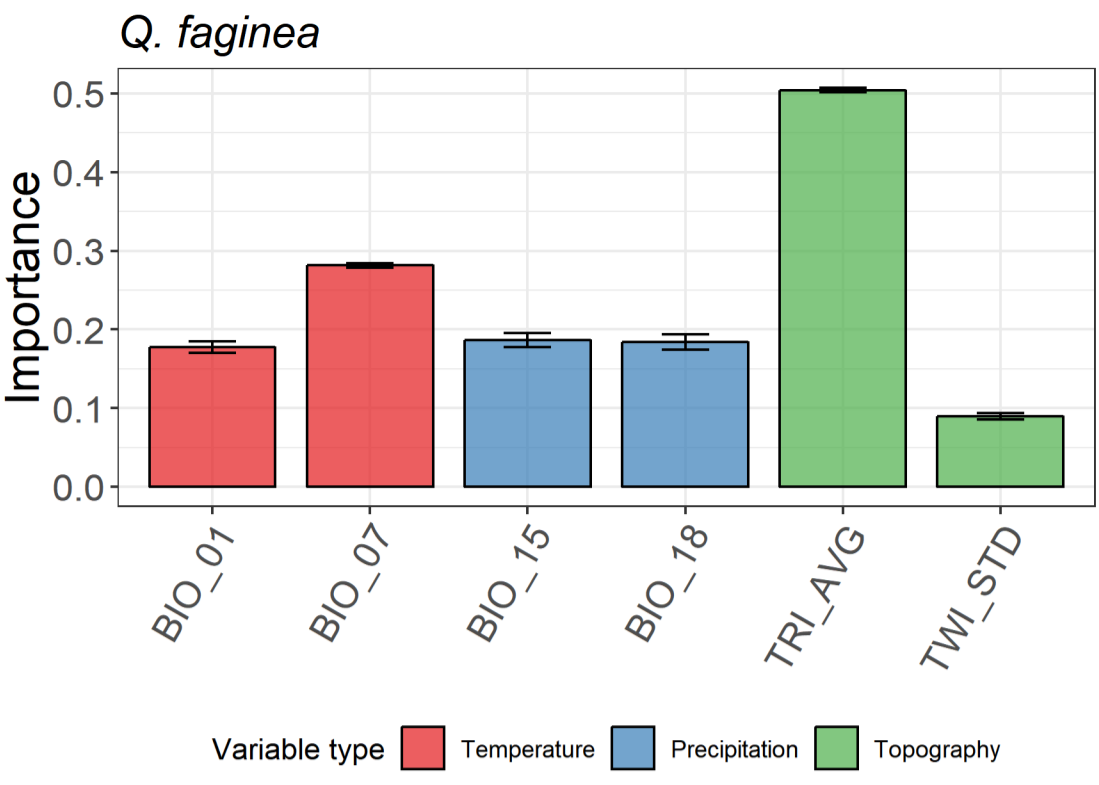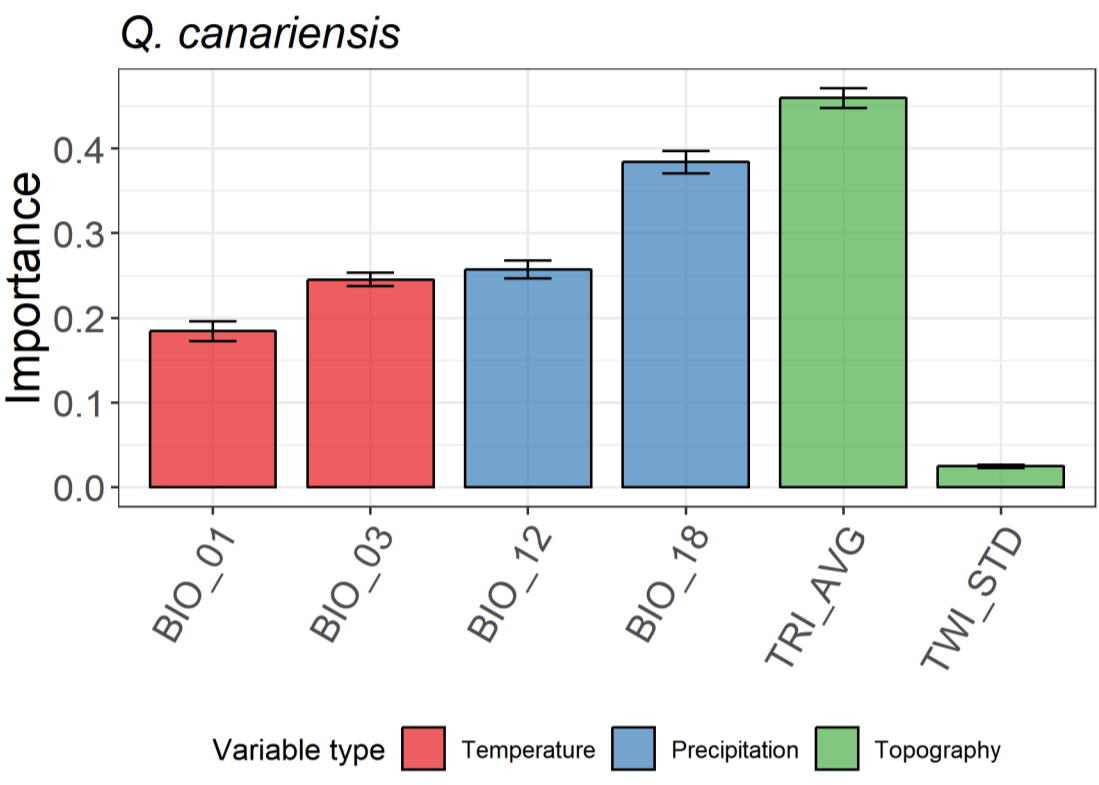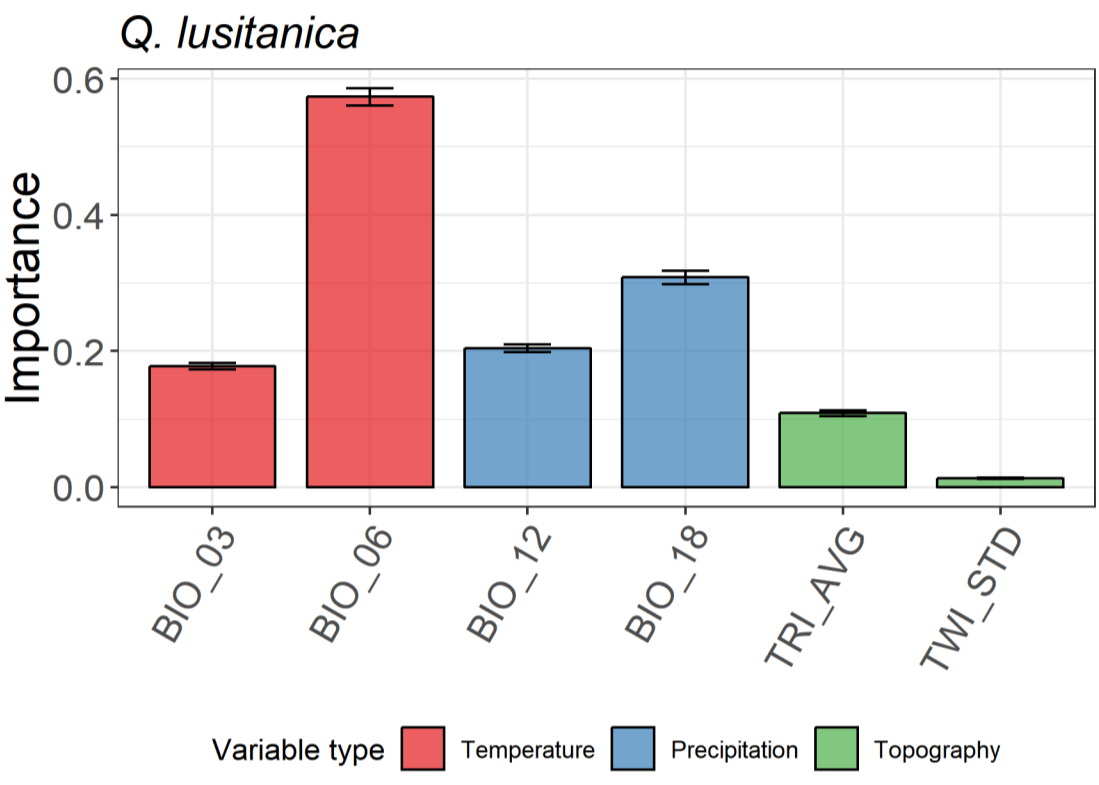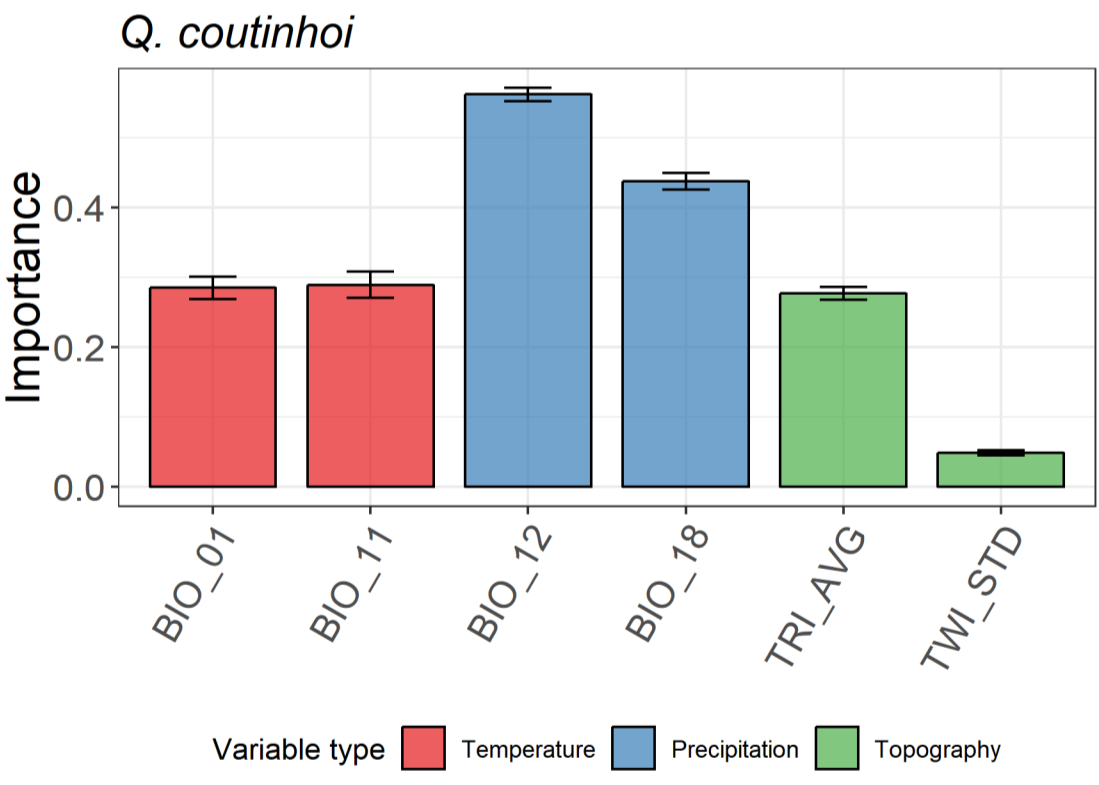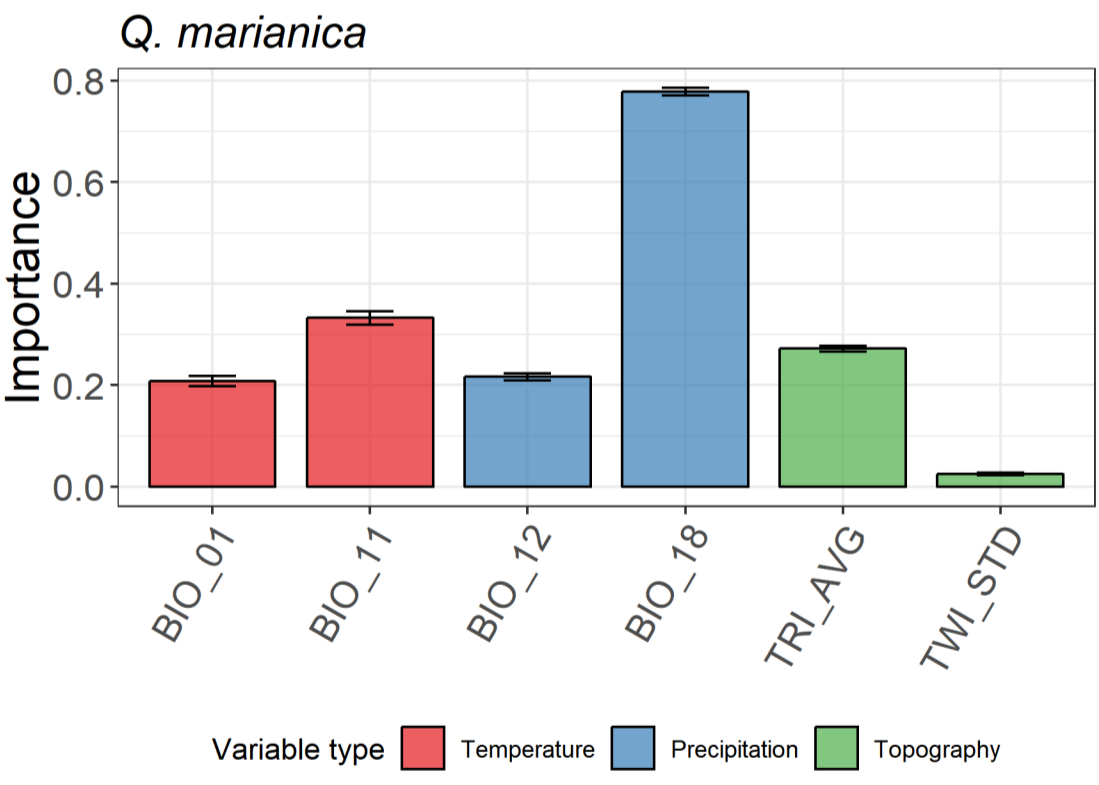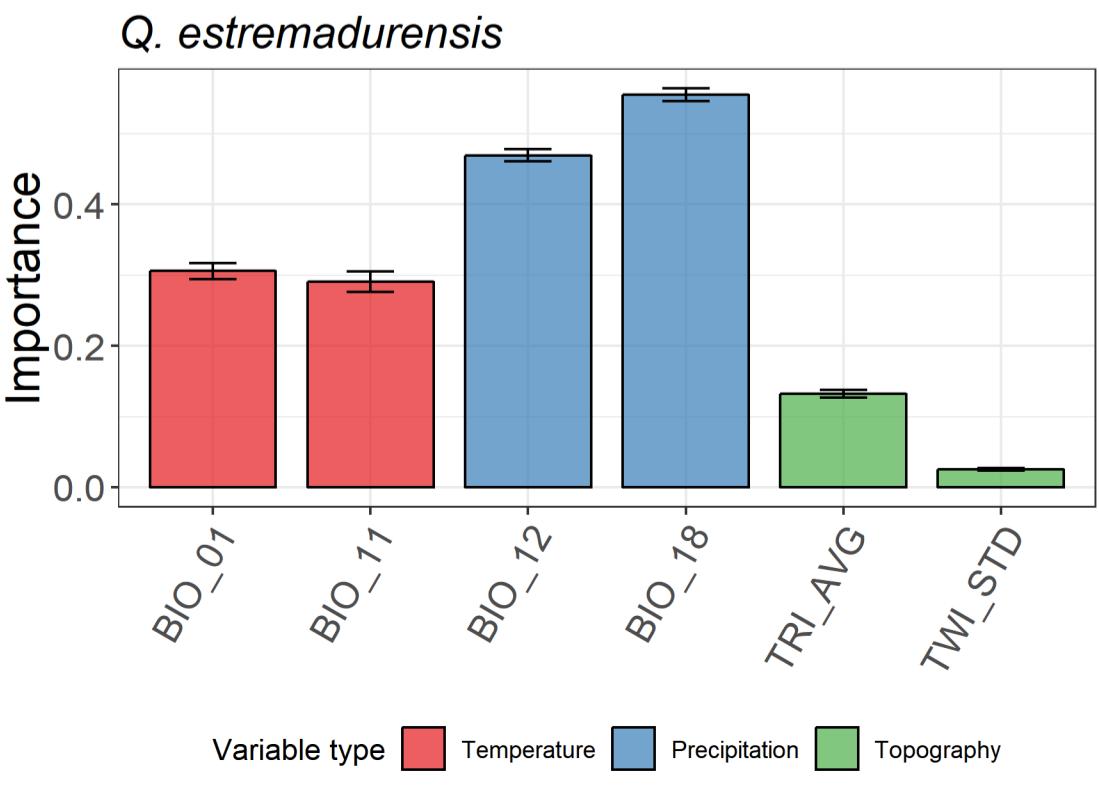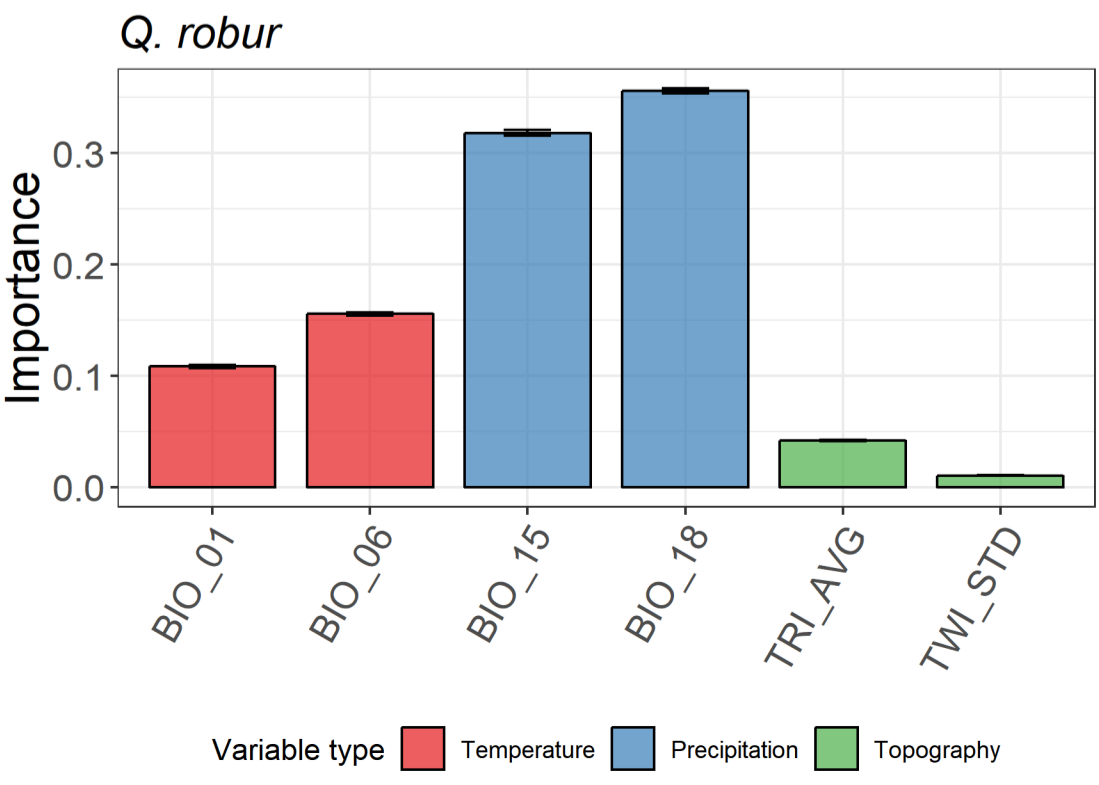

Supplement: Supplementary file 5 — Supplementary Fig. S1. [file 41598_2020_78576_MOESM5_ESM.pdf]

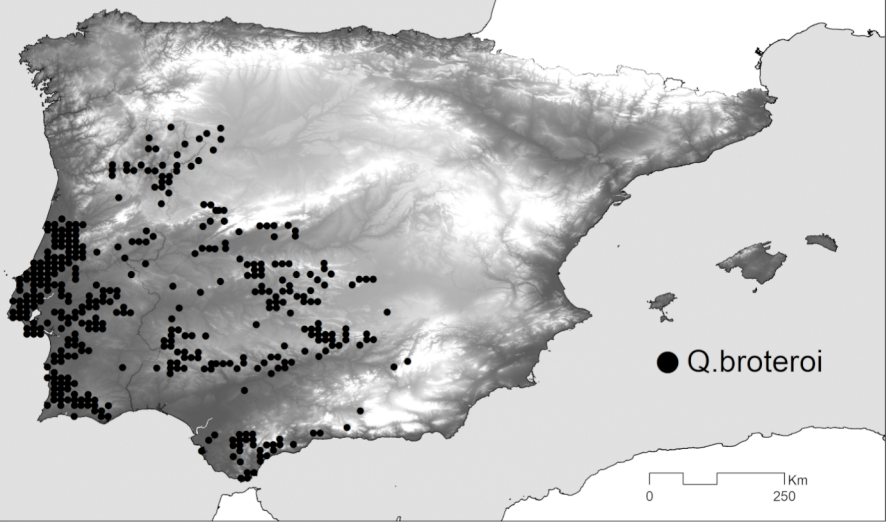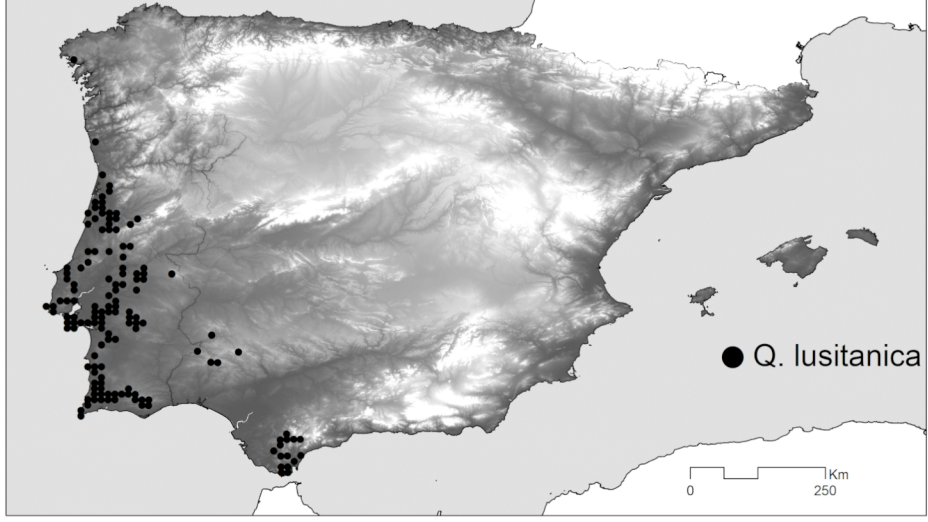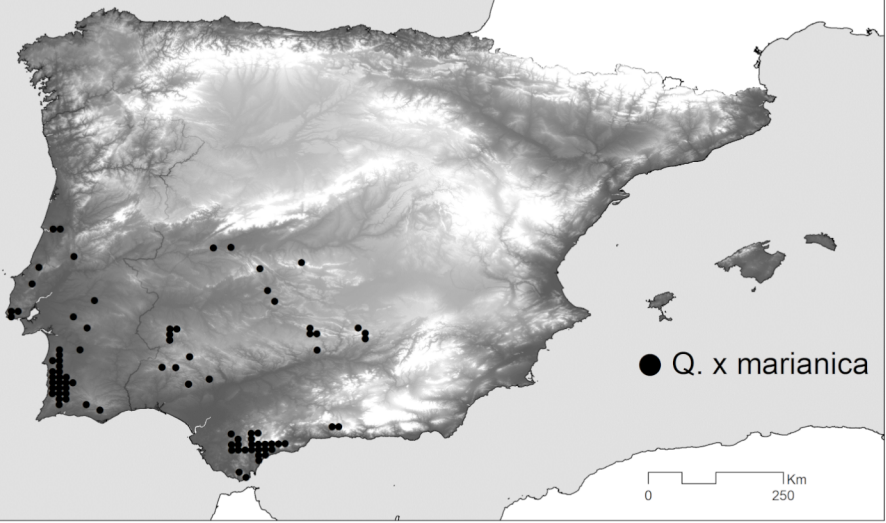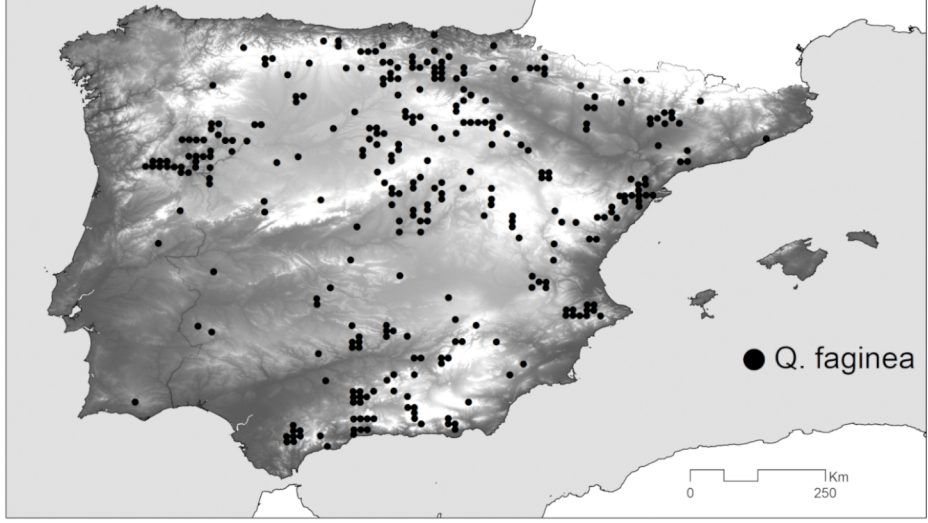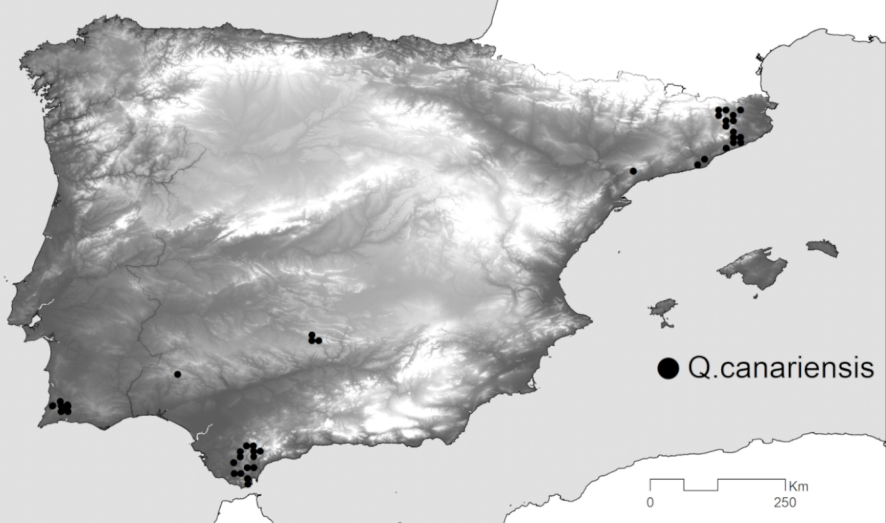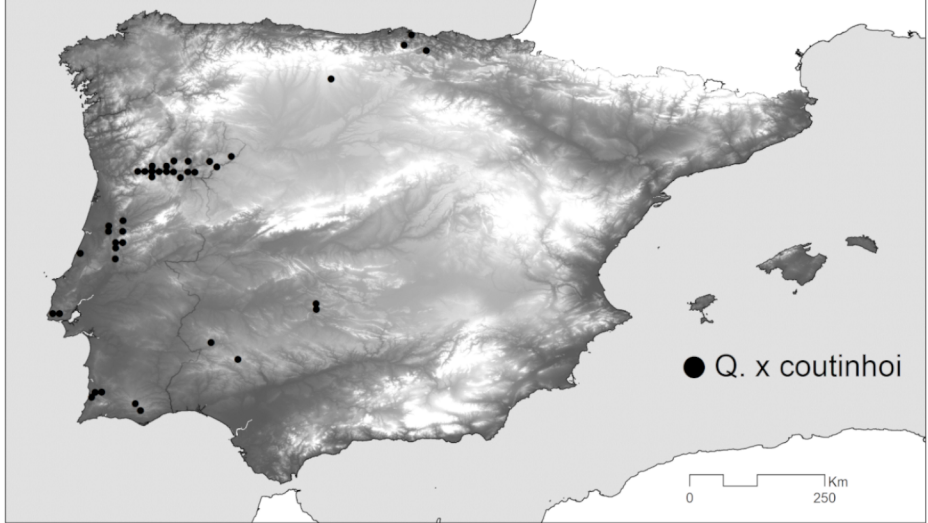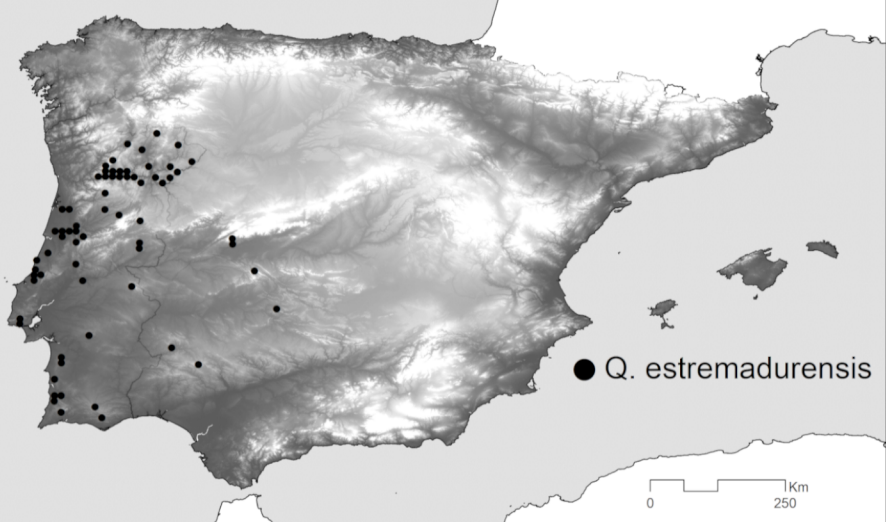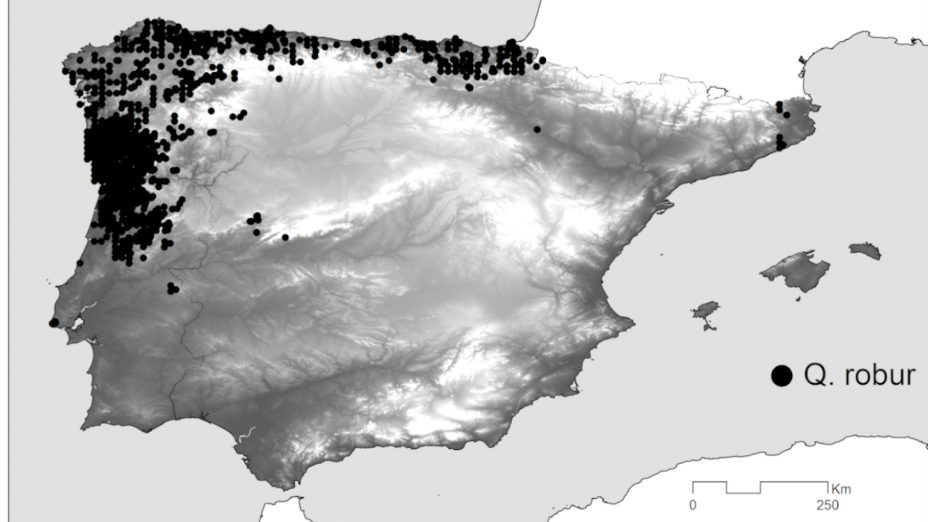

Supplement: Supplementary file 6 — Supplementary Fig. S2. [file 41598_2020_78576_MOESM6_ESM.pdf]

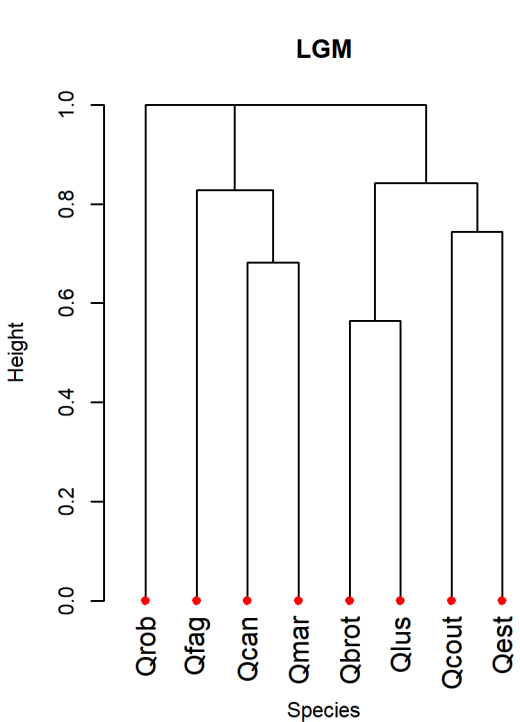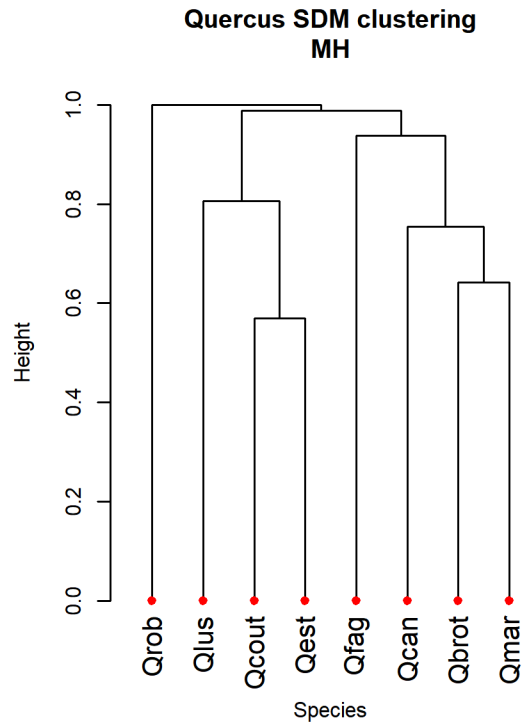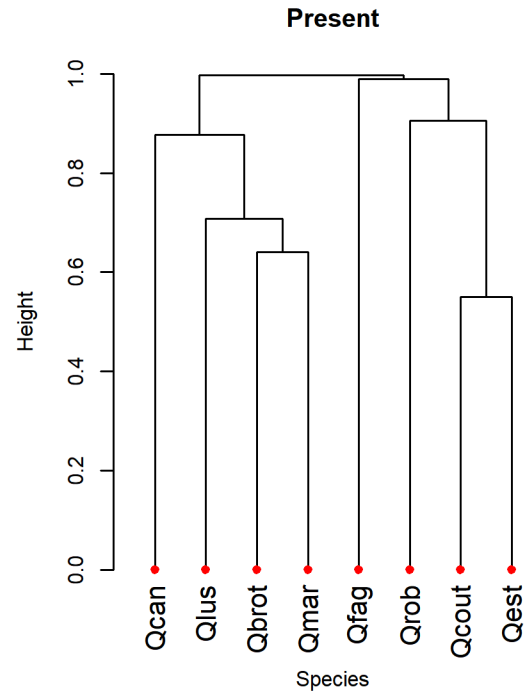

Supplement: Supplementary file 7 — Supplementary Fig. S3. [file 41598_2020_78576_MOESM7_ESM.pdf]
