# Supplementary material for: Late Quaternary range shifts of marcescent oaks unveil the dynamics of a major biogeographic transition in southern Europe
Source: Sci Rep. 2020 Dec 9;10:21598. doi: 10.1038/s41598-020-78576-9 (PMC7726089; doi:10.1038/s41598-020-78576-9)
Supplement: Supplementary file 8 — Supplementary Note S1. [file 41598_2020_78576_MOESM8_ESM.pdf]

Late Quaternary range shifts of marcescent oaks unveil the dynamics of a major biogeographic transition in southern Europe

Carlos Vila-Viçosa[1,2,3*], João Gonçalves[1]; João Honrado[1,3]; Ângela Lomba A[1]; Rubim A. Silva[1,2,3]; Francisco Maria Vázquez [4]; Cristina Garcia[1,5]

Corresponding author: cvv@cibio.up.pt

Supplementary Note S1

Focal taxa and occurrence data

- *Quercus broteroi* (Cout.) Rivas Mart. & C. Sáenz and *Q. faginea* Lam. were formerly considered subspecies (of *Q. faginea*) but are considered nowadays as independent taxa by the majority of Iberian botanists and experts of genus *Quercus* [1-3], holding today a predominantly parapatric distribution. *Quercus broteroi* is indifferent for lithology and occupies areas with oceanic climate along the western coast , whereas *Q. faginea* is a strictly basophilous species, coping under sub-continental climates with cold winters [4].
- *Quercus canariensis* Willd. is a rare tree, disseminated in areas with moderate summer precipitation and/or fog condensation and mesic siliceous soils, being sympatric with *Q. broteroi* in its Southern distribution range [5].
- To deal with taxonomic interactions and biogeographic patterns we considered the hybrids *Q.* × *marianica* C. Vicioso (*Q. canariensis* × *Q. broteroi*) to ascertain the past occurrence of *Q. canariensis* in these forests, or the breakthrough of this hybrid, departed from parental species.
- Additionally, we used the reference of *Q. estremadurensis* O. Schwarz (≡*Q. robur* subsp. *estremadurensis* (O.Schwarz) A. Camus), a relictual taxa occurring in transitional areas towards Mediterranean conditions in Southern Iberian Peninsula, presenting marcescent behavior and considered a Tertiary relictual taxon [3,6,7].
- We add the Portuguese dwarf-oak (*Q. lusitanica* Lam.), as it shares synecological and biogeographical traits and belonging to the same infrageneric group of oaks (Subsect. *Gallifereae*) [8].

- Finally, we also used broad references to *Q.* × *coutinhoi* Samp, which includes all taxonomic interactions between *Q. faginea* or *Q. broteroi* and roburoid oaks (*Q. robur* and *Q. estremadurensis*), which hybrid swarms are known from central and Northern Iberia.
- We add *Q. robur* to the analysis as parent species, in order to establish the comparison with a deciduous and temperate species, ecologically more demanding in terms of summer precipitation.

1 Rivas-Martínez, S. & Saénz, C. Enumeración de los Quercus de la Península Ibérica. *Rivasgodaya* **6**, 101-110 (1991).

2 García-Mijangos, I., Campos, J. A., Biurrun, I., Herrera, M. & Loidi, J. in *Warm-Temperate Deciduous Forests around the Northern Hemisphere* (eds Elgene O Box & Kazue Fujiwara) 119-138 (Springer, 2015).

3 Vila-Viçosa, C., Vázquez F.M., Meireles C & Pinto-Gomes, C. Taxonomic peculiarities of marcescent oaks (Quercus, Fagaceae) in southern Portugal. *Lazaroa* **35**, 139–153, doi:10.5209/rev_LAZA.2014.v35.42555 (2014).

4 Franco, J. A. in *Flora Iberica, (Plantanaceae-Plumbaginaceae [Partim])* Vol. 2 (eds Castroviejo S. & et. al.) pp. 15-36 (Real Jardín Botánico, CSIC, 1990).

5 Vila-Viçosa, C. *et al.* Syntaxonomic update on the relict groves of Mirbeck's oak (Quercus canariensis Willd. and Q. marianica C. Vicioso) in southern Iberia. *Plant Biosystems* **149**, 512-526, doi:10.1080/11263504.2015.1040484 (2015).

6 Schwarz, O. Einige neue Eichen des Mediterrangebiets und Vorderasiens. *Notizblatt des Königl. botanischen Gartens und Museums zu Berlin* **114**, 461-469, doi:10.2307/3994977 (1935).

7 Vicioso, C. *Revisión del género Quercus en España*. (Tipografía Artítica, 1950).

8 Tschan, G. F. & Denk, T. Trichome types, foliar indumentum and epicuticular wax in the Mediterranean gall oaks, Quercus subsection Galliferae (Fagaceae): implications for taxonomy, ecology and evolution. *Botanical Journal of the Linnean Society* **169**, 611-644 (2012).
