# Supplementary material for: Late Quaternary range shifts of marcescent oaks unveil the dynamics of a major biogeographic transition in southern Europe
Source: Sci Rep. 2020 Dec 9;10:21598. doi: 10.1038/s41598-020-78576-9 (PMC7726089; doi:10.1038/s41598-020-78576-9)
Supplement: Supplementary file 9 — Supplementary Information S1. [file 41598_2020_78576_MOESM9_ESM.pdf]

Late Quaternary range shifts of marcescent oaks unveil the dynamics of a major biogeographic transition in southern Europe

Carlos Vila-Viçosa[1,2,3*], João Gonçalves[1]; João Honrado[1,3]; Ângela Lomba A[1]; Rubim A. Silva[1,2,3]; Francisco Maria Vázquez [4]; Cristina Garcia[1,5]

Corresponding author: cvv@cibio.up.pt

Supplmentary information S1 - Herbaria list of studied physical and virtual collections (*Quercus* L) and consulted databases

Studied herbaria collections *(Quercus* L.*)*

Herbarium Code	Institution	Location
AVE	Universidade de Aveiro	Portugal. Aveiro.
BCN	University of Barcelona	Spain. Catalonia. Barcelona
BRESA	Polytechnic Institute of Bragança - IPB	Portugal. Bragança.
COI	University of Coimbra	Portugal. Coimbra.
HSS	Research Centre of "La Orden-Valdesequera"	Spain. Extremadura. Mérida.
HVR	Universidade de Trás-os-Montes e Alto Douro	Portugal. Vila Real.
L	Naturalis	Netherlands. Leiden.
LISE		Portugal. Oeiras.
LISFA	Instituto Nacional de Investigação Agrária e Veterinária, I. P. (INIAV)	Portugal. Oeiras.
LISI	Instituto Superior de Agronomia	Portugal. Lisboa.
LISU	Museu Nacional de História Natural e da Ciência	Portugal. Lisboa.
MA	Real Jardín Botánico	Spain. Madrid.
PO	Museu de História Natural e da Ciência da Universidade do Porto	Portugal. Porto
SALA	Universidad de Salamanca	Spain. Salamanca.
SANT	Universidad de Santiago de Compostela	Spain. Galicia. Santiago de Compostela.
UEVH	Universidade de Évora	Portugal. Évora.

Online herbaria collections (*Quercus*)

Herbarium Code	Institution	Location
B	Botanischer Garten und Botanisches Museum Berlin-Dahlem, Zentraleinrichtung der Freien Universität Berlin	Germany. Berlin.
G	Conservatoire et Jardin botaniques de la Ville de Genève	Switzerland. Genève.
K	Royal Botanic Gardens	U.K. England. Kew.
MAIA	INIA (Instituto Nacional de Investigación y Tecnología Agraria y Alimentaria)	Spain. Madrid.
P	Muséum National d'Histoire Naturelle	France. Paris.
MPU	Université de Montpellier	France. Montpellier.

Online databases

Name	Site	Reference
ANTHOS: Spanish Plants Information System	http://www.anthos.es/	Castroviejo *et al.* (2006)
Flora-On: Flora de Portugal Interactiva	www.flora-on.pt	Pereira *et al.* (2016)
GBIF Global Biodiversity Information Facility	https://www.gbif.org/	Holstein (2001)
SIVIM: Iberian and Macaronesian Vegetation Information System	http://www.sivim.info/sivi/	Font *et al.* (2012)

References

Castroviejo, S., Aedo, C. & Medina, L. (2006) Management of floristic information on the Internet: the Anthos solution. *Willdenowia*, *36*, 127-137.

Font, X., Pérez-García, N., Biurrun, I., Fernández-González, F. & Lence, C. (2012) The Iberian and Macaronesian Vegetation Information System (SIVIM, www. sivim. info), five years of online vegetation's data publishing. *Plant Sociology*, *49*, 89-95.

Holstein, J. (2001) *GBIF: Global Biodiversity Information Facility*. University of Ulm.

Pereira, A.J., Francisco, A. & Porto, M. (2016) Flora-On: Occurrence data of the vascular flora of mainland Portugal. *PhytoKeys*, 105.
